# Diversification of Pharmaceutical Manufacturing Processes: Taking the Plunge into the Non-PGM Catalyst Pool

**DOI:** 10.1021/acscatal.4c01809

**Published:** 2024-06-13

**Authors:** Hui Zhao, Anne K. Ravn, Michael C. Haibach, Keary M. Engle, Carin C. C. Johansson Seechurn

**Affiliations:** ∥ Sinocompound Catalysts, Building C, Bonded Area Technology Innovation Zone, Zhangjiagang, Jiangsu 215634, China; ‡ Department of Chemistry, The Scripps Research Institute, 10550 North Torrey Pines Road, La Jolla, California 92037, United States; § Process Research and Development, AbbVie Inc., 1 North Waukegan Road, North Chicago, Illinois 60064, United States; † Sinocompound U.K., 14b Warwick Road, Barnet, EN5 5EQ, U.K.

**Keywords:** nonplatinum group metals, pharmaceutical industry, earth-abundant metals, catalysis, sustainability

## Abstract

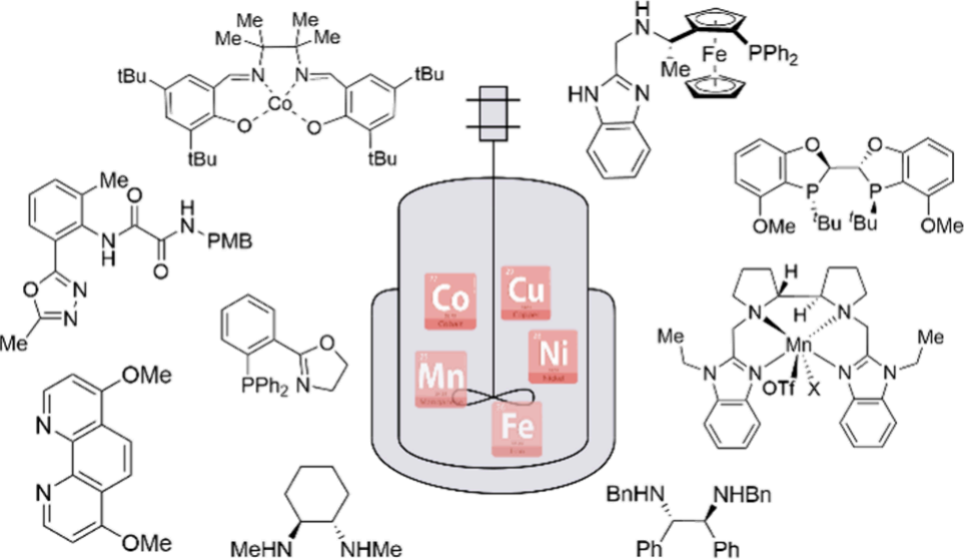

Recent global events have led to the cost of platinum group metals (PGMs) reaching unprecedented heights. Many chemical companies are therefore starting to seriously consider and evaluate if and where they can substitute PGMs for non-PGMs in their catalytic processes. This review covers recent highly relevant applications of non-PGM catalysts in the modern pharmaceutical industry. By highlighting these selected successful examples of non-PGM-catalyzed processes from the literature, we hope to emphasize the enormous potential of non-PGM catalysis and inspire further development within this field to enable this technology to progress toward manufacturing processes. We also present some historical contexts and review the perceived advantages and challenges of implementing non-PGM catalysts in the pharmaceutical manufacturing environment.

## 1 Introduction

It is well-established that nonprecious metal^1^ catalysis has been widely utilized since the beginning of the twentieth century for large-scale industrial processes in the petrochemical, food, and fine chemical industries. In addition, essential biochemical transformations in living organisms take advantage of enzymes containing base metal ions, such as iron and magnesium.^2^ These examples stand in stark contrast to the state of play in the pharmaceutical industry, wherein platinum group metal (PGM) catalysis is heavily relied upon and adoption of non-PGM alternatives is not yet widespread.

**Figure 1:**
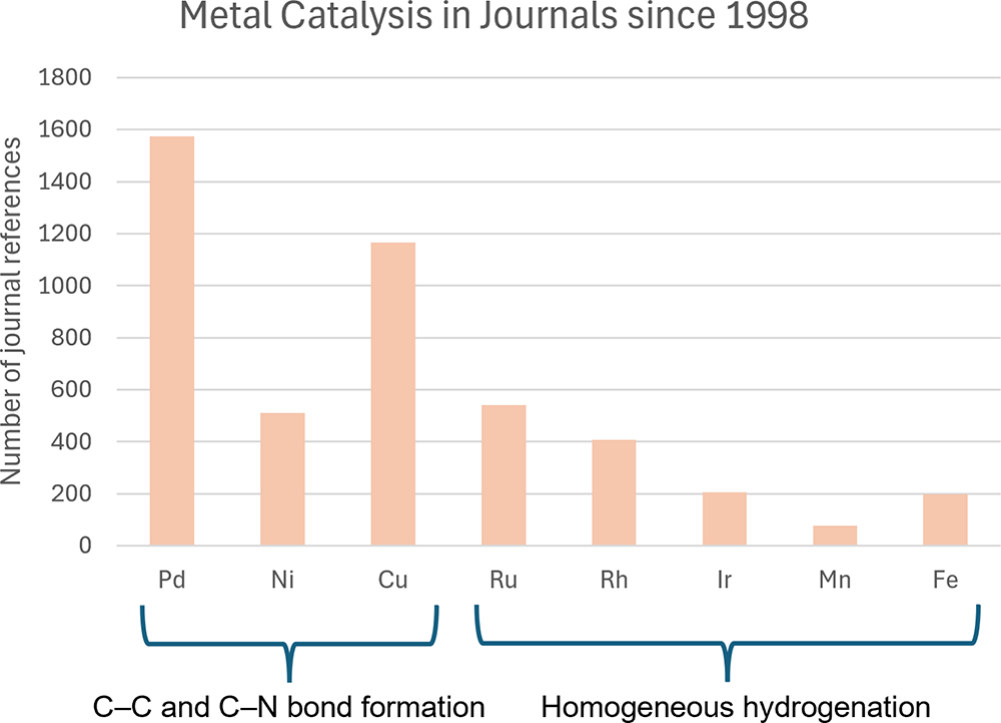
A number of journal publications involving metal catalysis published since 1998. Data from SciFinder (search terms: “metal” AND “C–C/C–N bond formation” for Pd, Ni, Cu, and “metal” AND “homogeneous hydrogenation” for Ru, Rh, Ir, Mn, Fe).

The late twentieth century witnessed a rise in processes catalyzed by platinum group metals (PGMs) in the pharmaceutical industry to construct frameworks of key intermediates that could not be obtained by traditional synthetic methodology. Subsequently, during the last two decades, PGM catalysis has made substantial contribution to the synthesis of compound libraries in batch as well as flow production, lead optimization, process chemistry and ultimately the large-scale preparation of APIs (active pharmaceutical ingredients) with high efficiency.^3^ Indeed, the cherry on the cake for PGM-catalyzed reactions has been recognition with multiple Nobel Prizes in chemistry—for asymmetric hydrogenation and oxidation, olefin metathesis, and cross-coupling reactions.^4^ Because catalysts composed of palladium, rhodium, iridium, platinum, and ruthenium play an important role in the above transformations, it is unsurprising that precious metal catalysts have been selected as more general choices for metal-catalyzed reactions since the 1970s. Today, this trend continues, as indicated by the number of journal publications involving metal catalysis since 1998 (Figure 1), with the most frequently investigated metal being palladium for C–C and C–N bond formation, and ruthenium and rhodium for hydrogenation reactions.

It is interesting to consider that historically, both palladium and nickel catalysts were reported in early examples of C–C cross-coupling reactions. Why did subsequent research for pharmaceutical applications of these reactions focus more heavily on palladium? In Negishi’s report of the very first Negishi reaction in 1977, both Ni(PPh_3_)_4_ and PdCl_2_(PPh_3_)_2_ were successful catalysts for aryl–aryl bond formation. What may have tipped the balance in favor of palladium over nickel could have been a combination of factors, the importance of which slowly emerged over time. First, a key consideration is the need for high functional group tolerance due to the structural complexity of many drugs. The Kumada coupling (1970) is a classic example of a highly efficient Ni-catalyzed reaction with limited functional group tolerance. Second, the ease in which phosphine ligands can be tuned for PGMs, such as palladium, rhodium, and ruthenium, to engender desired reactivity and selectivity profiles proved to be highly enabling; as the importance of ancillary ligands became increasingly understood, the size of the phosphine ligand libraries available for these metals grew very rapidly. Third, an issue that has emerged in relatively recent years stems from the competitive timelines in the pharmaceutical industry combined with the often costly nature of the substrates, particularly in the early drug development phases. R&D and management often face the choice between using a cheaper, but less well-known, process with a non-PGM catalyst, or the use of a more conventional, but more costly, process using a PGM catalyst. Due to the higher potential risk of batch failure when the less conventional (non-PGM) process is used, in many cases the higher cost associated with the PGM-based route can be justified during R&D. Non-PGM catalyzed reactions have less precedence on large scale and are more complex and less understood, which makes quick trouble-shooting more difficult.

Another contributing factor to palladium being heavily favored over nickel for cross-coupling applications in industry has been the availability of readily accessible catalyst precursors. Nickel in particular suffered due to the instability, high cost, and toxicity associated with Ni(COD)_2_. The relatively slow uptake of nickel-catalyzed processes in industry may have, in turn, influenced academic research efforts to some extent. This effect can arguably also be seen in the massive difference in the range of commercially available phosphine ligands versus that of nitrogen-donor ligands, which in turn presents further challenges to the application of certain nickel- and copper-catalyzed processes.

Although the success and synthetic advantages of the use of PGM catalysis are evident, gradually, shortcomings, such as low abundance, high price, and supply insecurity of these precious metals, have become increasingly pertinent considerations, especially with the mounting strain of the global resources and global supply chain. From this point of view, nonprecious metal catalysts seem like obvious alternatives due to their advantages over precious counterparts from many practical perspectives (cost, abundance, sustainability, and waste streams^5^).^6^

**Figure 2:**
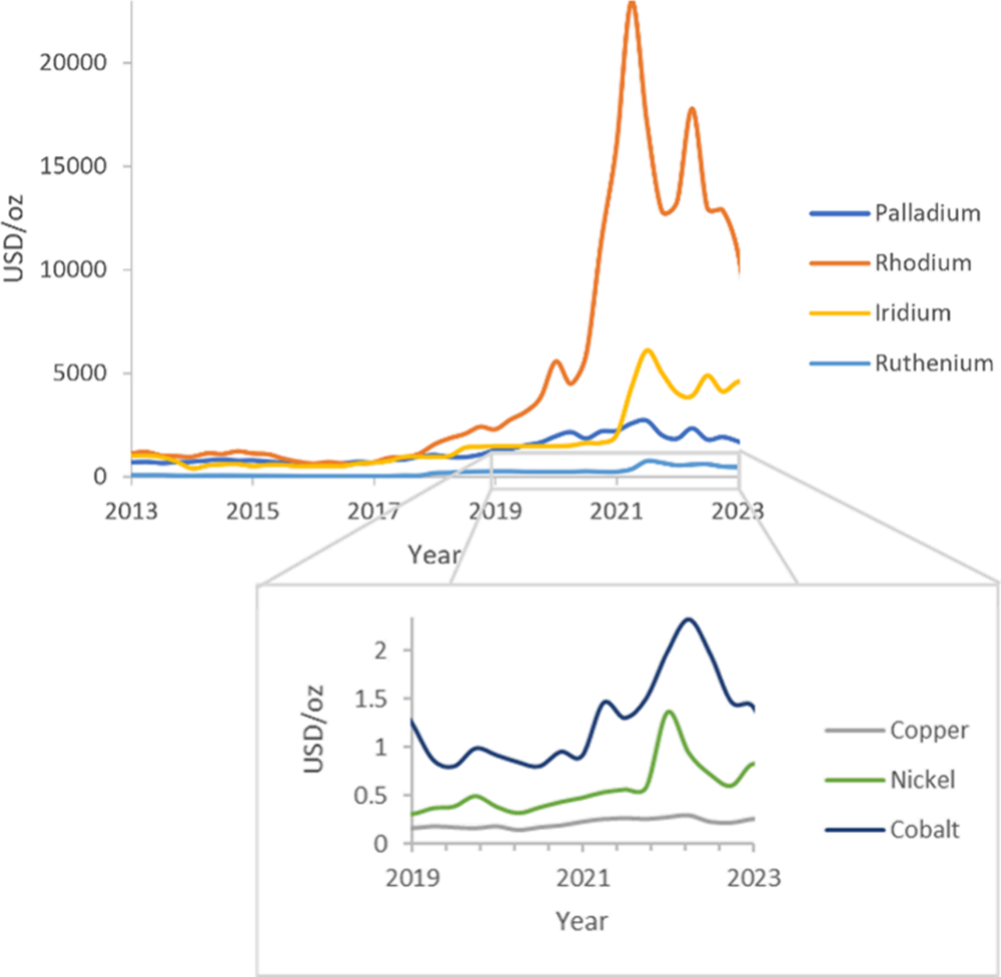
Cost graphs of selected metals over the past 10 years.^14^

As a matter of fact, nonprecious metal catalysts have resurfaced in recent years to provide alternatives to precious metals for transformations such as cross-couplings,^7^ hydrogenations,^8^ oxidations,^9^ aminations,^10^ alkoxylations,^11^ hydrosilylations,^12^ and C–H activations.^13^ One of the drivers, if not the main driver, behind this surge of papers is the recent spike in metal prices. As shown in Figure 2, recent global events have caused some metal prices to skyrocket, and unfortunately, there is no telling whether the price will ever return to the reasonably stable levels prior to 2018. In any case, this has served as a warning signal and spurred researchers on to find potential alternative methods, particularly for transformations that previously relied on PGM-catalysis only and especially for progression into scale-up and manufacturing.

**Figure 3:**
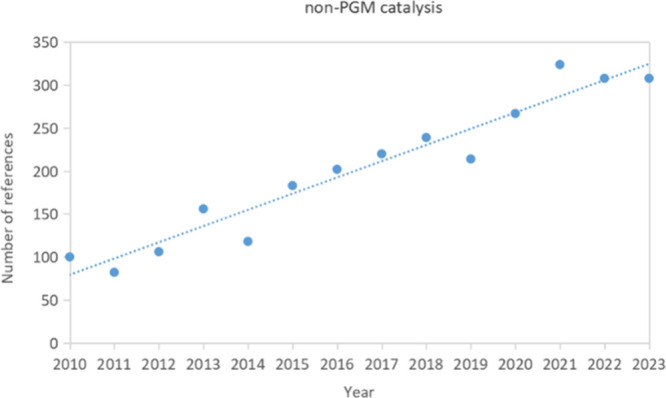
Number of references involving non-PGM metal catalysis increased during the last decades. Data were from SciFinder.

The trend of recent interest in non-PGM metal catalysis seems to be common ground for academic and industrial researchers, although, as with most “new” technologies, surprisingly few have found their way into pharmaceutical scale-up or manufacturing processes. The number of literature reports disclosed by academic groups far outweighs the reports on successful translation of these emerging methodologies to an industry-based user. As shown in Figure 3, the number of literature reports involving non-PGM catalysis increased steadily during the past decade. In 2019, over 16,000 references involving non-PGMs were published. This may be due in part to the availability of research funding. One academic researcher commented a few years back that “it is increasingly difficult to get funding applications approved for proposals that involve PGM catalysis”. The industrial chemist, in his/her turn, faces a number of different challenges, such as robustness, issues with workup, and cost, to proceed via the route less traveled compared to sticking with reliable (but increasingly less sustainable) PGM catalysis.

A thorough review article of non-PGM publication statistics recently appeared as a preprint, and the interested reader is referred to this publication for more in-depth analysis.^15^

Although the study of non-PGM catalysis is flourishing in academia, the adoption of these methods in the pharmaceutical industry faces several challenges. These include less well elucidated mechanistic pathways, risk aversion to replace known catalysts with less familiar systems, and the difficulty in sourcing some non-PGM catalysts on a large scale. Another challenge is that some non-PGM publications require the use of relatively expensive ligands, driving up the overall process cost compared to that of a potentially competing PGM-catalyzed process. As a hypothetical example, the ligand cost difference would likely outweigh the metal cost difference when comparing BINAP-Ru with DuPhos-Ni for an asymmetric hydrogenation if the ruthenium loading can be low enough.

There can also be key limits in the substrate scope of non-PGM publications. Sometimes, this methodology may not tolerate electron-rich aryl halides, sterically hindered nucleophiles, or substrates with Lewis-basic functional groups. Non-PGM-catalyzed processes more often require conditions that are difficult to scale up: for example, reactions that require >100 psi hydrogen, anhydrous insoluble bases, insoluble reductants in reductive cross-coupling, multicomponent metallophotoredox protocols or precise water stoichiometry. Finally, since non-PGM-catalyzed reactions are more recent and emerging technologies, they are also more likely to be covered by IP (both composition of matter and use patents) than more established PGM-catalyzed reactions.

Historically, the hesitance to adopt emerging non-PGM catalysis in industry may have stemmed from another interesting reason. Several high-profile cases of reactions that were initially claimed as “non-metal-” or “non-PGM-catalyzed” processes were later proven to be catalyzed by ppm levels of precious metal contaminants in the catalyst, ligand, or other components of the reaction mixture.^16^ These examples may have led to unfair levels of skepticism regarding non-PGM catalysis in pharma, though anecdotal evidence suggests that this viewpoint has subsided.

With these challenges in mind, we see increasing evidence that pharmaceutical chemists are pursuing non-PGM catalysis—likely because of large potential economic advantages of non-PGM metal catalysts in comparison to their precious metal analogues. Scientists at Abbvie, Pfizer, and Boehringer Ingelheim have initiated a series of reviews highlighting the progress of nonprecious-metal-catalyzed transformations, with the first article published in 2019.^17^ These companies also participate in a precompetitive research alliance on non-PGM catalysis, which has resulted in the development of new methods. Iron complexes have been successfully field-tested as highly effective catalysts in practical, kilogram-scale industrial synthesis of pharmaceuticals.^18^

Meanwhile, some truly novel reactions specific to non-PGMs have been developed in the past decade. One key example is the efficient nickel-catalyzed cross-coupling of C–O and C–N bonds of phenol, amide, and ester electrophiles developed by Garg and co-workers.^19^ Despite the use of relatively cheap phenol derivatives as starting materials, these Ni-catalyzed cross-couplings have not yet found widespread, large-scale use in industry. One possible reason for this is that there are patents covering this type of reaction, which can be a deterrent when developing a potential manufacturing process.^20^ Nonetheless, it is worth emphasizing that non-PGM catalysis has the potential to enable entirely novel disconnections that cannot be realized using PGM catalysis and provide access to significantly shorter synthesis routes.

Before discussing specific applications of non-PGM catalysis, however, we first discuss some common perceived advantages and disadvantages from an industrial perspective. A couple of noteworthy perspectives on the topic of base metal or non-PGM catalysis have been published recently.^21^

### 1.1 Are Non-PGMs More Sustainable than PGMs?

**Figure 4:**
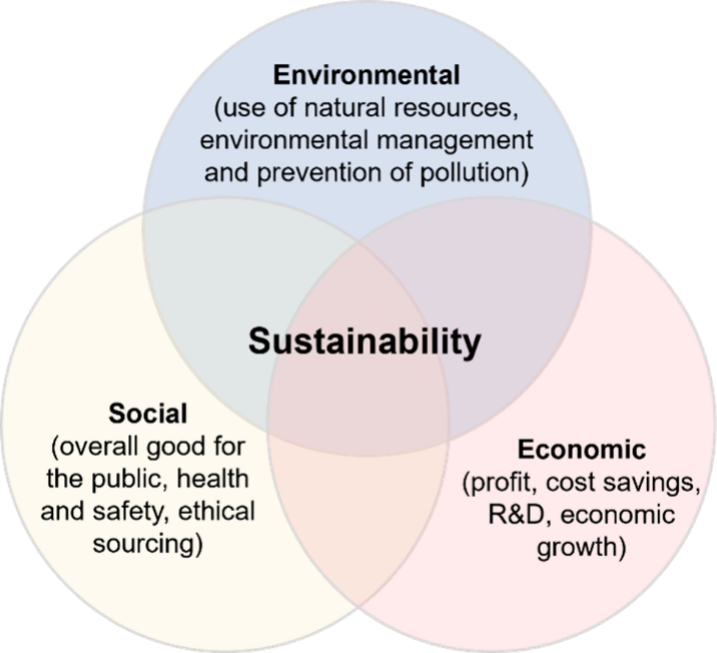
“Three circles” model of sustainability.

There is an increasing focus on sustainable manufacturing within the pharmaceutical industry.^22^ A result is greater attention to the origin and life cycle of all raw materials, including metal catalysts. Often, the word “sustainable” is used as shorthand for “environmentally friendly”.^23^ However, for a process to qualify as sustainable, other criteria need to be fulfilled, as well. As shown in Figure 4, the “three circles” model of sustainability nicely illustrates this.

It is noteworthy that catalysis in general is accepted as one of the ’12 principles of Green Chemistry’, as originally proposed by Paul Anastas and John Warner.^32^

Going into more detail, the fairest way to compare the sustainability of PGMs versus non-PGMs would be to assume that a particular process is using identical conditions and that the only difference is the use of, for example, palladium in one process and nickel in the other. This is unlikely, however, to be the case in the real world since the optimal reaction conditions (solvent, base, temperature, etc.) may not be the same for both metals.

A recent perspective by Drover and co-workers discusses the topic of sustainability of precious metals versus base metals.^33^ The authors proposed that the definition of a base metal also includes the origin and ethical mining of the metal, in addition to its natural abundance.

While a full analysis of the supply chain and lifecycle for each of the metals is outside the scope of the present review, we highlight a few key sustainability metrics in Table 1. In terms of global warming potential, the differences can be stark: production of PGMs requires 1,000–10,000 more CO_2_ equivalents than non-PGMs.^24^

**Table 1:** Sustainability Data for Selected PGMs and Non-PGMs

Entry	Element	CO_2_ emission (kg CO_2_ eq per kg metal)^24^	Crustal abundance (ppm)^25^	Annual production (tonnes)	Top 3 producers^26^
1	Pd	3,880	0.015	210^27^	1. South Africa
					2. Russia
					3. Zimbabwe
2	Ir	8,860	0.001	7^28^	1. South Africa
					2. Russia
					3. Zimbabwe
3	Rh	35,800	0.001	30^29^	1. South Africa
					2. Russia
					3. Zimbabwe
4	Ru	2,110	0.001	30^30^	1. South Africa
					2. Russia
					3. Zimbabwe
5	Ni	6.5	84	2,700,000^31^	1. Russia
					2. Indonesia
					3. Philippines
6	Cu	2.8	60	21,000,000^31^	1. Chile
					2. Peru
					3. China
7	Co	8.3	25	170,000^31^	1. DRC
					2. China
					3. Zambia

The disposal of metal-contaminated waste from a pharmaceutical process is part of the metal’s life cycle and thus a component of the sustainability question. Waste streams containing PGMs are typically combined for recovery by a specialized company, while non-PGMs are not.^34^ Recovery rates for heterogeneous catalysts are ∼90%, while those from solution are lower. Both PGMs and non-PGMs may need to be purged from aqueous waste streams due to their aquatic toxicity. When compared in isolation, PGMs may appear advantageous at this stage due to the common use of a recovery step, which is motivated by the cost of the metal but also provides waste remediation. Waste remediation for a non-PGM would thus be an added expense, though the overall process may still represent a considerable sustainability advantage.^35^

### 1.2 The Toxicity of PGMs vs Non-PGMs

It is worth briefly discussing the topic of “toxicity” relating to non-PGM catalysts since this has historically been an argument used in favor of certain elements (primarily iron and copper). We first refer the reader to a series of perspectives by Egorova and Ananikov; upon reviewing extensive toxicology data, the authors conclude that “*there are no catalytically demanded metals for which comprehensive toxic “portraits” could be built*”.^36,37^ The authors therefore argue against using toxicity as a selling point for new catalysts.

From the perspective of pharmaceutical manufacturing, there are two main areas of consideration when assessing metal catalyst toxicity: occupational safety and residual metal level in the final product.

The permitted level of residual metal impurities depends on the way the drug is administered; orally, by injection (parenteral), or by inhalation. Generally, the permitted levels of catalytically relevant non-PGMs are higher than those of PGMs (except for cobalt).^38^ Palladium, iridium, rhodium, and ruthenium all have the same permitted levels for oral drugs (100 μg/day), parenteral drugs (10 μg/day), and drugs administered by inhalation (1 μg/day) (Table 2, Entries 1–4). Nickel and copper are both permitted to a somewhat higher level; 200 and 2000 μg/day, respectively, for oral drugs, 20 and 300 μg/day, respectively, for drugs that are injected and 5 and 30 μg/day, respectively, for drugs that are administered by inhalation (Entries 5 and 6). Cobalt is more controlled, with permitted levels of 50 μg/day for oral drugs, 5 μg/day for parenteral drugs and 3 μg/day for drugs that are inhaled (Entry 7). Iron does not have a specified PDE (permitted daily exposure) and is treated as an “ordinary” impurity in most scenarios.

**Table 2:** PDE for Elemental Impurities from ICH Q3D

Entry	Element	Oral PDE (μg/day)	Parenteral PDE (μg/day)	Inhalation PDE (μg/day)
1	Pd	100	10	1
2	Ir	100	10	1
3	Rh	100	10	1
4	Ru	100	10	1
5	Ni	200	20	5
6	Cu	3000	300	30
7	Co	50	5	3

Metals with lower PDEs can require more intensive processes for their removal. The ease and cost for removing PGMs or non-PGMs from reaction streams for APIs must be considered as part of the overall cost and sustainability for their application. While various methods for PGM removal have been developed, the removal processes of non-PGMs are less refined, although promising studies have been reported.^39^

The aspect of occupational safety of non-PGMs is less uniformly regulated. In the EU, there are increased regulations on the use of nickel catalysis from this perspective since all nickel compounds are restricted under REACH. Discussions are also ongoing regarding REACH regulations for cobalt and some of its inorganic compounds. This can result in a specific process being viable to run in a manufacturing plant in Asia or North America but not in Europe.

### 1.3 History of Non-PGM in Industrial Processes

Non-PGM catalysis has played an integral part in the synthesis of fine chemicals for many years. Both homogeneous and heterogeneous catalysts derived from non-PGMs have been employed in several extremely important processes in the chemical industry, as exemplified by the Haber–Bosch process (heterogeneous iron), the production of syngas (heterogeneous nickel), the Dupont adiponitrile process (homogeneous nickel), and the Shell Higher Olefin Process (homogeneous nickel). This list is not exhaustive but nevertheless demonstrates the impact of non-PGMs to produce feedstock and fine chemicals on a very large scale.

#### Haber Process

**Figure 5:**
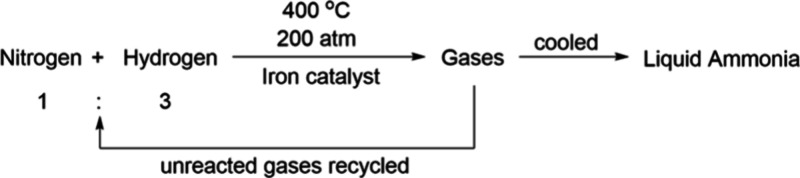
Haber–Bosch process.

The famous Haber process (also known as Haber–Bosch process) is the reaction of nitrogen and hydrogen under high temperature and pressure, over a heterogeneous iron catalyst, to produce ammonia (Figure 5). It was not until the start of the twentieth century that this method was developed to harness the atmospheric abundance of nitrogen to produce ammonia, which can then be oxidized to make the nitrates and nitrites essential to produce nitrate fertilizer and munitions.^40^ Today, over 450 million tons of nitrate fertilizer are produced annually, and it is estimated that 50% of all food grown on the planet is aided by this fertilizer.^41^

#### Syngas Synthesis

**Figure 6:**
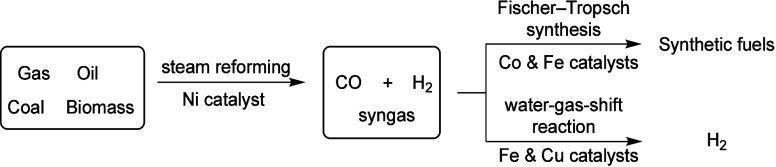
Both synthesis and the downstream application of syngas rely on non-PGM catalysis.

Syngas is a fuel source that mainly consists of hydrogen and carbon monoxide. One of the most important applications of syngas is the usage of it as a feedstock to produce an impressive number of modern industrial chemicals.^42^ Notably the synthesis and downstream application of syngas are highly dependent on non-PGM catalysis (Figure 6). One typical process in syngas synthesis is termed steam reforming, which generates hydrogen-rich synthesis gas from light carbohydrates by means of nickel catalysis. Furthermore, the Fischer–Tropsch process that produces synthetic fuels relies on cobalt or iron catalysis. Meanwhile, as the main industrial process for production of hydrogen, the water gas shift reaction (WGSR) relies heavily upon iron- and copper-based catalysts.^43^

#### The DuPont Adiponitrile Process

**Figure 7:**
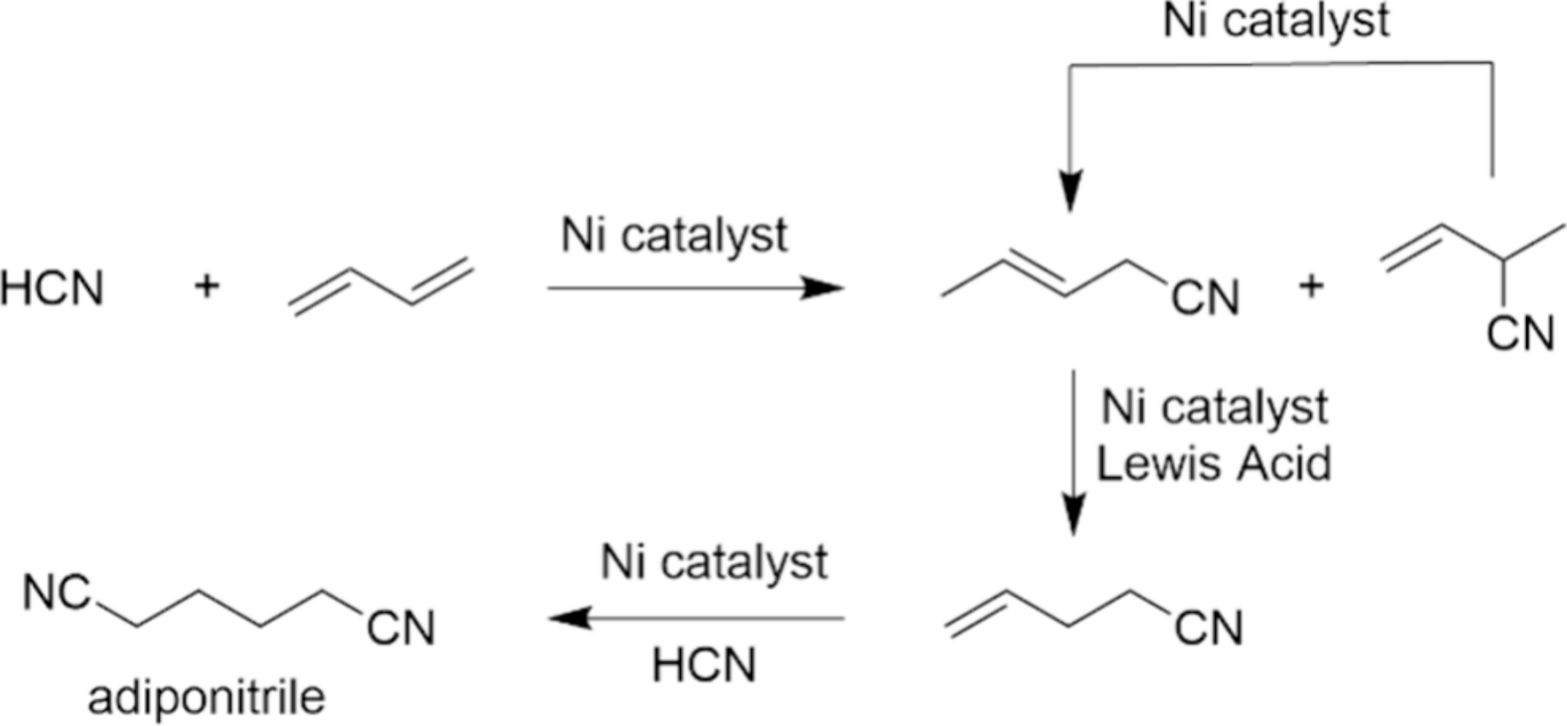
Dupont’s Ni-catalyzed hydrocyanation reaction.

A historically prominent nickel-catalyzed reaction is the olefin hydrocyanation reaction developed by DuPont to produce adiponitrile (Figure 7). This is an important precursor in the manufacture of nylon.^44^

The process utilizes nickel(0)–phosphite complexes, such as Ni[P(O-*o*-tol)_3_]_4_, as catalysts for the direct addition of HCN to butadiene, followed by a second anti-Markovnikov addition of HCN to produce adiponitrile. This is the main route by which DuPont manufactures adiponitrile in the US. Since 1977, this process is also run in France together by DuPont and Rhone-Poulenc, with an annual capacity of 100,000 tonnes.

On an interesting side note, one of the first applications of the bidentate phosphine ligands XantPhos and DPEPhos, which are now commonly used ligands in Pd-catalyzed cross-coupling reactions, was in the Ni-catalyzed hydrocyanation of alkenes.^45,46^

#### Shell Higher Olefin Process (SHOP)

**Figure 8:**
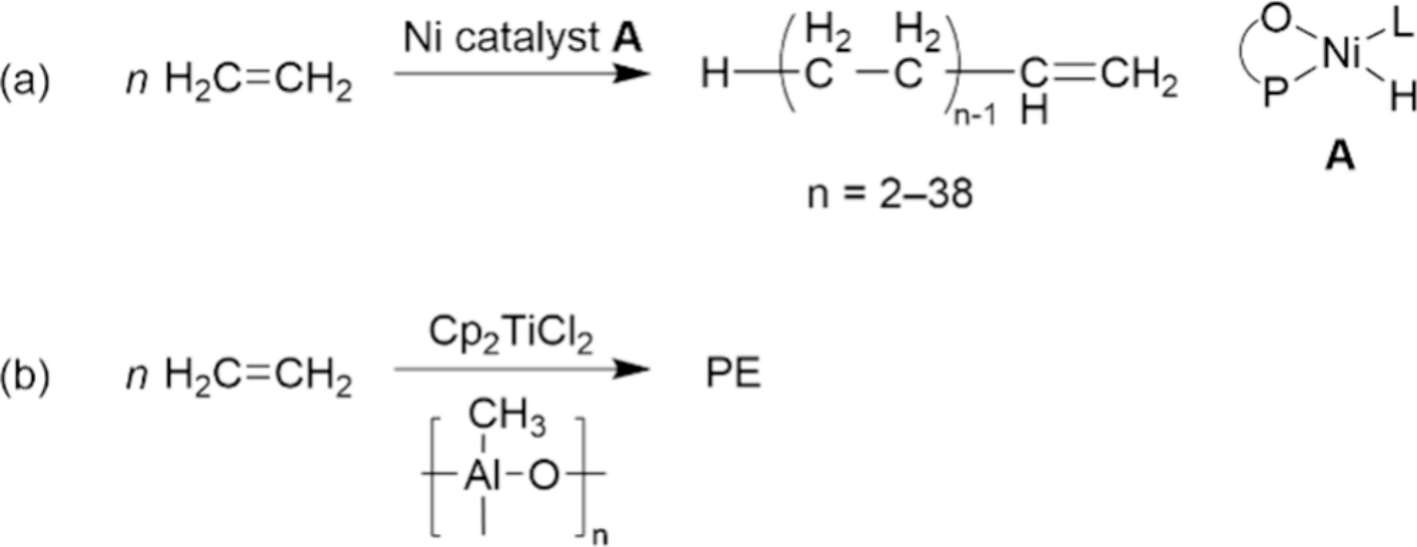
(a) Ni-catalyzed ethylene oligomerization to α-olefins; (b) metallocene-catalyzed polymerization.

Non-PGM catalysis has been widely applied in the petrochemical industry for several decades. Ziegler–Natta catalysts, a broad family that includes titanium complexes, have been employed to catalyze the polymerization of ethylene to produce polyethylene (PE). 90% of the high-density polyethylene (HDPE) in the world is produced using Ziegler–Natta catalysts. The synthesis of advanced polymers, e.g., linear low-density polyethylene (LLDPE), requires extensive use of homogeneous base metal catalysts. SHOP, that was discovered as early as 1968, was achieved by means of nickel-catalyzed oligomerization and metathesis of ethylene.^47^ As shown in Figure 8, the nickel–phosphine catalyst **A** is generated in situ from a nickel salt, NaBH_4_, a P,O-ligand, and ethylene.^48^ Currently, over one million tonnes of C12–C18 α-olefins are manufactured every year using SHOP.^42^ As demand for specialty polymers increases, metallocene catalysts are growing in significance.^49^ In 2009, a zirconium catalyst was put into operation in the 150,000 ton α-Sablin process in Saudi Arabia.^50^ Mitsui Petrochemical also developed a homogeneous catalytic process with titanium-based catalysts.^51^

#### Other Noteworthy Applications

In addition to the non-PGM catalysts mentioned above that have been used for a long time, vanadium has also been used as a chemical catalyst for sulfuric acid production for over 100 years.^52^ Aluminosilicates are a critical component of modern petrochemical manufacturing.^53^ Sometimes, in the case of heterogeneous catalysis, the nature of the support can have a significant impact on catalytic properties, and aluminum can be used for this purpose in the shape of alumina (Al_2_O_3_). Platinum supported on alumina are highly effective heterogeneous catalysts allowing for the dehydrogenation of alkanes.^54^ Olefin hydroformylation represents a large-volume application of catalysis.^55^ Originally, this process was achieved using cobalt catalysis; however, high pressure was required to drive these reactions forward. Now, rhodium-based catalysts are more commonly used, since lower pressure can then be applied.

In the fuel cell industry, there has been significant research in the replacement of the commonly used platinum-based electrocatalysts with non-PGM-based electrocatalysts (iron, cobalt, etc.). This would be a major step forward to produce hydrogen from water and renewable energy.^56^

## 2 NON-PGM CATALYSIS RELEVANT TO THE PHARMACEUTICAL INDUSTRY

As exemplified throughout the following sections of this paper, the introduction of non-PGM catalysis in other chemical sectors, including the pharmaceutical industry, is now becoming increasingly apparent. In this section, selected examples from the literature are discussed that illustrate emerging trends for the metals highlighted below. The selection of examples focuses on large-scale^57^ or especially pharmaceutically relevant applications of non-PGM-catalyzed reactions.

### 2.1 Nickel

The smaller size and decreased electronegativity of nickel relative to palladium enable much more facile oxidative addition to weak electrophiles. Beginning in the 1990s, reports emerged demonstrating that nickel catalysts were effective for cross-couplings of aryl methanesulfonate esters.^58^ More recently, several groups have developed nickel-catalyzed cross-coupling of aryl alkyl ethers, benzylic ethers, and certain amides. This advantage among others of nickel catalysis for cross-couplings has prompted researchers to consider its use for large-scale applications.

**Figure 9:**
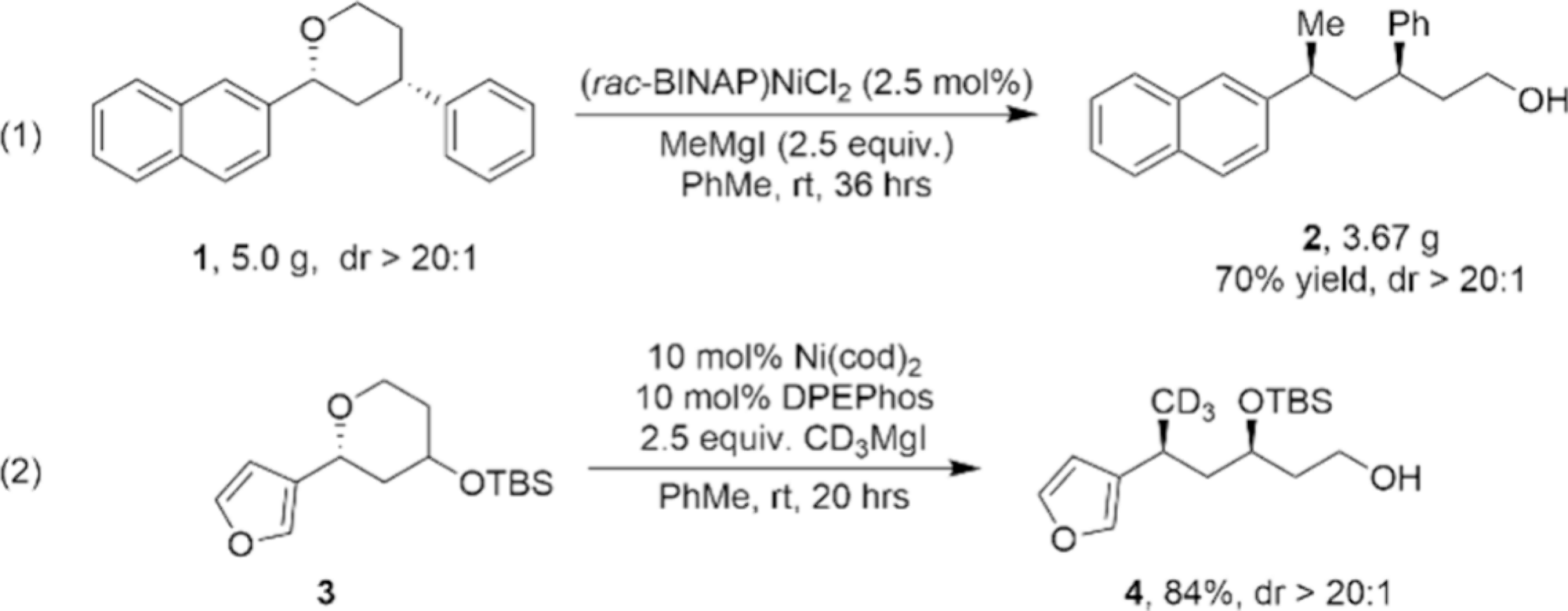
Nickel-catalyzed gram-scale Kumada cross-coupling reactions of benzylic ethers.

In 2015, Jarvo et al. reported a nickel-catalyzed gram-scale Kumada cross-coupling reaction of benzylic ether substrates (Figure 9, eq 1).^59^ Using bidentate nickel catalyst (*rac*-BINAP)NiCl_2_, the reactions proceed with inversion at the benzylic position, providing the corresponding ring-opened product **2** with high stereospecificity. As shown in Figure 9, eq 2, by changing the nickel catalyst to Ni(COD)_2_ (or Ni(dppe)Cl_2_), the methodology can be used to synthesize ring-opened products with isotopically labeled substituents, such as **4**. The reaction was demonstrated on multigram scale, with high yield and stereospecificity maintained. This reaction is expected to find use for the preparation of isotopologues of API intermediates, provided that their functional groups tolerate the presence of a Grignard reagent.

As with many other reports from academic settings, it is often only a matter of time before an industrial chemist identifies one of these methodologies as potentially useful for a target structure and sets to work to implement it. The other important function of publications like this is to provide a foundation for or inspire further developments within the field to identify other useful transformations. The example highlighted above represents a selected example from the advances in the field within the academic setting. There are numerous other reports^60^ that have been crucial to the overall advancement and understanding of the non-PGM catalysis area. Nevertheless, the focus of this review will lie on the development and implementation of this technology for larger scale purposes in industry.

**Scheme 1:**
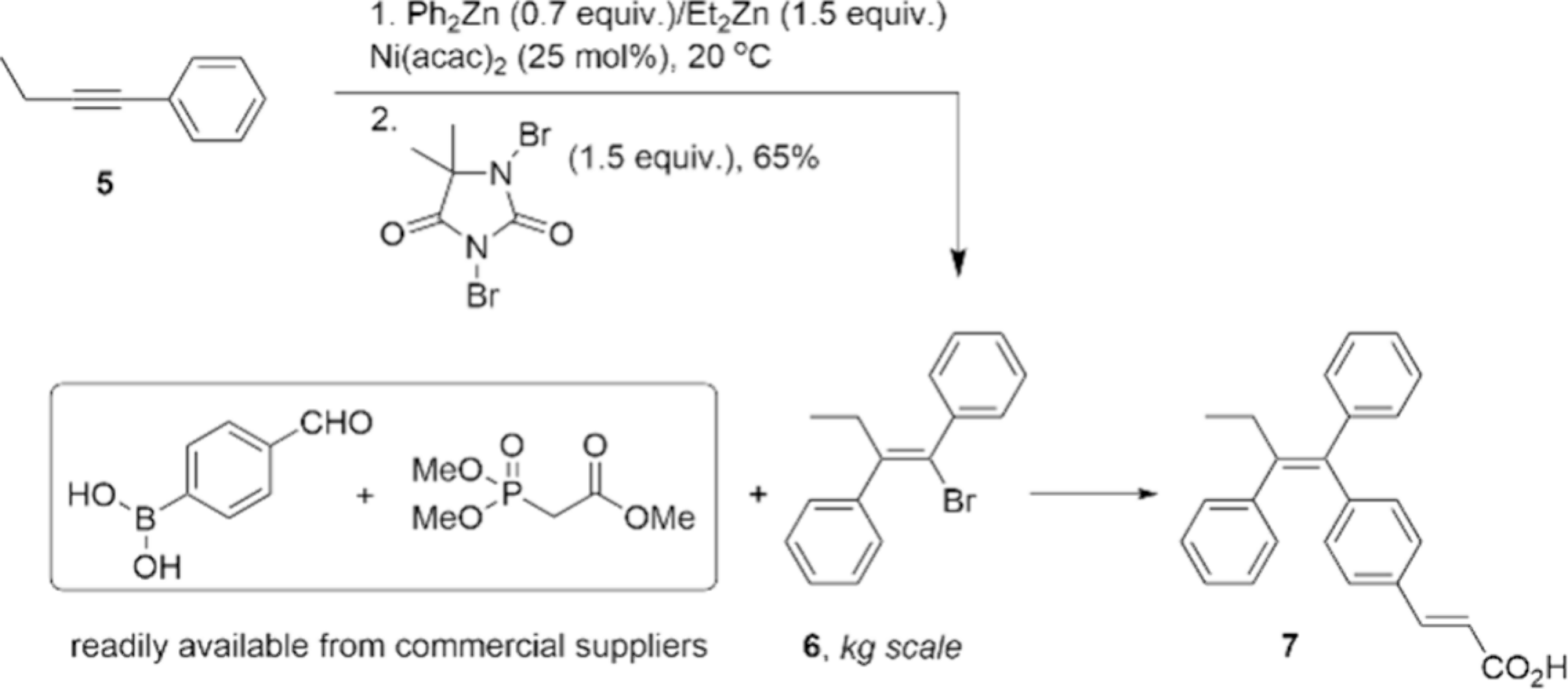
Kilogram-Scale Stereoselective Synthesis of 6

In 2010, scientists at BMS developed a process taking advantage of the catalyst Ni(acac)_2_ to produce multikilogram quantities of alkenyl bromide **6** (Scheme 1),^61^ a key intermediate for the synthesis of alkene derivative **7** that has attractive properties as estrogen agonist/antagonist which are desirable for the treatment of breast cancer. With a relatively high loading of the inexpensive Ni(acac)_2_ (25 mol %), carbometalation of but-1-ynylbenzene **5** with diphenylzinc/diethylzinc can proceed under moderate temperature, followed by bromination with *N*,*N’*-dibromo-5,5-dimethylhydantoin under well-defined conditions, yielding **6** in 65% isolated yield with high stereoselectivity (Scheme 1). After subsequent recrystallization, **6** can be obtained at 99.3% purity with only <0.1% diethyl analogue and none of the (*E*)-isomer detected. The ease by which the synthesis of **6** can be scaled-up, combined with the commercial availability of the other two synthons enlisted subsequently, (4-formylphenyl)boronic acid and methyl 2-(dimethoxyphosphoryl) acetate, ensured the production of enough **7** for pharmaceutical development activities and clinical trials.

**Scheme 2:**
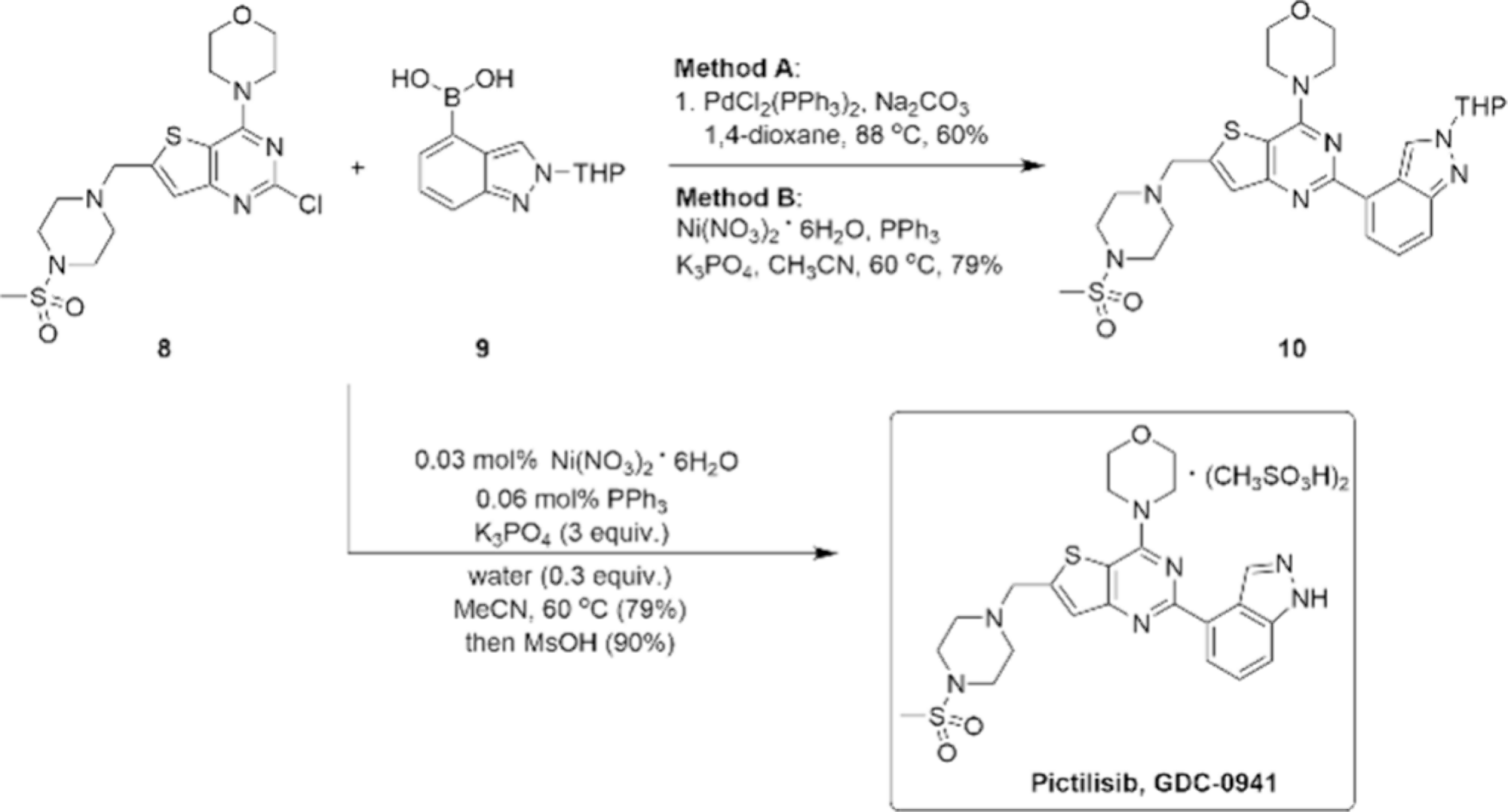
Two Methods Used for the Kg-Scale Synthesis of Key Intermediate 10 and the Synthesis of Pictilisib

Another example reflecting the economic benefits of nickel is the nickel-catalyzed Suzuki–Miyaura cross-coupling reaction to form **10**, a key intermediate to produce pictilisib, which was achieved on multikilogram-scale.^62^ As shown in Scheme 2, Pictilisib, also known as GDC-0941, is a novel small molecule PI3K inhibitor discovered by Genentech, and it was evaluated as an anticancer agent. The Suzuki–Miyaura reaction between substrates **8** and **9** was developed initially to produce substantial amounts of GDC-0941 that can be used for supporting further pharmaceutical development activities. The team developed both PdCl_2_(PPh_3_)_2_ and Ni(NO_3_)_2_·6H_2_O/PPh_3_ as efficient catalysts for this late-stage cross coupling.

The decision about which catalyst to use ultimately depends on the following factors: substrate performance and the cost of removing impurities. On both points, the route with the nickel precatalyst, which afforded the THP protected **10** in 79% yield, won out over the palladium catalyzed route (Scheme 2). The removal of the residual palladium contaminant required the use of relatively expensive scavengers (Florisil and Thio-Silica) and a large volume of solvents; in contrast, the residual nickel catalyst could be easily removed from the crude reaction mixture through an aqueous ammonia wash and crystallization. Moreover, the precatalyst Ni(NO_3_)_2_·6H_2_O is relatively cheap (ca. $15/mol) and readily available. Furthermore, the cross-coupling could be operated at an impressive 0.03 mol % catalyst loading.

**Figure 10:**
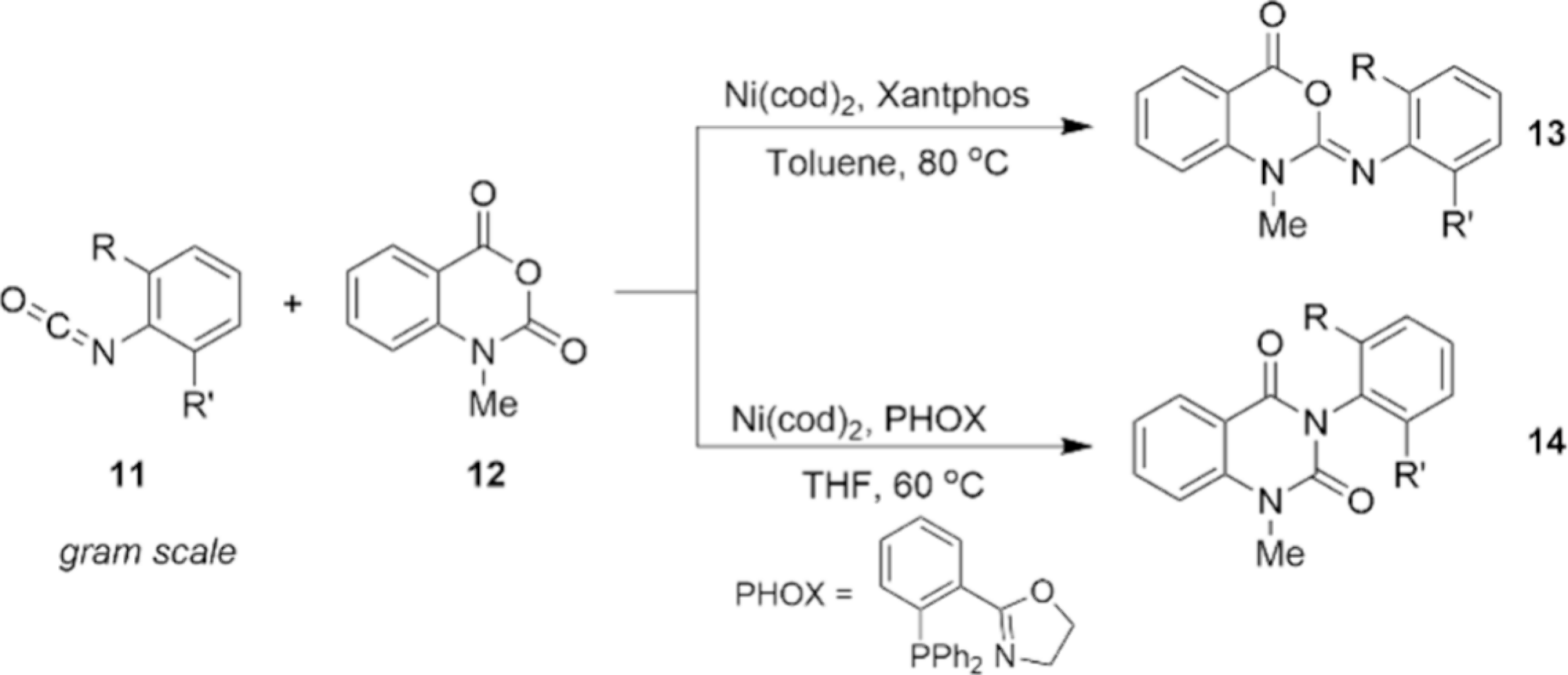
Switching ligands allowed access to either of the constitutionally isomeric products shown.

In precious-metal-catalyzed reactions, it is common that the identity of the ancillary ligands can influence product selectivity, and the same is true for non-PGM-catalyzed reactions. Taking the nickel(0)-catalyzed reaction of isocyanates **11** and isatoic anhydrides **12** as an example, screening reaction showed changes in the substitution pattern of the isocyanate led to different products.^63^ XantPhos was unique to forming the benzoxazinone imine **13**, while PHOX ligand allowed for synthesis of the constitutionally isomeric quinazolinediones **14** in excellent yields and selectivities (Figure 10).^64^ Both reactions can be performed at the gram scale. This example nicely illustrates that the development of general processes for base-metal catalysis also requires highly efficient catalyst and reaction screening systems, similar to those commonly used in PGM-catalyzed reaction development. The difference may be that there is currently less knowledge of which ligands may be effective in combination with non-PGM catalysts in a specific transformation. This can turn a non-PGM catalysis screening project into more of a “guess-work” and less of a knowledge-based project.

**Scheme 3:**
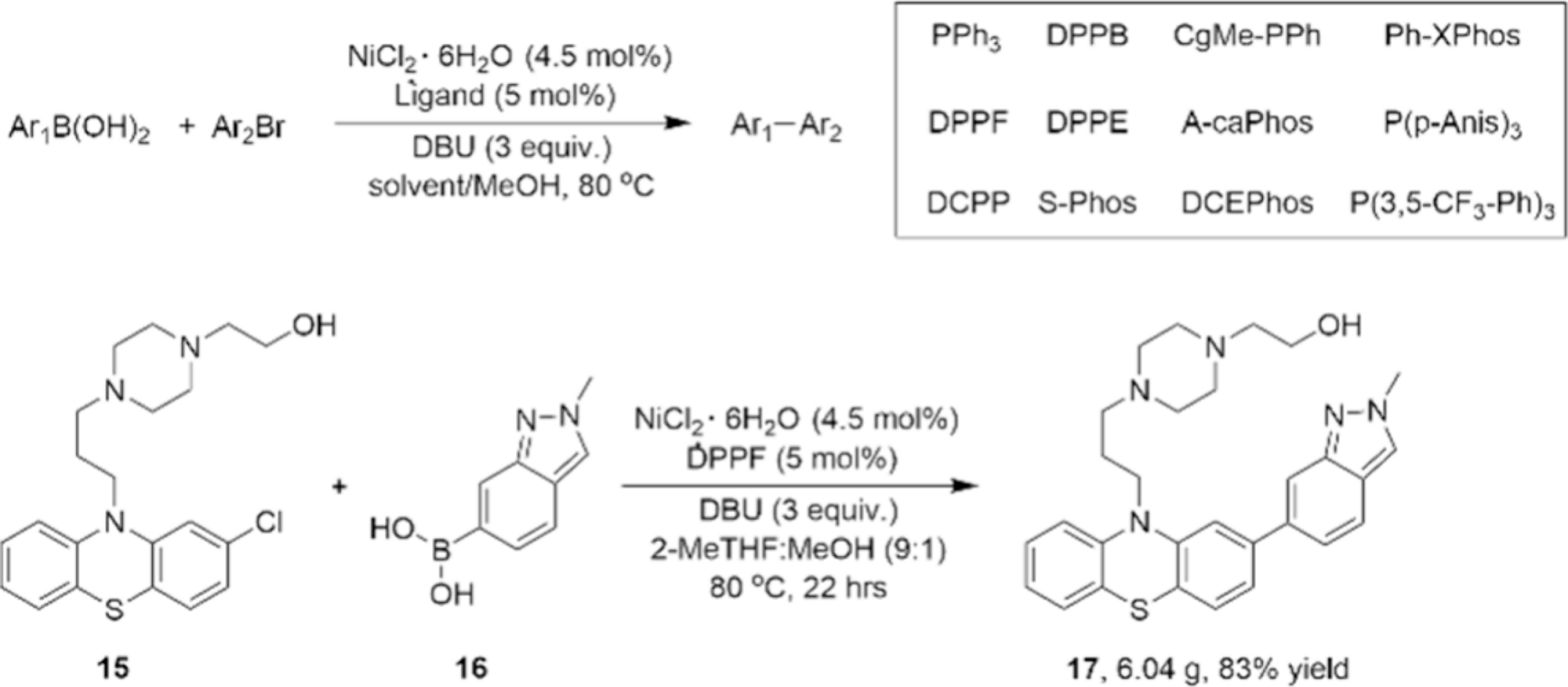
Screening of the Boronic Acids and Aryl Halides in Suzuki–Miyaura Coupling Reactions and Gram-Scale Synthesis of 17

Recognizing the value of testing a diverse collection of ligands during the initial evaluation of nickel catalysis in a potential transformation of interest, chemists at BMS reported the development of a 24-reaction screening platform for identifying nickel-catalyzed Suzuki–Miyaura reaction conditions.^65^ The screening platform includes NiCl_2_·6H_2_O as a catalyst and 12 monophosphine and bisphosphine ligands. The crucial part of this platform is the methanol additive that significantly improves the reaction performance and enables the use of organic-soluble amine bases (Scheme 3). The ligand screening platform enabled rapid ligand screening for API synthesis with nickel catalysis, as demonstrated by a gram-scale coupling reaction of antipsychotic perphenazine **15** with boronic acid **16** to form **17** in 83% yield. This methodology was designed to be directly applicable to process scale-up by achieving homogeneous reaction conditions employing stable and inexpensive nickel(II) precatalysts.

**Scheme 4:**
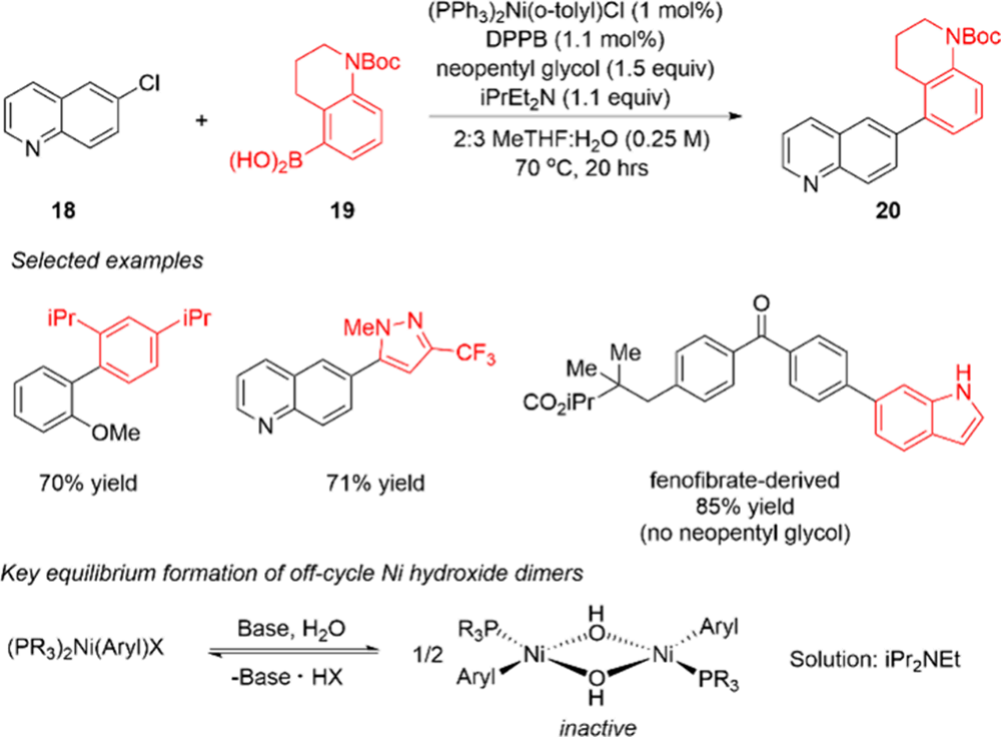
Ni-Catalyzed Suzuki–Miyaura Reaction of Heteroarylboroic Acids under Aqueous Conditions

**Scheme 5:**
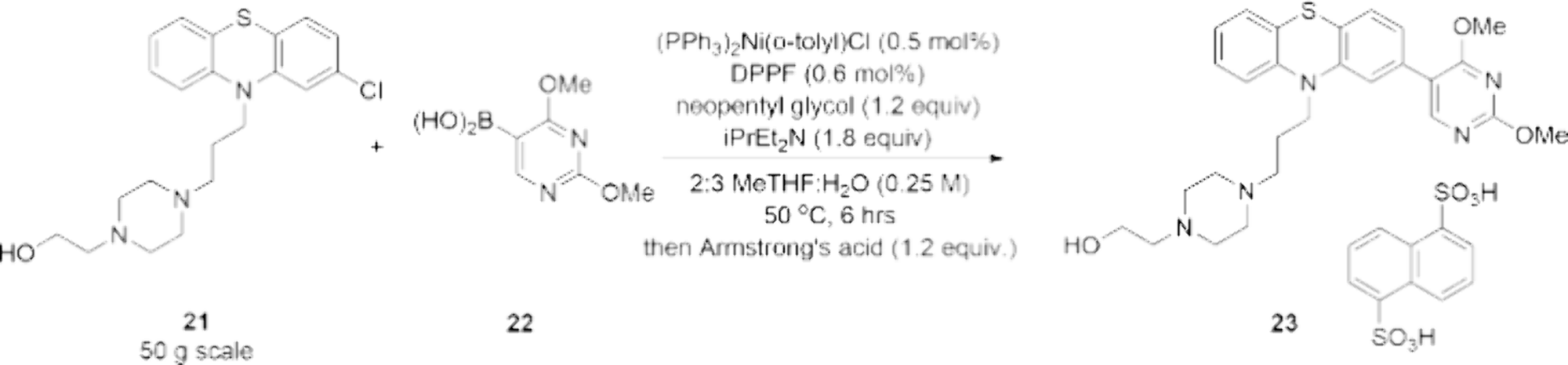
Perphenazine Cross-Coupling on a 50-g Scale

Following their demonstration of homogeneous conditions (MeOH and DBU) for nickel Suzuki–Miyaura reaction,^66^ BMS researchers sought to develop aqueous conditions that more closely mimicked those used in the palladium version. Previously, this was challenging for nickel due to the formation of inactive Ni-hydroxide species. As shown in Scheme 4, the authors found that amine bases allowed the nickel-hydroxide formation to be more reversible than that with traditional inorganic bases, and conditions were developed with *i*-Pr_2_NEt in 2-MeTHF/water. The reaction was catalyzed by a combination of the phosphine ligand and the versatile, air-stable nickel precursor, (PPh_3_)_2_Ni(*o*-tolyl)Cl. DPPB (1,4-bis(diphenylphosphino)butane) had the most generality as the ancillary ligand, though in some cases other common phosphines were optimal. The authors demonstrated an impressive substrate scope containing pharmaceutically derived or relevant heteroaryl-boronic acids and aryl chlorides. An example was demonstrated by a Suzuki–Miyaura cross-coupling reaction on 50 g of pherphenezine **21** using only 0.5 mol % Ni, and the product **23** was isolated by salt formation with only 10 ppm residual nickel (Scheme 5).

**Scheme 6:**
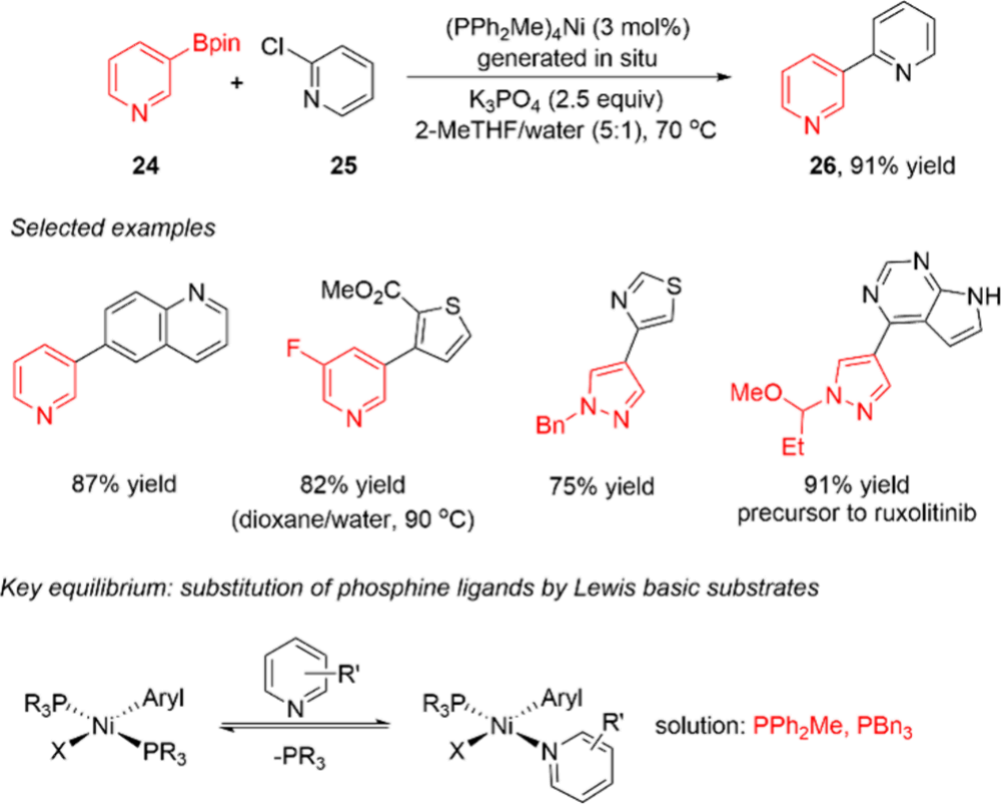
Ni-Catalyzed Small-Scale Suzuki–Miyaura Reaction of Lewis Basic Aryl-Bpin Substrate 24 under Aqueous Conditions

These two examples from the groups at BMS illustrate how demands posed by industrial applications led to the advancement of synthetic methodology—in both cases, addressing the need for easily scalable reaction conditions and applicability to decorated heterocyclic substrates. In a similar vein, a collaboration between scientists at AbbVie and Pfizer sought to address another major limitation of the Ni-catalyzed Suzuki–Miyaura reaction: poor reactivity with Lewis-basic arylboron nucleophiles.^67^ The authors found that the majority of widely studied phosphine/Ni catalysts were unable to promote the coupling of a (pyridyl)Bpin **24**, while the simple ligand PPh_2_Me was highly effective.^68^ The reactions, which were performed from milligram to gram scale, proceed under “standard” palladium-catalyzed Suzuki–Miyaura reaction conditions with K_3_PO_4_ as base in 2-MeTHF/water as the solvent (Scheme 6). Under these conditions, many Lewis basic (aryl)Bpin substrates could be coupled with functionalized heteroaryl halides in moderate to high yield. The active catalyst (PPh_2_Me)_4_Ni was generated by mixing (PPh_2_Me)_2_NiCl_2_, PPh_2_Me and *n*-BuMgCl, though (PPh_2_Me)_2_Ni(o-tolyl)Cl, PPh_2_Me and (TMEDA)Ni(*o*-tolyl)Cl were also effective catalysts. The surprising activity of PPh_2_Me was shown to result in part from the ability of this ligand to resist substitution by Lewis basic substrates at Ni(aryl)X intermediates.

**Scheme 7:**
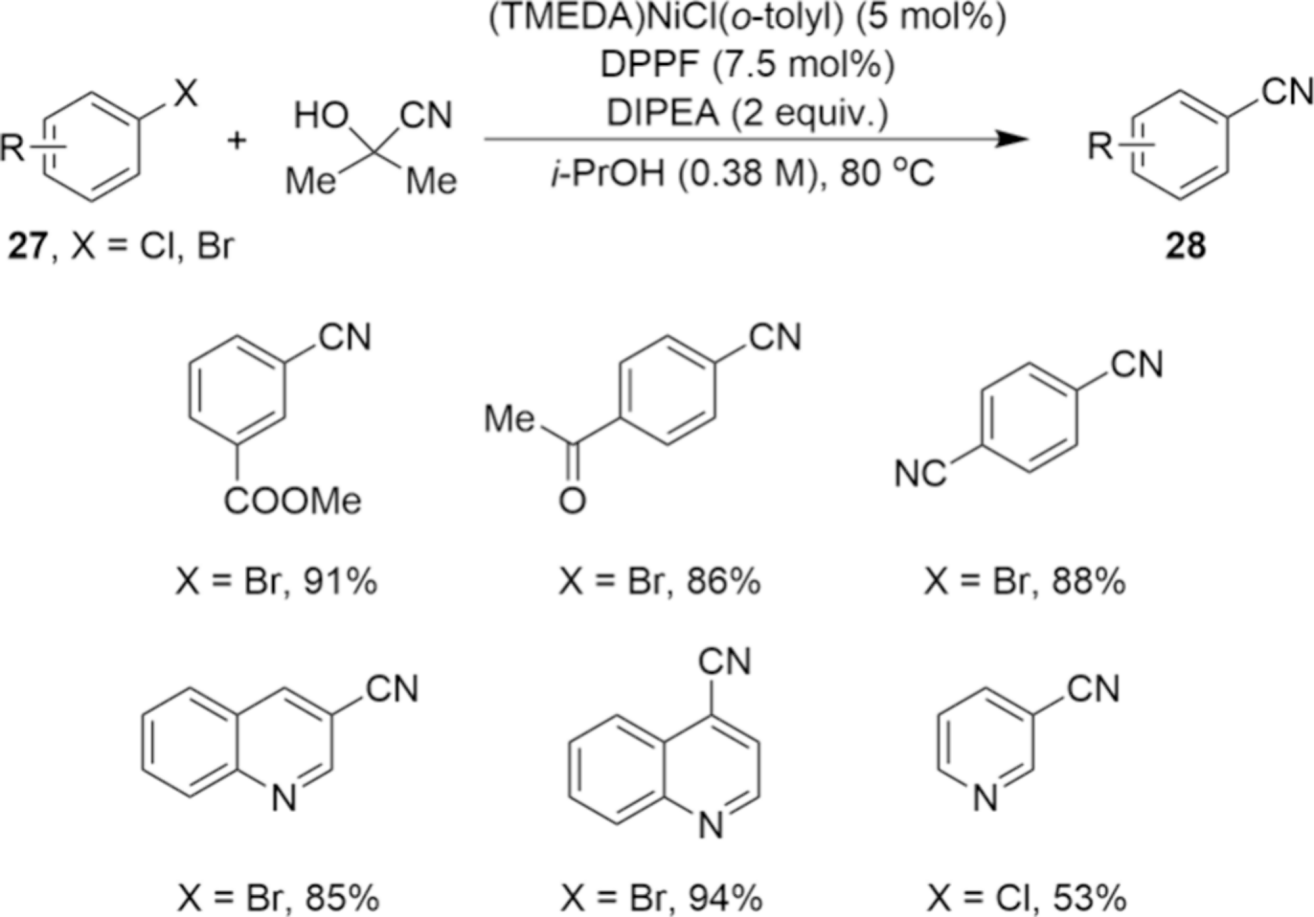
Milligram-Scale Synthesis of Simple Benzonitriles 28 by Nickel-Catalyzed Cyanation of Aryl Halides

Nickel catalysis has also found industrial relevance in other cases involving challenging nucleophiles. In 2016, scientists at Bayer reported a mg scale nickel-catalyzed cyanation of aryl halides **27**.^69^ As shown in Scheme 7, the reaction uses the recently developed (TMEDA)NiCl(*o*-tolyl) as the precatalyst and bidentate phosphine dppf as the ligand. These reactions were run in 2-propanol as the solvent using DIPEA as a base at 80 °C, leading to reaction completion typically within 2 h, providing simple benzonitriles **28** in moderate to excellent yield. Interestingly, the reaction conditions are analogous to those of palladium-catalyzed reaction systems. Most simple electron-deficient aryl bromides are well-tolerated, while aryl chlorides and electron-rich aryl bromides gave low to no conversion. The authors pointed out that although the scope of nickel-catalyzed cyanation demonstrated to date is not as extensive compared to that of palladium-based methods, the operational simplicity coupled with the low cost of nickel will facilitate the development of this method to produce simple aryl nitriles on an industrial scale. An additional advantage is the use of acetone cyanohydrin (ACH) as the cyanide source, which is safer to handle than for example HCN

**Scheme 8:**
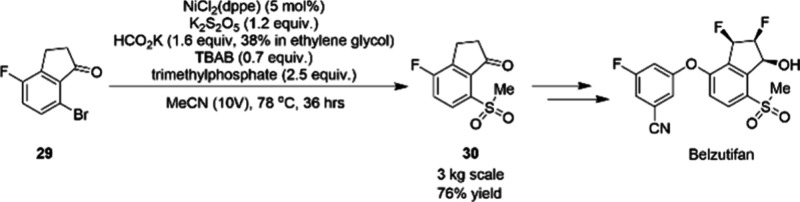
Ni-Catalyzed Sulfonylation Reaction of Aryl Bromide 29 to Intermediate 30 in the Manufacturing Process of API Belzutifan

In 2024, researchers at Merck described their efforts in developing a robust, and scalable nickel-catalyzed sulfonylation of **29** to access intermediate **30** as a means to identify a more sustainable and cost-effective manufacturing process of the API Belzutifan (Scheme 8).^70^ The previous process had utilized copper catalysis and sodium methanesulfinate for the sulfonylation, but this reagent was costly for the overall synthesis. The inorganic sulfur salt, potassium metabisulfite, was chosen as an inexpensive source of SO_2_, however, the reagent introduced stirring challenges due to heterogeneity of the reaction. Slow addition of the reductant, potassium formate, in ethylene glycol mitigated these mixing challenges, which ultimately allowed for the lowering of the amount of metabisulfite. This prevented catalyst poisoning with SO_2_, thus lowering the catalyst loading to 5 mol % NiCl_2_(dppe). Furthermore, the versatile nickel catalyst system was amenable to in situ methylation conditions of the aryl sulfinate using the nontoxic reagent trimethylphosphate, thereby circumventing the need for subsequent methylation. The process was demonstrated on 3 kg scale, providing 76% yield of the desired product **30**.

**Scheme 9:**
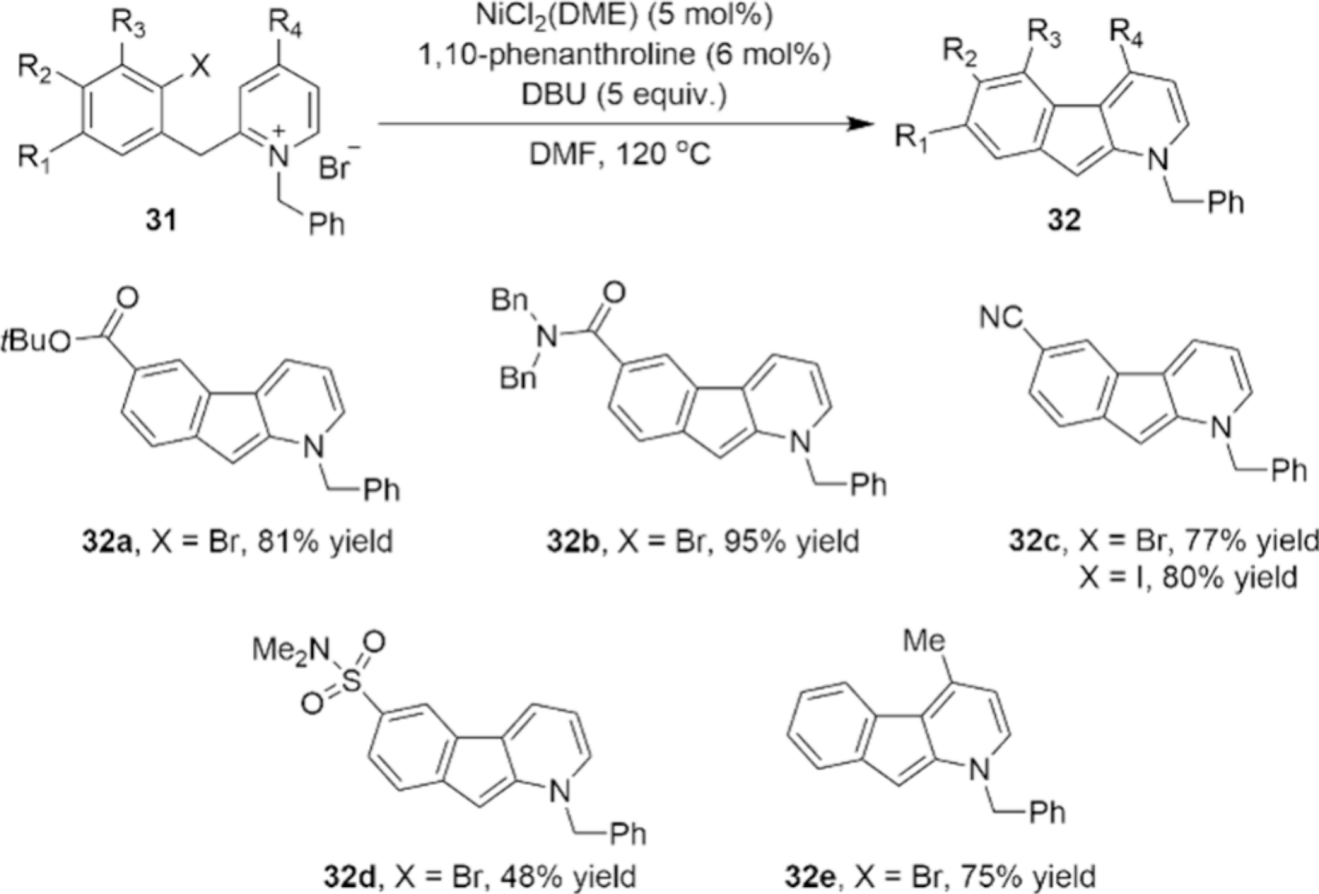
Nickel-Catalyzed C-3 Direct Arylation of Pyridine Derivatives 31 at Milligram Scale

**Scheme 10:**
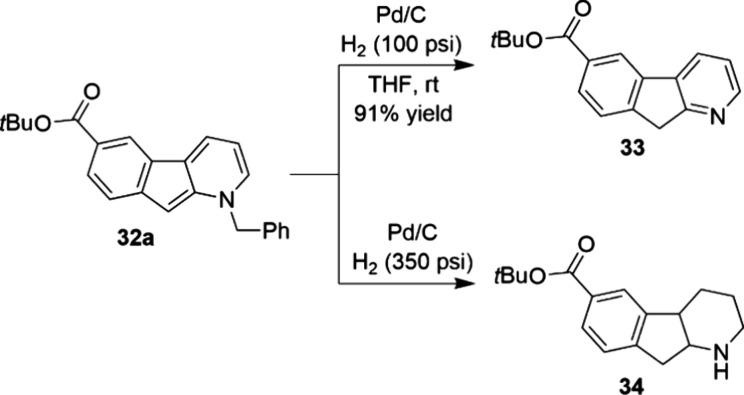
Debenzylation and Reduction of 1-Azafluorenes 32a to Valuable Pharmacophores 33 and 34

The combination of air-stable nickel catalyst precursors and phenanthroline ligands also enables the direct arylation of pyridine substrates using non-PGM catalysts. Chemists at Boehringer Ingelheim reported the first mg scale nickel-catalyzed C-3 direct arylation of pyridinium ion substrates (**31**), which was found to enable access to azafluorene pharmacophores (Scheme 9).^71^ This methodology, combining commercially available NiCl_2_(DME) as catalyst and inexpensive 1,10-phenanthroline as ligand, is compatible with a variety of substituents on either the aryl halide or pyridine core. As shown in Scheme 7, substrates with either electron-withdrawing substituents at C-6, such as esters **32a**, amides **32b**, nitriles **32c** and sulfonamides **32d**, or substituents at C-4 of the pyridine ring **32e** were well tolerated (77–95%). After debenzylation and reduction, the *N*-benzyl protected 1-azafluorenes can afford valuable pharmacophores, such as **33** and **34** (Scheme 10). Notably, the debenzylation step still requires the use of a palladium catalyst, highlighting the remaining PGM dependence of this synthesis route.

**Scheme 11:**
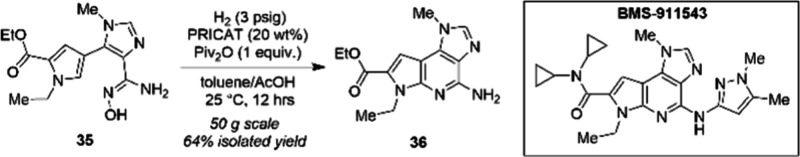
Nickel-Catalyzed C–H Functionalization in the Synthesis of BMS-911543 at Gram Scale

Another reported example of nickel-catalysis utilized to selectively construct cyclized structures is in the formation of the nitrogen-rich heterocycle core of the JAK2 inhibitor BMS-911543.^72^ The scientists at BMS found that under low pressure hydrogen using Ni(acac)_2_(H_2_O)_2_ with nickel oxide supported on silica (PRICAT), the C–N cyclization of pyrrole and activated hydroxylamine in compound **35** was observed in 77% yield and 16:1 selectivity for the desired cyclized product **36** (Scheme 11). This method provided a fruitful route for the heterocyclic core, which had been unsuccessful to construct via 6π-electrocyclization pathways. Notably, no product was obtained if unsupported Ni(acac)_2_(H_2_O)_2_.

**Scheme 12:**
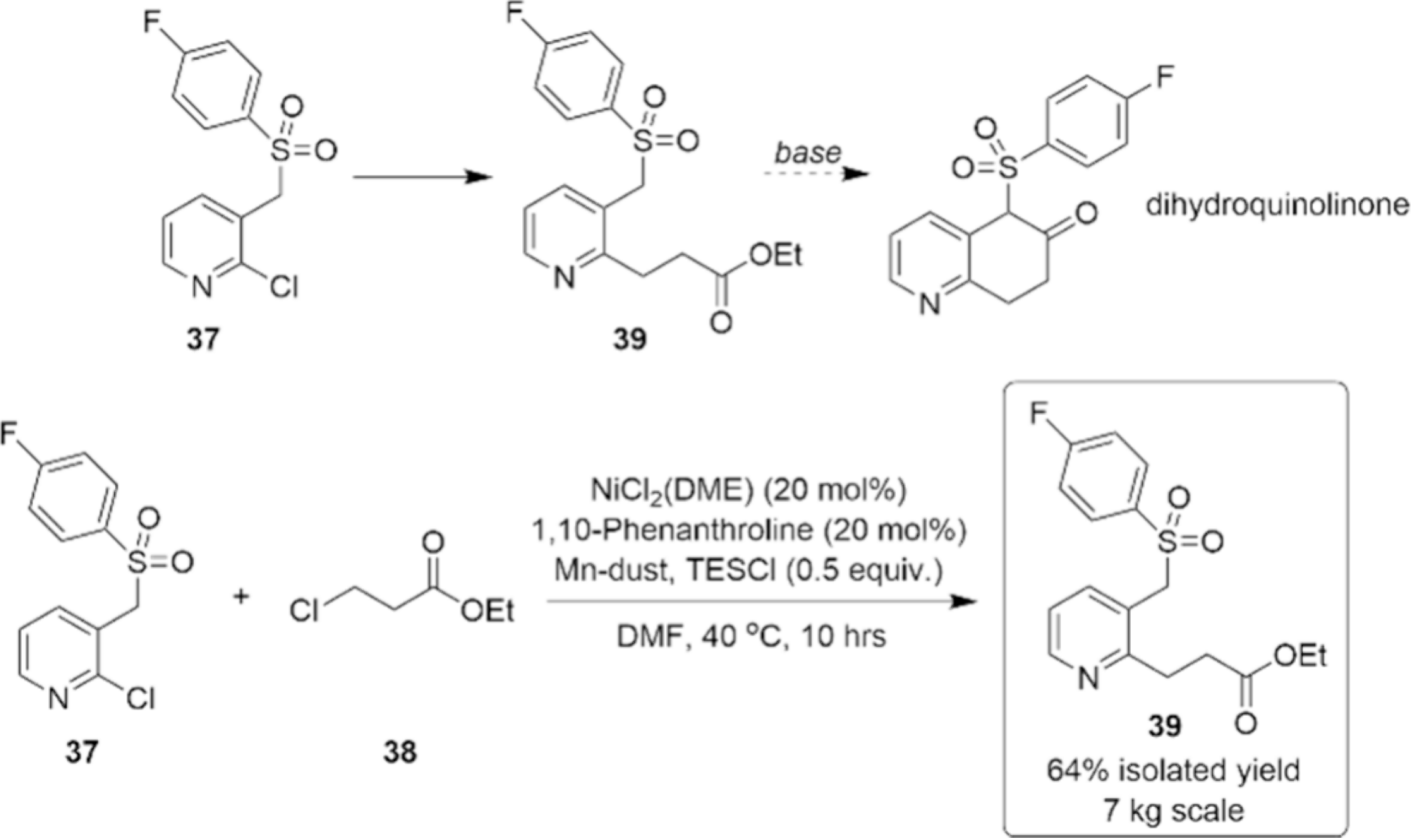
Kilogram-Scale Nickel-Catalyzed Reductive Cross-Coupling Reaction Yielding 39

In 2020, researchers at BMS described a practical method taking advantage of a nickel-catalyzed reductive cross-coupling strategy for the kg scale synthesis of **39**, a key intermediate toward dihydroquinolinone core structures (Scheme 12).^73^ Initial experiments showed that the Pd-catalyzed Negishi cross-coupling afforded the desired product in 65% conversion; however, the limited availability of the organozinc species on a large scale proved to be a constraint. Suzuki- and Heck-based strategies also failed. Fortunately, catalyst and ligand screening revealed that the combination of NiCl_2_(DME) and 1,10-phenanthroline was the best catalytic system for the reductive coupling of 2-chloropyridine **37** and ethyl 3-chloropropanoate **38**. In addition, stoichiometric manganese as the terminal reductant and chlorotriethylsilane (TESCl) as the activator provided optimal conversion. The process was demonstrated on a 7 kg scale and afforded **39** in 64% yield. Obviously, a comprehensive understanding of mixing requirements, including maintaining an optimal suspension of manganese to promote catalytic turnover and identifying the appropriate agitation parameters through modeling, was important for the successful scale-up of this reaction.

It is worth mentioning that newly revived synthesis methodology, such as photocatalysis and electrocatalysis, has also relied on the application of non-PGM catalysts.^74^

**Scheme 13:**
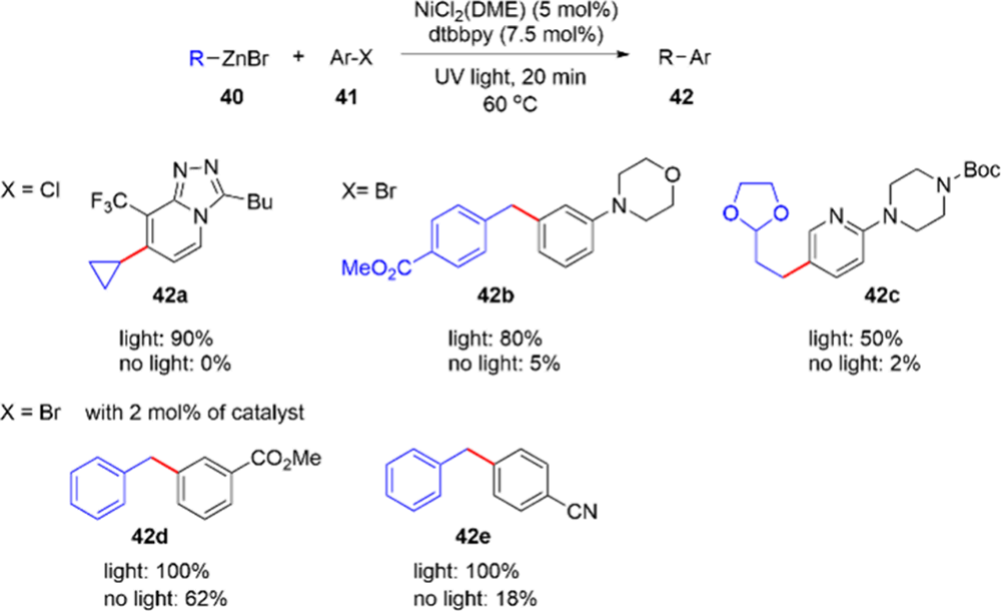
Nickel-Catalyzed Negishi Cross-Couplings at Milligram Scale

Alcázar and co-workers reported a mg scale nickel-catalyzed Negishi cross-coupling between freshly prepared organozinc derivative **40** and aryl halides **41**.^75^ As shown in Scheme 13, the reaction efficiency can be accelerated extensively by visible-light irradiation alone without the addition of any photocatalysts. The reaction was performed by using a NiCl_2_(DME) and dtbbpy catalyst system. For the synthesis of **42a**, the yield of the product differs by as much as 90% with and without light participation. Particularly, light irradiation had a significant effect on the conversion when strongly electron donating groups were present in the molecule, such as in compounds **42b** and **42c**. Notably, as represented by **42d** and **42e**, substrates with electron-withdrawing groups only required 2 mol % of the nickel catalyst. Moreover, as the reaction is carried out in flow, direct scalability could be relatively easily achieved and the overall approach is superior to batch protocols.

**Scheme 14:**
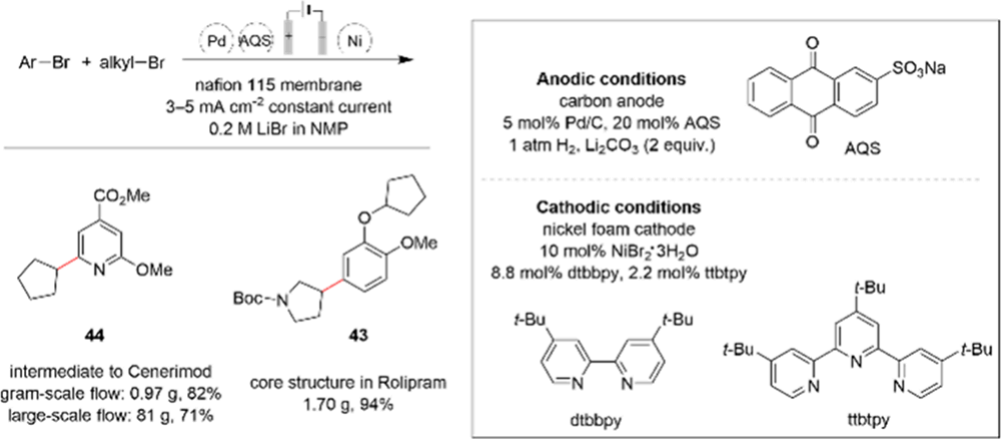
A Scalable Electroreductive Nickel-Catalyzed Cross-Electrophile Coupling of Aryl and Alkyl Bromides

With respect to nickel catalysis combined with electrochemical technique for the purpose of cross-electrophile coupling reactions; a nice review was published recently by Prof. Qiu et al.^76^ Nickel-catalyzed reductive couplings for C(sp^2^)–C(sp^3^) bond formation are becoming well-developed methodologies. Nevertheless, performing these reactions on an industrial scale introduces a number of challenges due to the sacrificial stoichiometric metal or amine reductants typically employed. In 2023, a scalable electrochemical approach was demonstrated for the synthesis of pharmaceutically relevant structures. The method utilizes H_2_ as the formal reductant in an electrochemical setup by leveraging a cascade of catalytic reactions.^77^ The nickel-catalyzed coupling was optimized to 10 mol % NiBr_2_ trihydrate and a 4:1 ratio of the ligands dtbbpy and ttbtpy. The method was performed on a gram scale using an electrochemical flow reactor to obtain core structure **43** found in Rolipram and intermediate **44** in the synthesis of Cenerimod (Scheme 14).

**Scheme 15:**
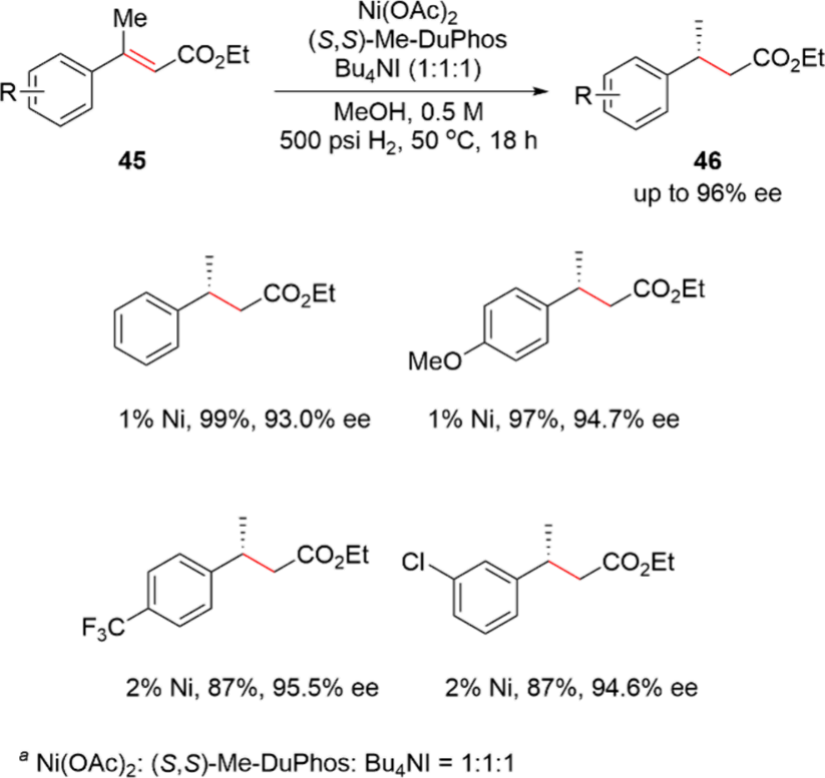
Milligram-Scale Asymmetric Hydrogenation of 45 with a Combination of Nickel Catalyst and Me-DuPhos Ni(OAc)_2_: (*S,S*)-Me-DuPhos: Bu_4_NI = 1:1:1

In recent years, the advanced bidentate phosphine ligands from the DuPhos and BPE families have also demonstrated utility in non-PGM catalyzed asymmetric hydrogenation reactions. In particular, the Me-DuPhos series has been extensively investigated for non-PGM catalysis in academia during the past decade. As shown in Scheme 15, taking advantage of the coordination chemistry of (*S*,*S*)-Me-DuPhos, Chirik and co-workers developed a highly active and enantioselective phosphine–nickel catalyst for the asymmetric hydrogenation of α,β-unsaturated esters **45**.^78^ Reaction screening demonstrated that an air-stable nickel source in combination with a Bu_4_NI additive as well as Me-DuPhos with a molar ratio of 1:1:1 was the most effective catalytic system in methanol. The reaction was performed at milligram scale and tolerated a range of substrates with electron donating and withdrawing functional groups, yielding (*S*)-**46** with up to 99% isolated yield and 96% ee. With the increasing commercial availability of the DuPhos and BPE family of ligands, this is now becoming a methodology worthy of attention for the process chemist.

### 2.2 Copper

Copper catalysis is probably the most widely applied alternative to PGM catalysts in the pharmaceutical setting. The foundations of modern copper-catalyzed coupling reactions were established from Ullmann and Goldberg’s pioneering research.^79^ Since then, numerous copper-catalyzed cross-coupling reactions, especially those conducted under ambient conditions, have been developed in academia to construct C–C and C–heteroatom bonds. Importantly, the discovery of advanced bidentate ligands, such as diamines, 1,3-diketones and oxalic diamides, over the past two decades has extensively expanded the substrate scope of copper catalysis. Ma and co-workers have comprehensively summarized the development of useful reaction conditions for the coupling of (hetero)aryl halides with different nucleophiles.^80^ Copper-mediated functionalization of aryl halides was well reviewed by Priyadarshini et al.^81^ Furthermore, pyridines and pyrimidines produced via copper catalysis,^82^ as well as the market potential and prospects were also systematically reviewed.^83^

The Chan–Lam coupling, the copper-catalyzed C–N or C–O oxidative coupling of organoboron and heteroatom nucleophiles, is an excellent example of a transformation that is well-accepted among both medicinal and process chemists.

**Scheme 16:**
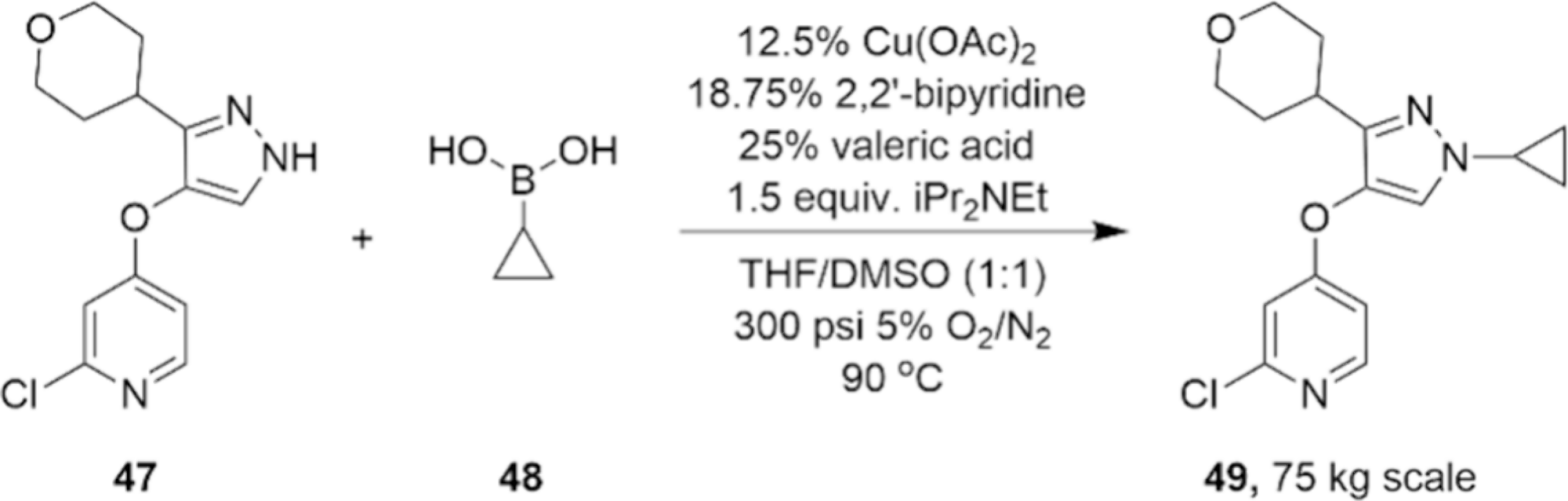
Kilogram-Scale Preparation of 49 via Optimized Cu-Catalyzed Chan–Lam Coupling Reaction

Taking advantage of aerobic oxidation, in 2019, scientists at Lilly reported a copper-catalyzed continuous process for multikilogram scale production of penultimate intermediate of an API (Scheme 16).^84^

**Scheme 17:**
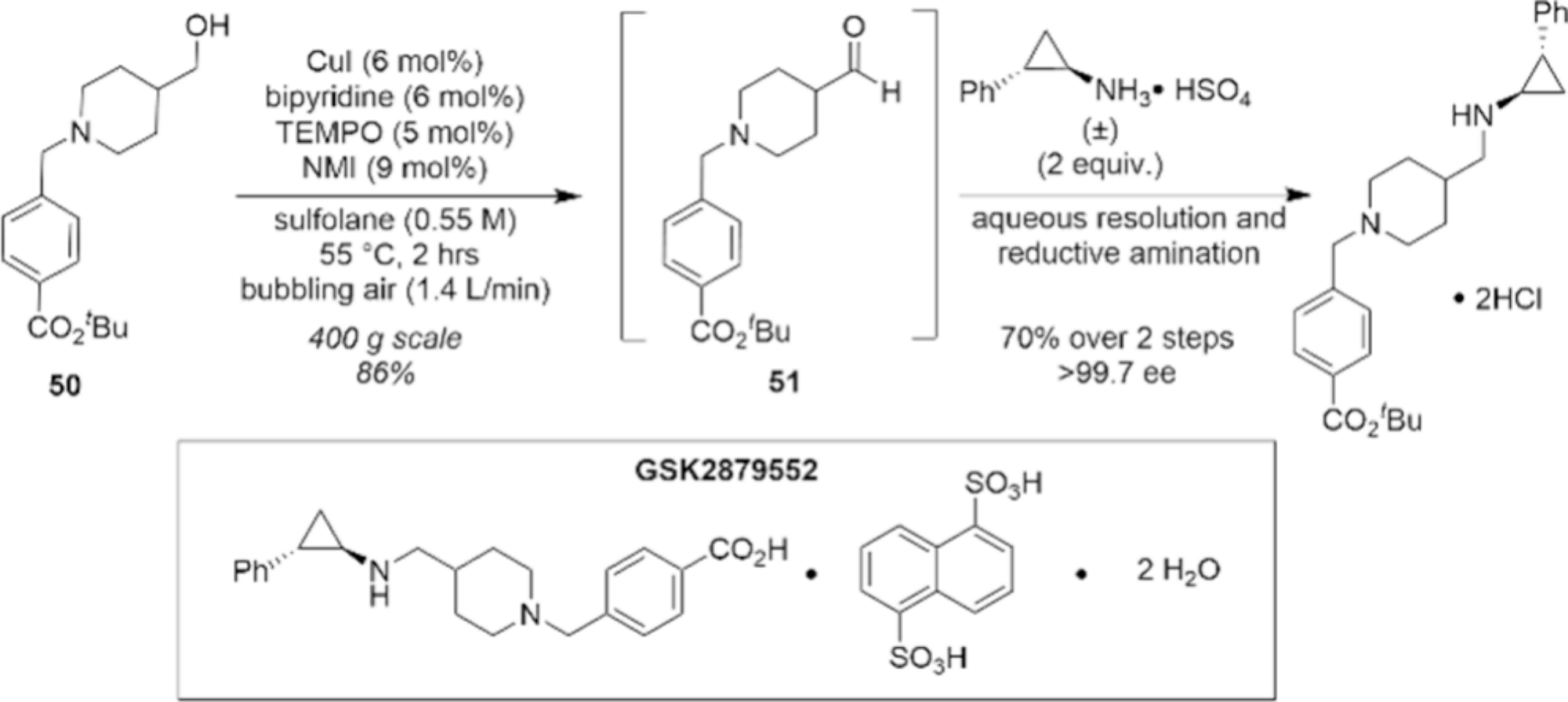
CuI/TEMPO-Catalyzed Oxidation of Alcohol 50 to Aldehyde 51 on 400-g Scale

The homogeneous reaction mixture of Cu(OAc)_2_ and bipyridine allowed the reaction to be carried out in a continuous vapor–liquid pipes-in-series reactor. In addition, the development of this oxidative coupling exemplifies a successful strategy for the implementation of aerobic oxidation in pharmaceutical manufacturing. Examples of this type of oxidation are relatively limited in this industry. Partly due to the creation of a potential flammable atmosphere composed of the mixture of oxygen gas and organic solvents. “Synthetic air” typically consists of less than 10% O_2_ in N_2_. In 2015, Stahl et al. developed a CuI/ABNO/NMI-catalyzed aerobic alcohol oxidation process. Using this protocol, functionalized primary and secondary alcohols were oxidized to aldehydes and ketones with high efficiency, suitable for use in both batch and flow processes. This catalyst system was demonstrated in a > 50 g scale batch reaction.^85^ Chemists at GSK also reported a 400 g scale aerobic oxidation synthesis of the LSD1 inhibitor GSK2879552 (Scheme 17). The starting aliphatic alcohol **50** transformed within 2 h to the corresponding aldehyde **51** facilitated by the CuI/TEMPO system. The use of sulfolane as a high flash point solvent, low cost catalyst components, and reaction concentration of >0.5 M make this method amenable to safe large-scale application in a batch reactor.^86^

Some work has been done with respect to establishing the limiting oxygen concentrations in a number of common solvents in order for these reactions to be better understood and hopefully more widely employed.^87^ One solution that could potentially allow for the safe use of 100% (pure) O_2_ is the implementation of continuous flow processes rather than batch reactions, like in the example from the Lilly laboratories.^88,89^ With increasing expertise and improving equipment available, the implementation of more and more flow processes in the pharmaceutical industry is becoming a reality, and perhaps aerobic oxidations are not far away from joining other hazardous reactions that have found a solution in flow.

**Scheme 18:**
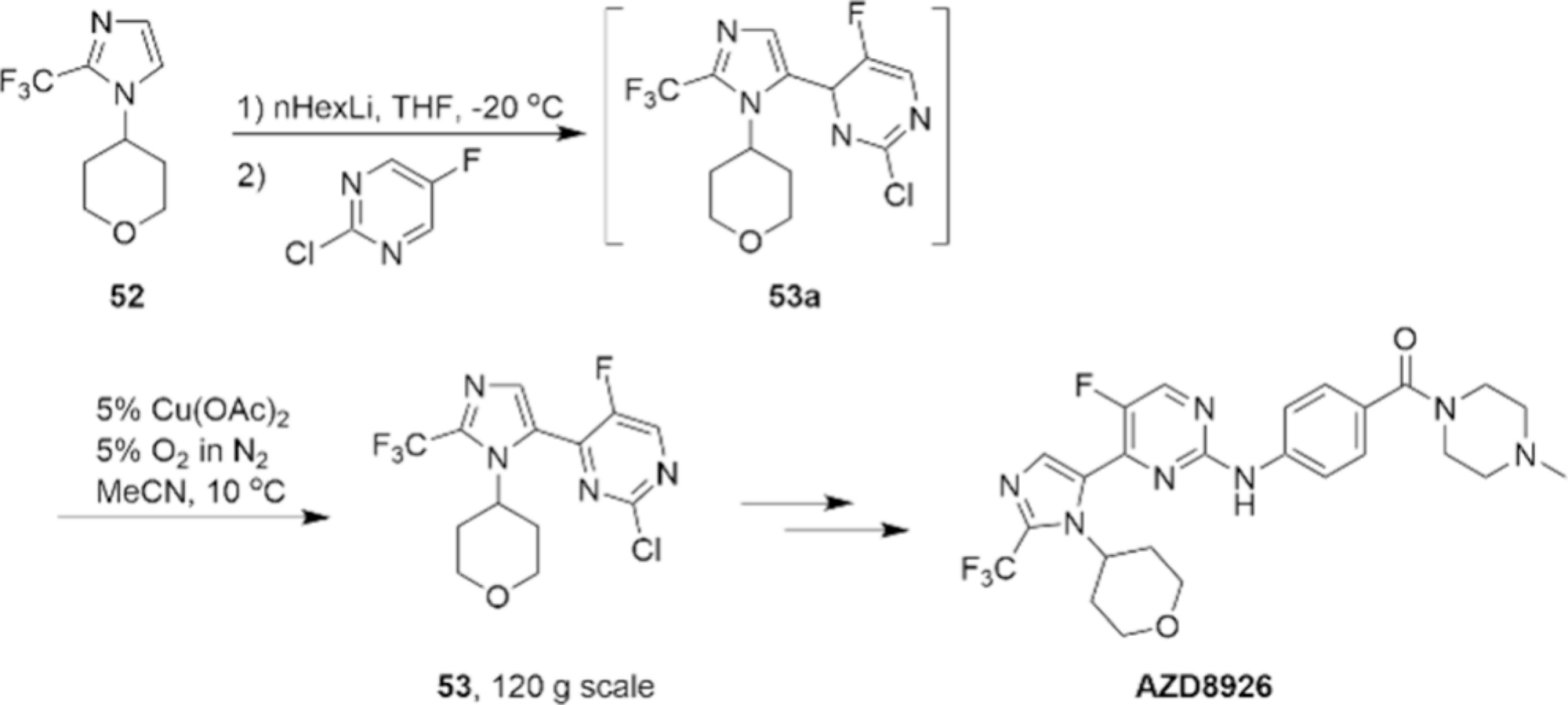
Scale-up Route to AZD8926 via Cu-Catalyzed Synthesis of the Key Intermediate 53

Another example of copper catalysis developed by a pharmaceutical laboratory is the large-scale synthesis of AZD8926, a GSK3β inhibitor (Scheme 18). A key step in the scalable route involves the copper-catalyzed dehydrogenative aromatization of the intermediate **53a**.^90^ Similarly, it was reported that using oxygen as the stoichiometric oxidant with Cu(OAc)_2_ as the catalyst is crucial to the success of this route. To remove copper from the product, a solution of 5% NH_3_ (aq.) was added (effective removal down to <50 ppm, without ammonia >2500 ppm). The mixture was cooled to 0 °C, and **53** was precipitated with an HPLC purity of 99% on a 120 g scale.

**Scheme 19:**
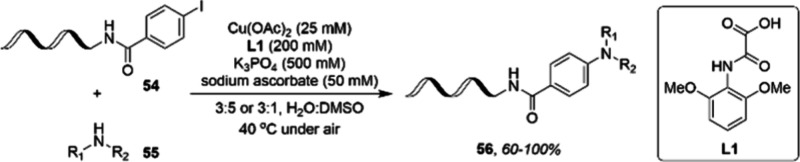
Copper-Catalyzed Amination of DNA-Conjugated Aryl Iodides 54

One interesting example of non-PGM application in industry was reported by researchers at Novartis. By means of using a series of novel oxoacetic acid ligands, they developed a copper-catalyzed cross-coupling of DNA-conjugated aryl iodides **54** with aliphatic amines **55**.^91^ The air-stable oxoacetic acid ligand **L1** ensured that the transformation proceeded in aqueous DMSO at a low temperature and in air. This makes the reaction an ideal methodology candidate for the synthesis of DNA-encoded library **56** (Scheme 19).

**Figure 11:**
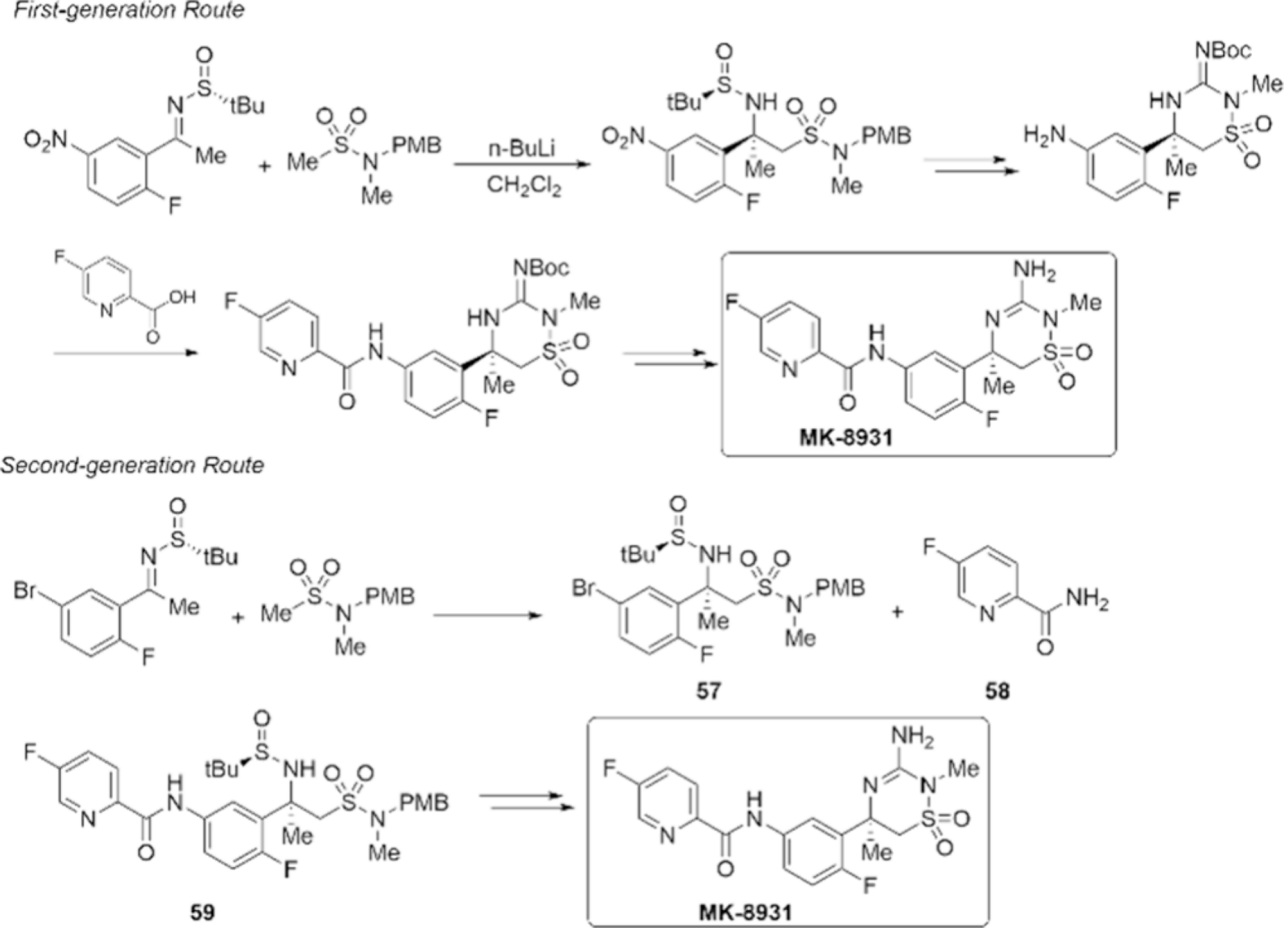
An overview of the synthesis of verubecestat MK-8931.

**Scheme 20:**
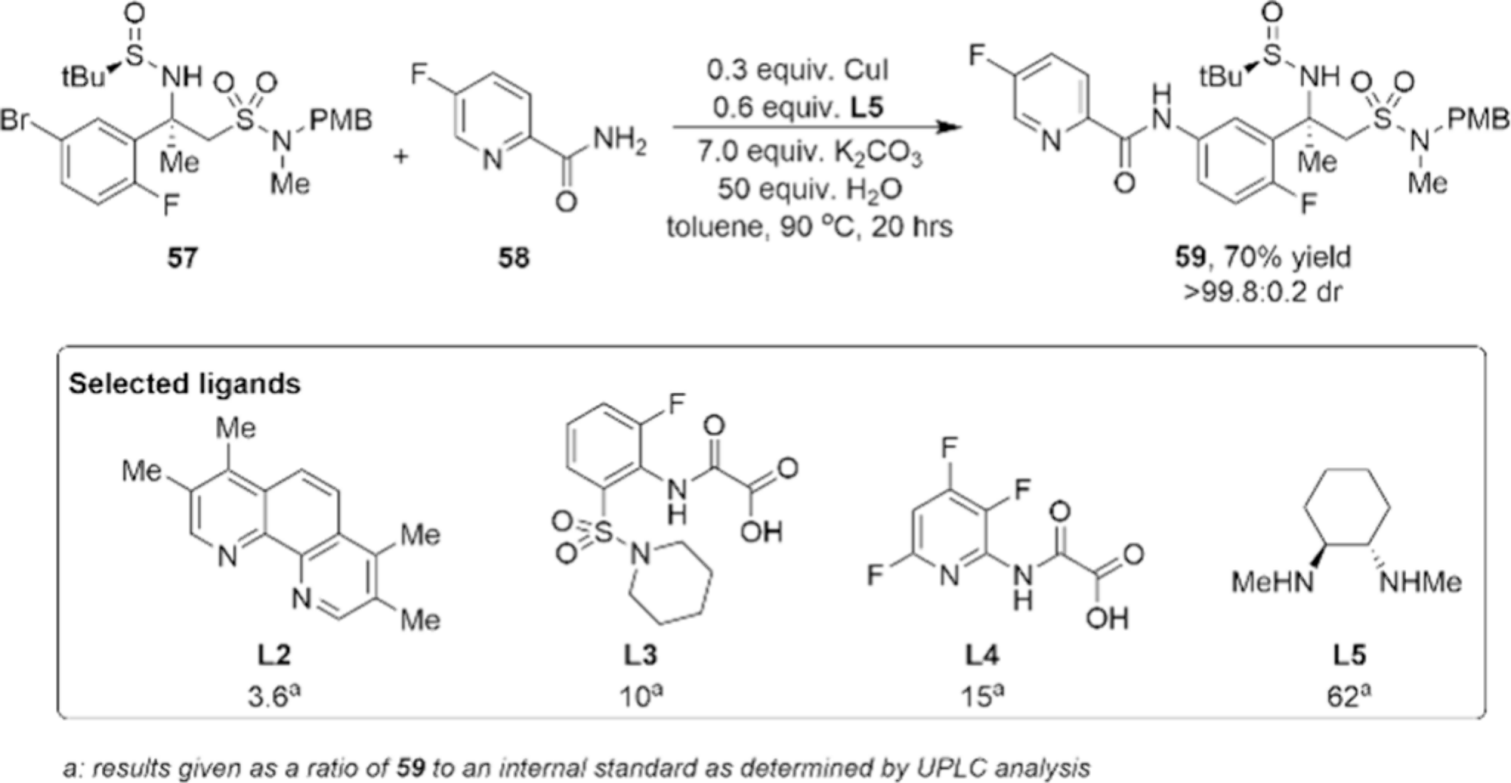
Decagram Scale Synthesis of Intermediate 59 for Verubecestat

Verubecestat (MK-8931), is an inhibitor of β-secretase and it was developed initially for the treatment of Alzheimer’s disease. Although its Phase III trials for the treatment of AD turned out to be a failure, more and more research is showing that Verubecestat has positive effects in the treatment of other ailments.^92^ As shown in Figure 11, there are two routes for the synthesis of MK-8931. The second-generation route relies on a copper-catalyzed C–N bond formation between **57** and **58** to produce intermediate **59**.^93^ High-throughput experiments demonstrated that the combination of 1,2-diamine ligand **L5** and CuI afforded the highest reactivity (Scheme 20). A carefully optimized amount of water is important for obtaining the target product since protodehalogenation of **57** is a significant undesired side reaction of the process. Additionally, solubility of the base, potassium carbonate, is also crucial. It was found that 50 equiv of water provided the best balance between maximizing reactivity and minimizing protodehalogenation. Under the optimized conditions, the C–N coupling product **59** was obtained in 80% assay yield at a decagram scale and 70% isolated yield after crystallization.

**Scheme 21:**
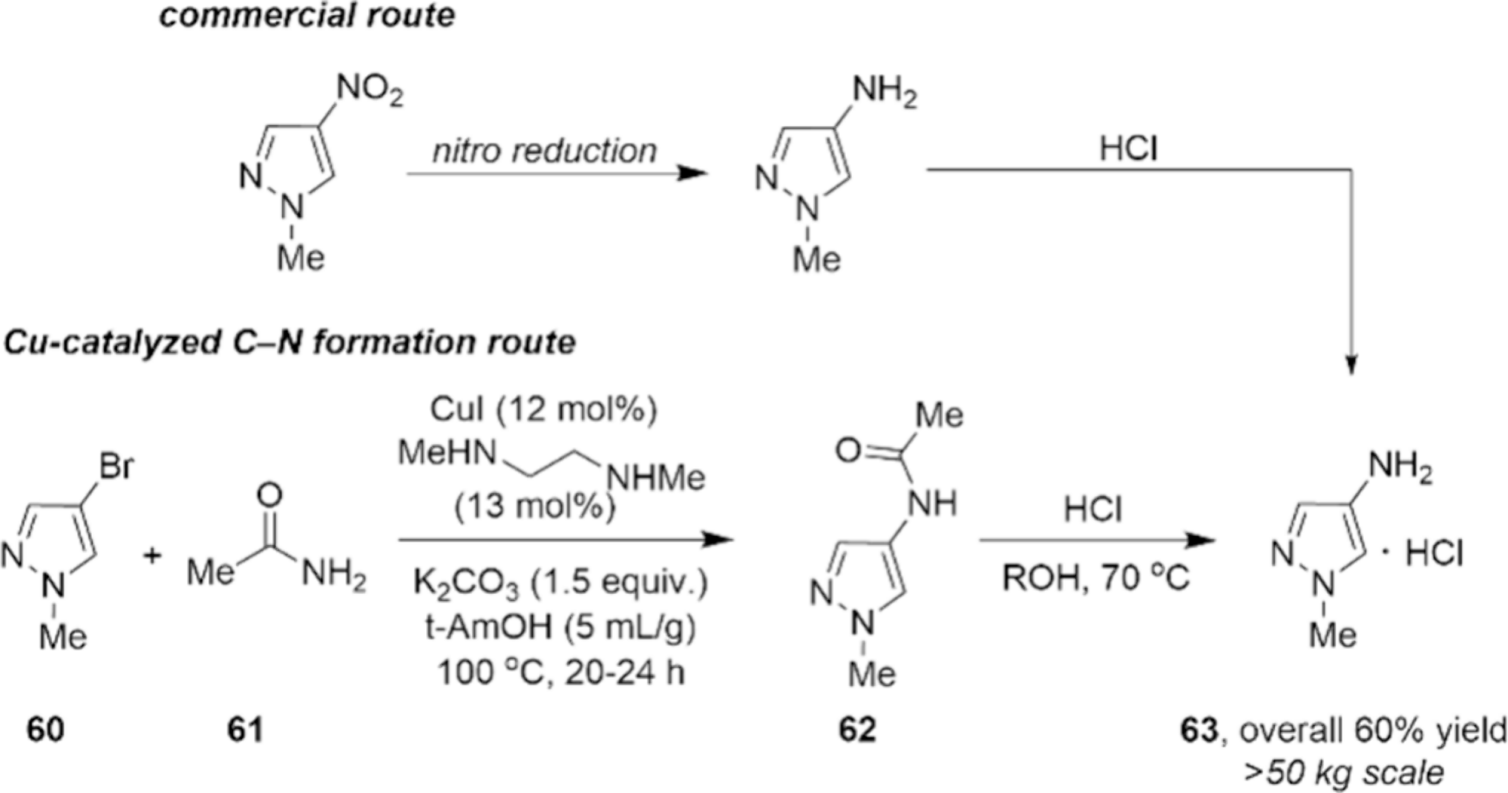
50 kg-Scale Synthesis of 63 via Cu-Catalyzed Amidation to Provide 62

Also, taking advantage of the catalyst system composed of CuI and diamine ligand, recently, a >50 kg scale synthesis route to aminopyrazole building block **63** (Scheme 21) was developed through a collaboration between Pfizer and STA Pharmaceutical.^94^ The route proceeds with the amidation reaction of bromide **60** and ammonia surrogate acetamide **61**, followed by acidic hydrolysis of intermediate **62**, producing **63** in overall 60% yield. Even though the catalyst and ligand loadings are relatively high (12 mol % and 13 mol %, respectively), this procedure provides an alternative to the standard nitration/reduction sequence and avoids energetic intermediates, specialized hydrogenation equipment, and potentially genotoxic impurities that arise from nitro reduction. Residual copper levels were found to be <10 ppm.

**Scheme 22:**
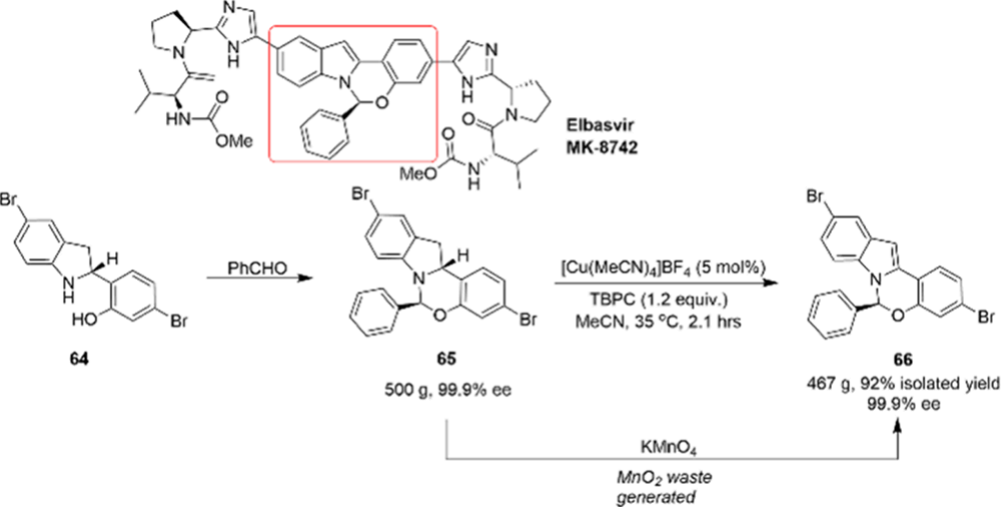
500 g-Scale Synthesis of the Key Intermediate 66 of Elbasvir

Elbasvir (MK-8742), developed in 2015 through a collaboration between MSD and WuXi Apptec, is a novel small molecule for the treatment of hepatitis C. As shown in Scheme 22, the synthetic challenge of Elbasvir lies in the construction of the central benzoxazinoindole, which contains a hemiaminal ether stereocenter. The route first reported to install the stereocenter proceeds through diastereoselective condensation of indoline **64** with benzaldehyde to yield hemiaminal ether **65**, followed by oxidative aromatization with KMnO_4_. Although this methodology can deliver indole **66** in 85% yield and >99.2% optical purity, it generates a large amount of insoluble and hazardous MnO_2_ waste. Thus, scientists at Merck developed an alternative method for the oxidation step.^95^ With a combination of [Cu(MeCN)_4_]BF_4_ as the catalyst and an organic percarbonate TBPC as the stoichiometric oxidant, the reaction proceeds without any byproducts and with good conversion and enantioselectivity. The procedure was successfully applied on a 500 g-scale for the synthesis of **66**. To a certain extent, this methodology maximizes product yield and minimizes the environmental footprint of this commonly used synthetic transformation.

**Figure 12:**
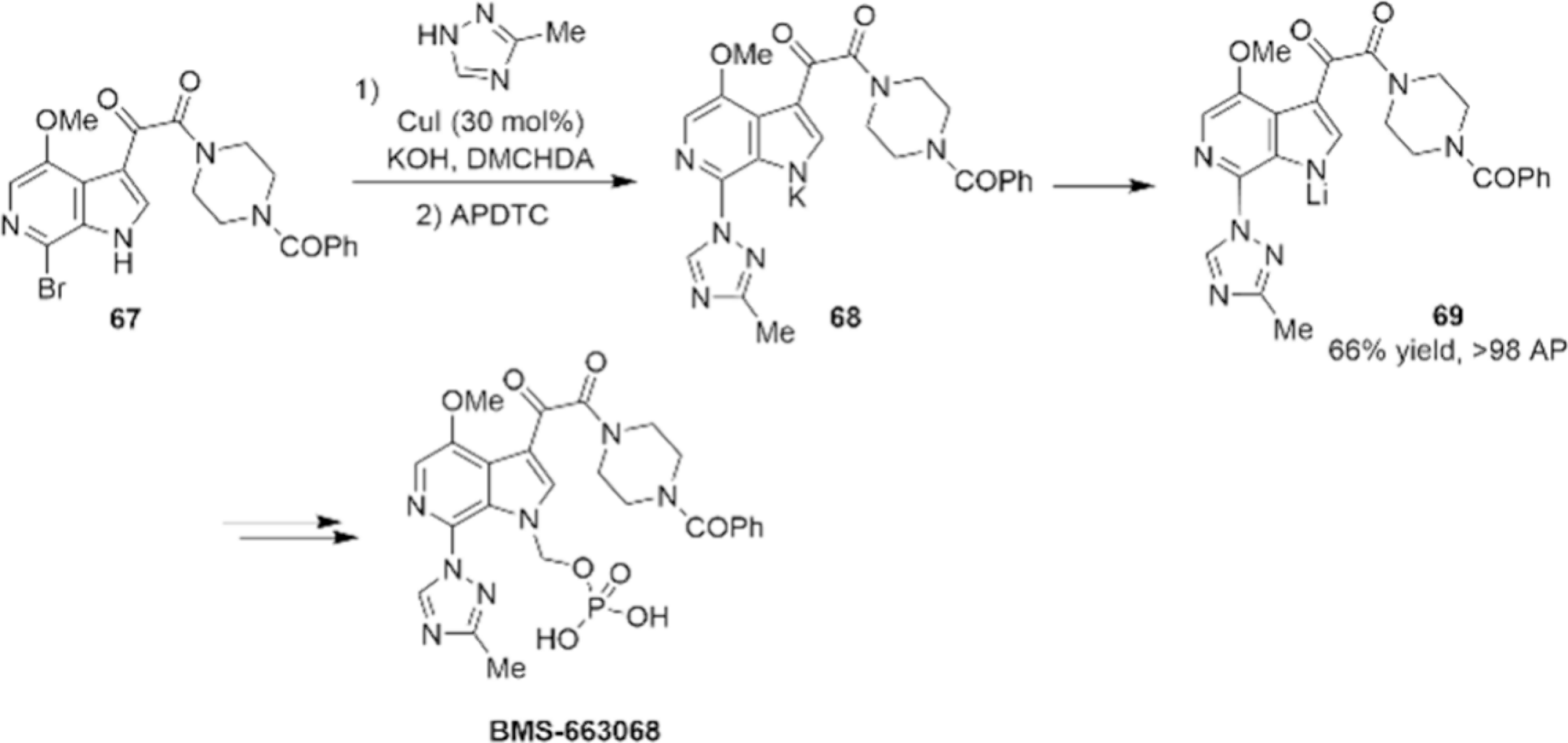
Commercially viable (hundreds of kg scale) synthesis of an intermediate toward BMS-663068 (DMCHDA = dimethylcyclohexane-1,2-diamine; APDTC = ammonium 1-pyrrolidinedithiocarbamate).

The catalyst loading of any reaction is a very important parameter for the process chemist to consider when choosing a metal catalyst. A high loading of a non-PGM catalyst, just as with a PGM catalyst, will make it difficult to remove metal impurities, which is extremely important for API production. An example representing a balance between the amount of catalyst used and choice of PGM versus non-PGM catalyst is the commercially viable hundreds of kg scale synthesis of the intermediate of BMS-663068 (Figure 12),^96^ a HIV attachment inhibitor prodrug. The CuI loading in the C–N coupling step is up to 30 mol %, but the adoption of APDTC as copper scavenger can decrease the level of residual copper to an acceptable level. An innovative salt metathesis promoted the isolation of **69**, which could be isolated via a facile filtration process because of its solid-state properties. The final processing conditions resulted in the isolation of **69** in good yield (66%) with excellent quality (>98 area%, > 96 wt %).

**Scheme 23:**
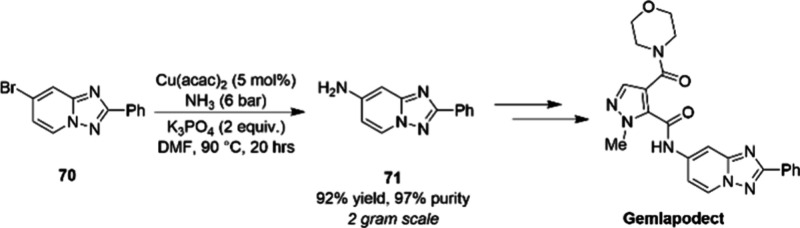
Copper-Catalyzed C–N Bond Formation (Gram Scale) in the Synthesis of Gemlapodect

Recently, the Roche R&D group described another gram scale copper-catalyzed C–N bond-forming reaction, namely a selective monoamination reaction of an aryl bromide using gaseous NH_3_ (Scheme 23), as part of route scouting to synthesize gemlapodect.^97^ Although impressive results were achieved, the toxicity associated with aniline intermediate **71** led the team to develop an alternative route.

**Scheme 24:**
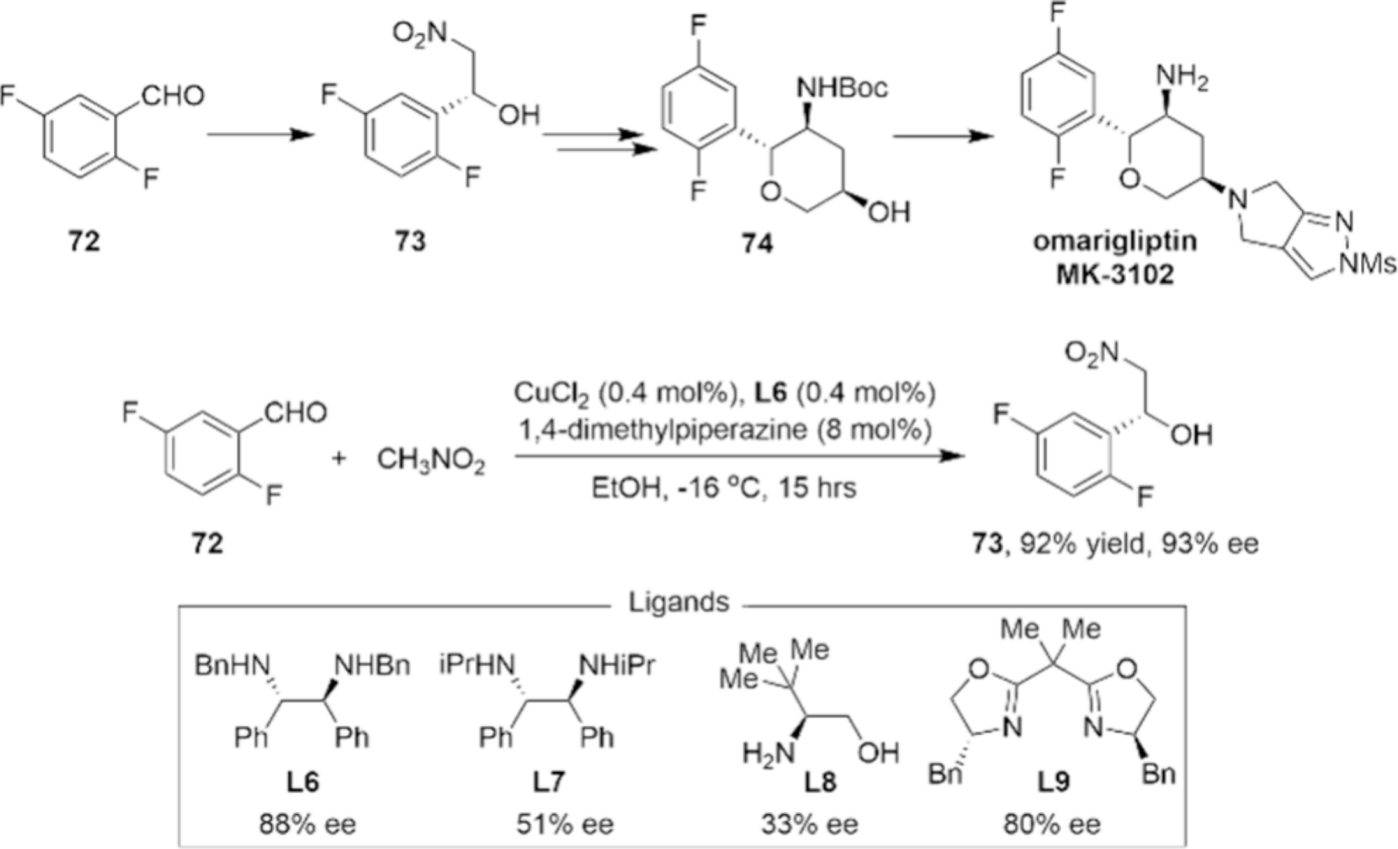
Kilogram-Scale Practical Asymmetric Henry Reaction Catalyzed by Cu-Diamine Complex

Scheme 24 describes a highly efficient precious-metal-free synthesis of a key tetrahydropyranol intermediate of DPP-4 inhibitor omarigliptin MK-3102.^98^ The synthesis of MK-3102 utilizes the simple starting material **72** and proceeds in four linear steps. Intermediate **73** was synthesized via an optimized asymmetric Henry reaction that takes advantage of 0.4 mol % of both CuCl_2_ and **L6** as the catalyst system, affording **73** in 92% yield with 93% ee within 15 h. The following one-pot nitro-Michael–lactolization–dehydration telescoped process results in **74**. These conditions were successfully demonstrated on kg scale.

**Scheme 25:**
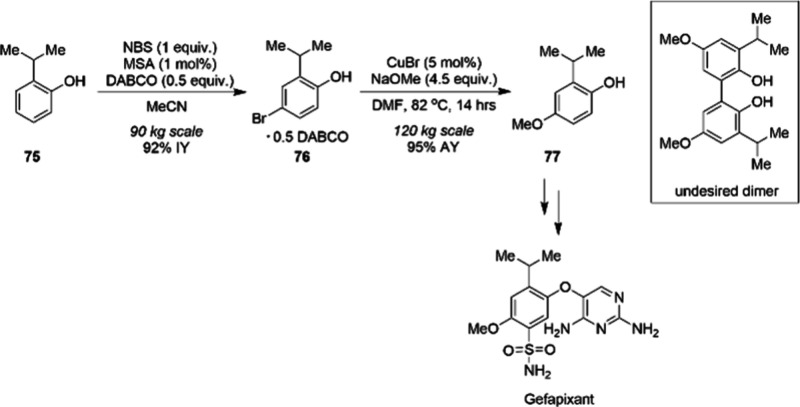
Copper-Catalyzed Methoxylation of the Bromophenol-DABCO Cocrystals 76 to Provide a Robust and Cost-Efficient Process to 77, an Early Intermediate in the Synthesis of Gefapixant

Scientists at Merck leveraged a copper-catalyzed C–O coupling for the synthesis of the important intermediate **77** in the manufacturing process of Gefapixant used in treatment of chronic cough.^99^ The authors developed a *para*-bromination protocol of the commodity chemical 2-isopropylphenol **75** which was then efficiently converted to aryl methyl ether **77** using methoxide under copper catalysis (Scheme 25). The purity of **76** had a profound influence on the success of the copper-catalyzed reaction. The authors identified crystallization of **76** with DABCO to provide a suitable purity to ensure reproducibility, thereby enhancing the robustness of the methoxylation step. Importantly, bromophenol-DABCO cocrystals **76** were also found to suppress the formation of an oxidative dimerization byproduct, which otherwise formed when the unprotected phenol was used under copper catalysis. The route was performed on a 120 kg scale to afford intermediate **77** containing just 11 ppm copper.

**Scheme 26:**
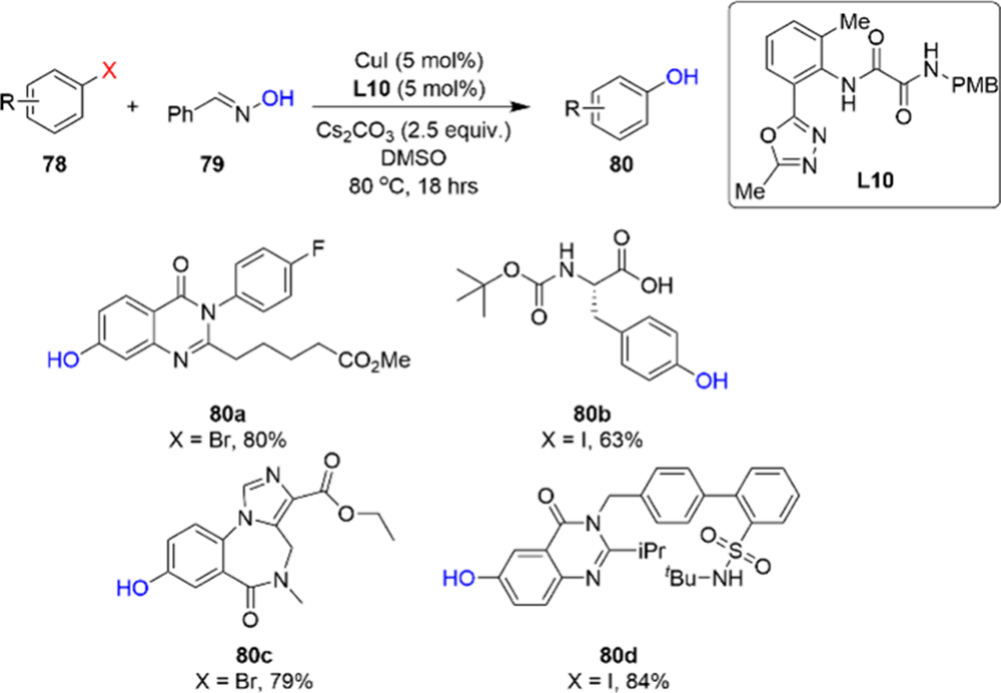
Late-Stage Milligram-Scale Synthesis of Numerous Drug-Like Phenols

The late-stage introduction of a phenol is important in certain cases due to instability of an intermediate or a lack of starting material. Scientists at Merck developed a catalyst system composed of ligand **L10** and CuI salt, with the goal of synthesizing phenols **80** (Scheme 26).^100^ Ligand **L10** was found to be the most effective through a combination of high-throughput experimentation, mass-directed ligand library purification, and rational ligand evolution. The catalyst system ensured a mild milligram scale copper-catalyzed reaction for the synthesis of phenols with a traceless hydroxide surrogate **79**, and therefore enabled the late-stage synthesis of numerous drug-like phenols.

**Scheme 27:**
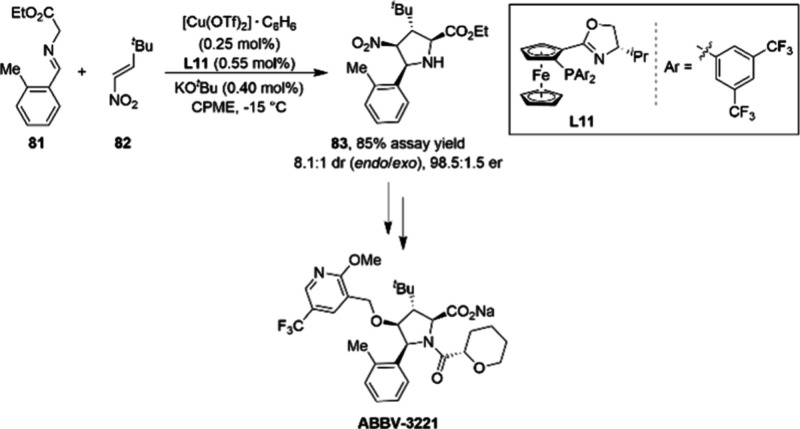
Kilogram-Scale Cu(I) Catalyzed [3 + 2] Cycloaddition Reaction in the Synthesis of ABBV-3221

A reaction class where enantioselective copper catalysis has proven to be enabling is the [3 + 2] cycloaddition, where copper serves as an effective chiral Lewis acid. Researchers at AbbVie developed such a method to access multikilograms of the pyrrolidine core of ABBV-3221 (Scheme 27).^101^

The cycloaddition reaction was found to proceed with the highest stereoselectivity, yield, and reproducibility using the impressively low loading of 0.25 mol % Cu(OTf)_2_·C_6_H_6_ and 0.55 mol % of ligand **L11**. This process was successfully implemented on the pilot plant to produce two 20 kg batches of cycloaddition product **83**. A critically important aspect of the scale-up campaign was controlling the exotherm of the reaction to maintain high enantioselectivity. This was achieved by diluting the nitro-olefin in CPME as well as increasing the addition time of this reactant from 0.5 to 3.5 h.

**Scheme 28:**
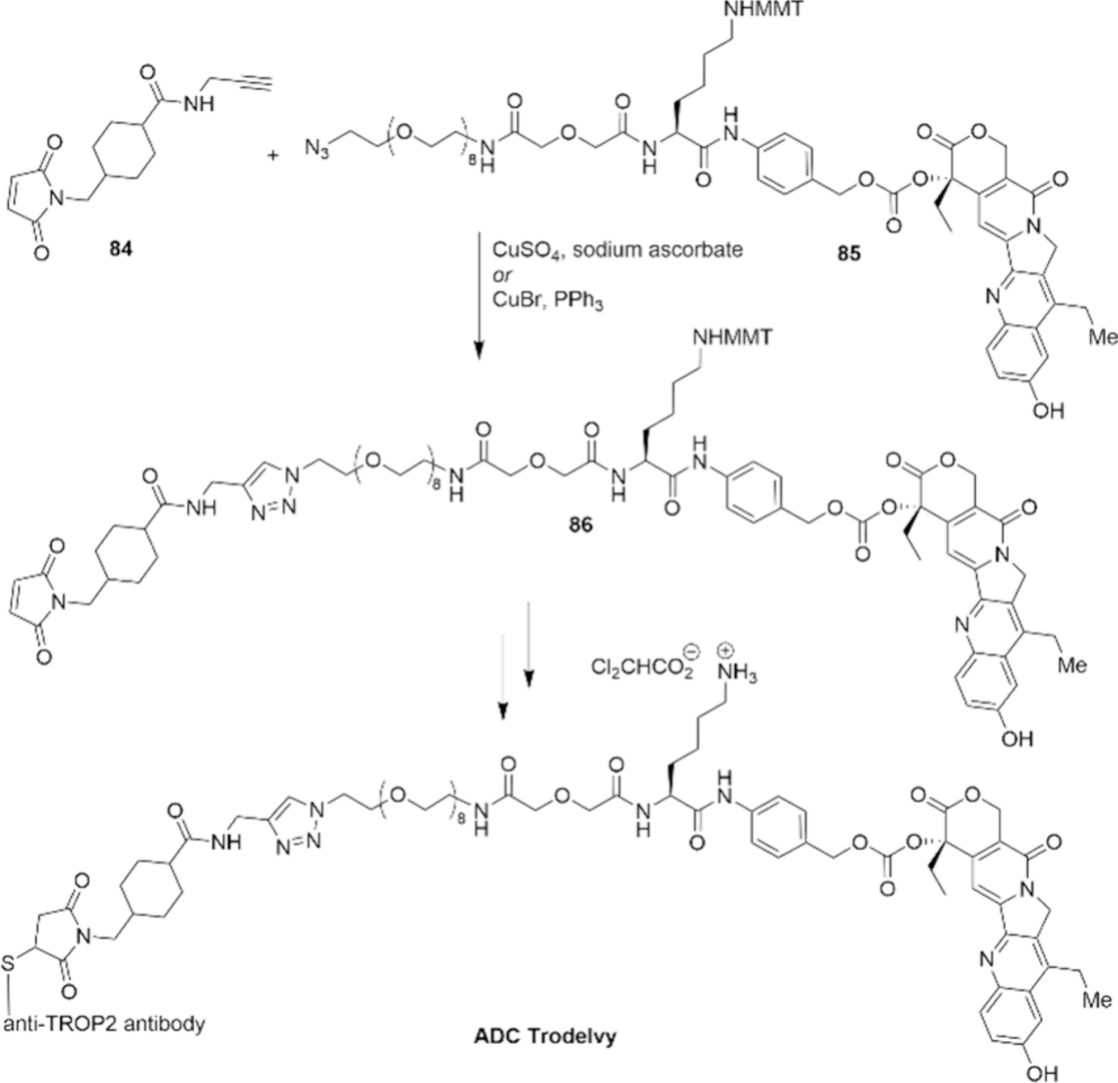
Cu-Catalyzed Click Reaction for the Assembly of the Linker in ADC Trodelvy at Milligram Scale

The 2022 Nobel Prize in Chemistry was awarded for the development of click chemistry and bioorthorgonal chemistry. One example of an industrial application of this reaction is in the synthesis of antibody-drug conjugate Trodelvy which was approved by the FDA in 2020. A key step in the synthesis of the appropriate linker between the cytotoxin and the antibody is the copper-catalyzed click reaction (Scheme 28).^102^ Employing this copper-catalyzed reaction allowed for rapid attachment of the maleimide group **84** before the drug conjugate was attached to the antibody.

**Scheme 29:**
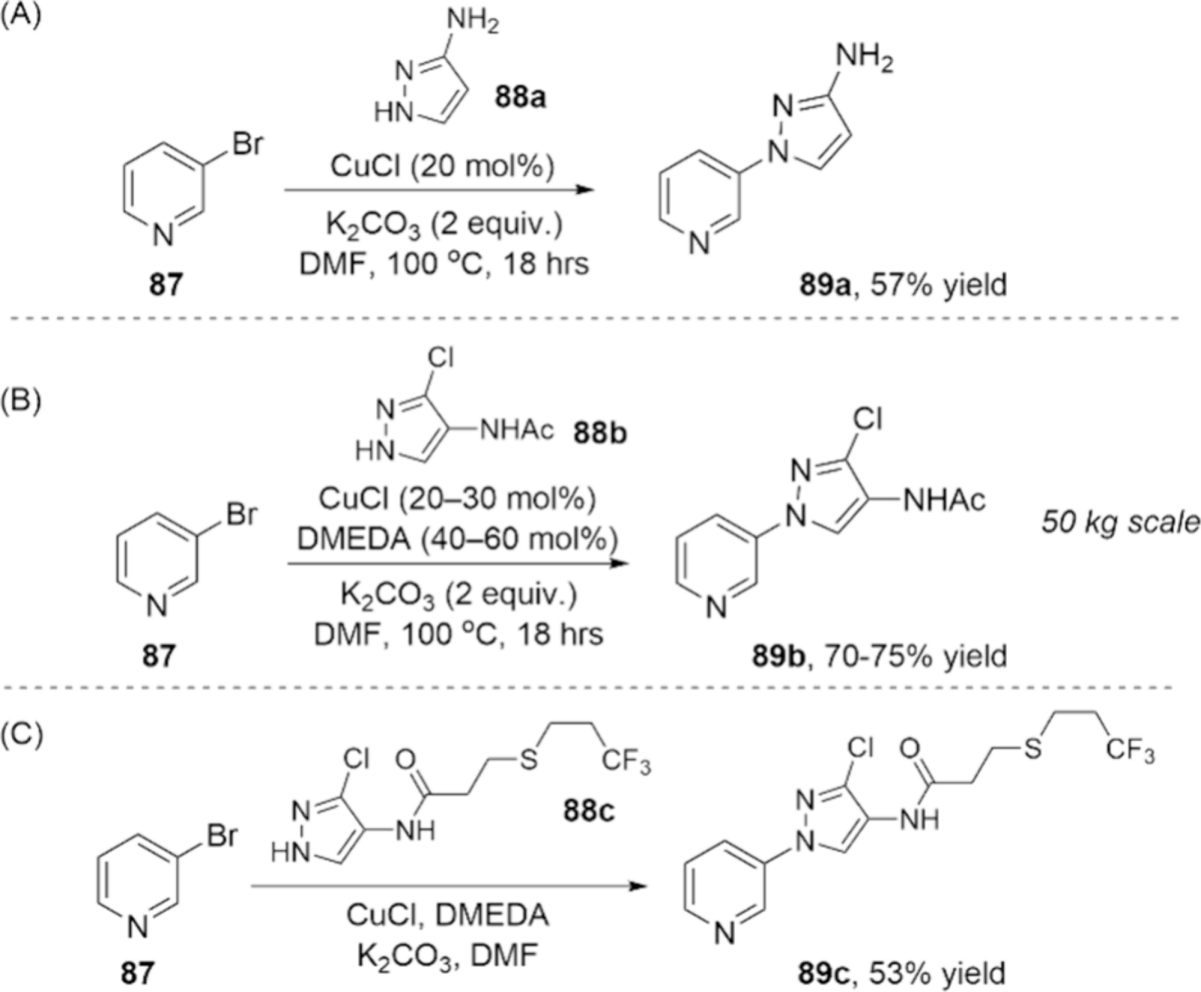
Ullman Couplings Studied during Development of an Agrochemical Manufacturing Process

In the agrochemical industry, production scales are typically much greater than those of pharmaceuticals at similar stages of development. Thus, optimized agrochemical processes tend to require very low catalyst loadings when homogeneous PGM catalysts are used. Non-PGM catalysts are an attractive alternative, which can obviate the need to develop coupling reactions that proceed with ultralow palladium loadings (e.g., for C–N couplings). An interesting case study was recently reported by Li and co-workers at Corteva, during development of the manufacturing process for the insecticide tyclopyrazoflor.^103^ To form a key pyridine–pyrazole bond, three Ullman couplings were studied (Scheme 29).

Initially, the authors considered an early stage coupling of bromopyridine with an aminopyrazole as part of the proposed route (Scheme 29A). The reaction proceeds effectively with CuCl, since the aminopyrazole can serve as a ligand. Approximately 10% of the regioisomer resulting from coupling at the primary amine was observed, but this impurity could be purged by crystallization. Despite the moderate yield, both starting materials and the catalyst were very economical, and the reaction was viewed as a successful proof of concept.

Next, a second route was developed with a midstage Ullman coupling (Scheme 29B). A ligand was required to promote this reaction. The authors successfully performed the reaction on 80 kg scale using DMEDA and CuI. The conditions were further optimized using CuCl in place of CuI to reduce iodide waste and lower the cost. The authors found that L/Cu ratios of 2:1 to 5:1 afforded high conversion at <10 mol % CuCl. Impurities resulting from arylation of the acetamide group were well-controlled during the process and purged during the isolation from DMF/water. These conditions were scaled up to multiple 50 kg batches, affording 70–75% yield of the isolated product in high purity. The authors also considered a later-stage Ullman coupling, such as the reaction shown in Scheme 29C. Here, the reaction yields were significantly lower than the midstage Ullman. As is often the case in pharmaceutical process development, the greater cost of the starting materials going into a late-stage step often means that yield is prioritized over the cost of the catalyst.

Ultimately, the route featuring the midstage Ullman coupling (Scheme 29C) was selected for further development. This decision was driven by a holistic evaluation of the process cost, safety, and efficiency, although the performance of the copper catalyst played a key role. The low cost, forgiving L/M ratio, and facile purge of DMEDA continue to make it a privileged ligand for large-scale Ullman couplings.

**Scheme 30:**
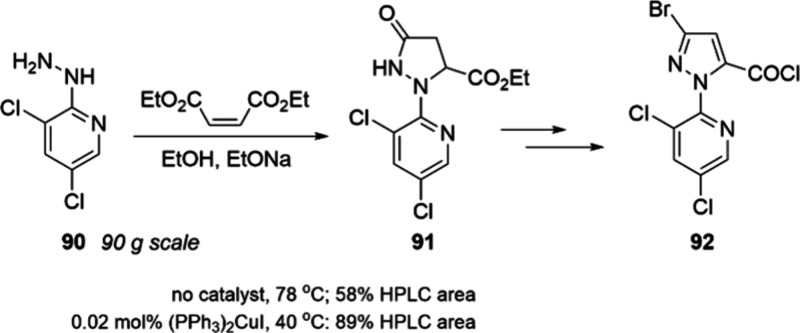
Copper-Catalyzed Cyclization of Arylhydrazine 90 with Diethyl Maleate

A novel application of copper catalysis for an alternative synthesis of arylated pyrazoles was recently reported by Yu and co-workers at Shenyang Sinochem Agrochemicals.^104^ The authors needed to conduct the cyclization of a hydrazine with diethyl maleate to generate a pyrazole precursor for the insecticide tetrachlorantraniliprole **92** but obtained <50% yield under the standard conditions (Scheme 30). They reasoned that previously reported examples of copper- or palladium-catalyzed addition of anilines to activated olefins could be amenable to this reaction.^105^ After screening a range of palladium, copper, and nickel complexes, the authors arrived at (PPh_3_)_2_CuI as the optimal catalyst, in terms of activity and cost. This complex provides an impressive rate acceleration at <0.1 mol %. Based on the authors’ observations and related literature, it appears that the role of copper is to promote an intramolecular conjugate addition. The reaction was successfully performed on a 0.5 mol scale using 0.03 mol % (PPh_3_)_2_CuI, affording the pyrazole precursor **91** in 81% isolated yield. This example serves as a reminder that copper complexes can be cost-effective promoters of challenging conjugate additions.

**Scheme 31:**
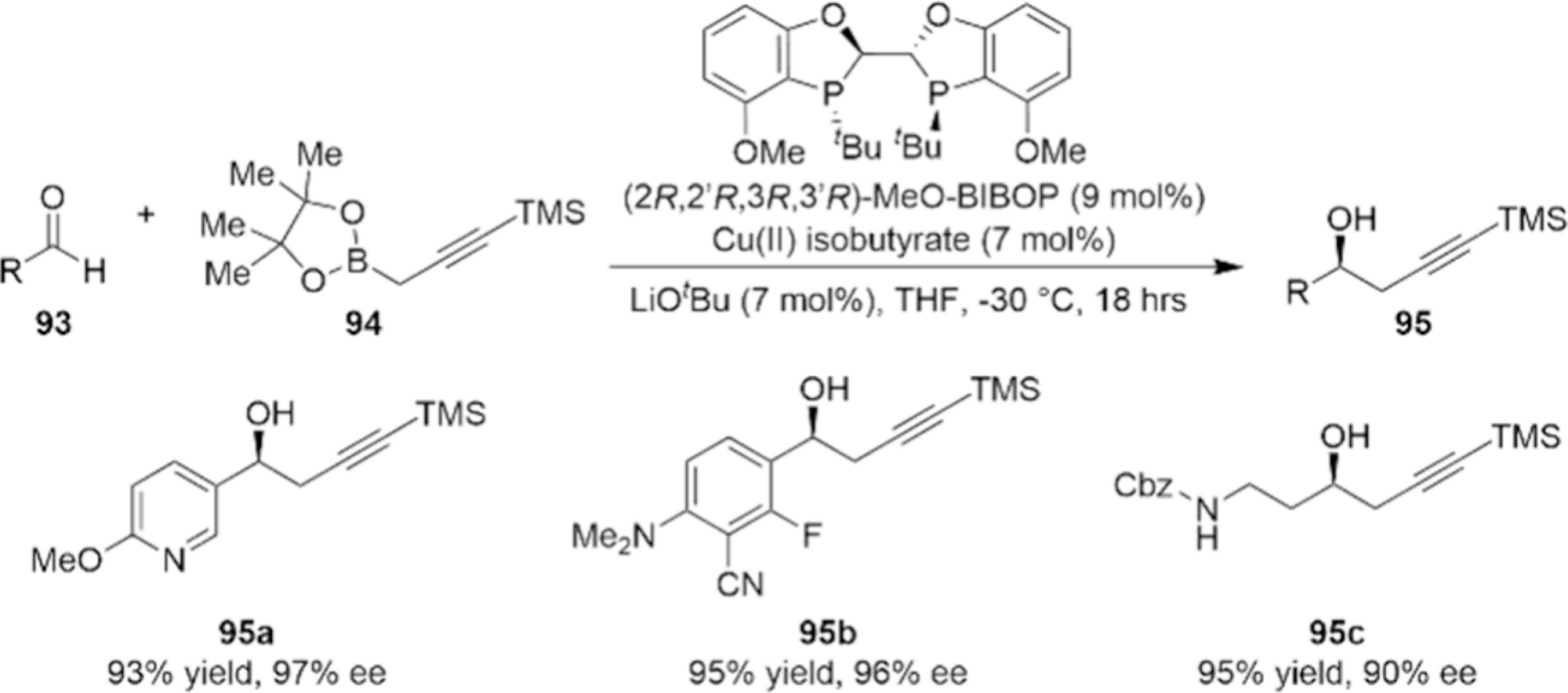
Copper-Catalyzed Propargylation of Aldehydes 93 at Milligram Scale

Finally, a reaction that merits discussion although no scale-up efforts can be found in the literature to date, is the copper-catalyzed enantioselective propargylation of aldehydes that was reported by researchers at Boehringer Ingelheim (Scheme 31).^106^ The resulting chiral homopropargylic alcohols (**95**) could be useful intermediates in the synthesis of pharmaceutically interesting compounds due to the pendant alkyne functional group, which can be further derivatized through coupling reactions or other transformations. The team at Boehringer Ingelheim demonstrated a relatively broad substrate scope, using 7 mol % Cu(II) isobutyrate and 9 mol % of the chiral ligand MeO-BIBOP. These numbers would quite likely require optimization to reduce loadings, were this reaction to be scaled up. Nevertheless, it provides a good example to illustrate the capabilities of chiral copper catalysts in assembling versatile enantioenriched building blocks.

### 2.3 Cobalt

**Scheme 32:**
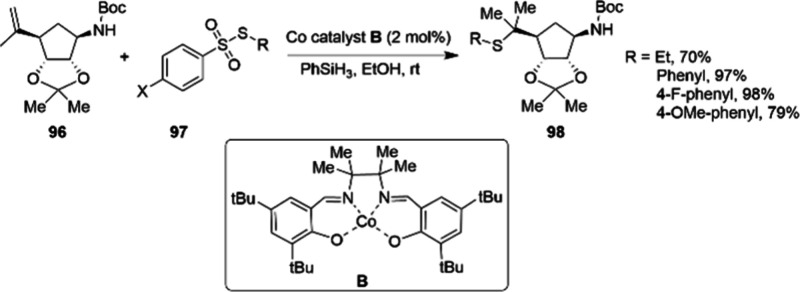
Small-Scale Cobalt-Catalyzed Synthesis of Tertiary Alkyl/Aryl Sulfides 98

**Scheme 33:**
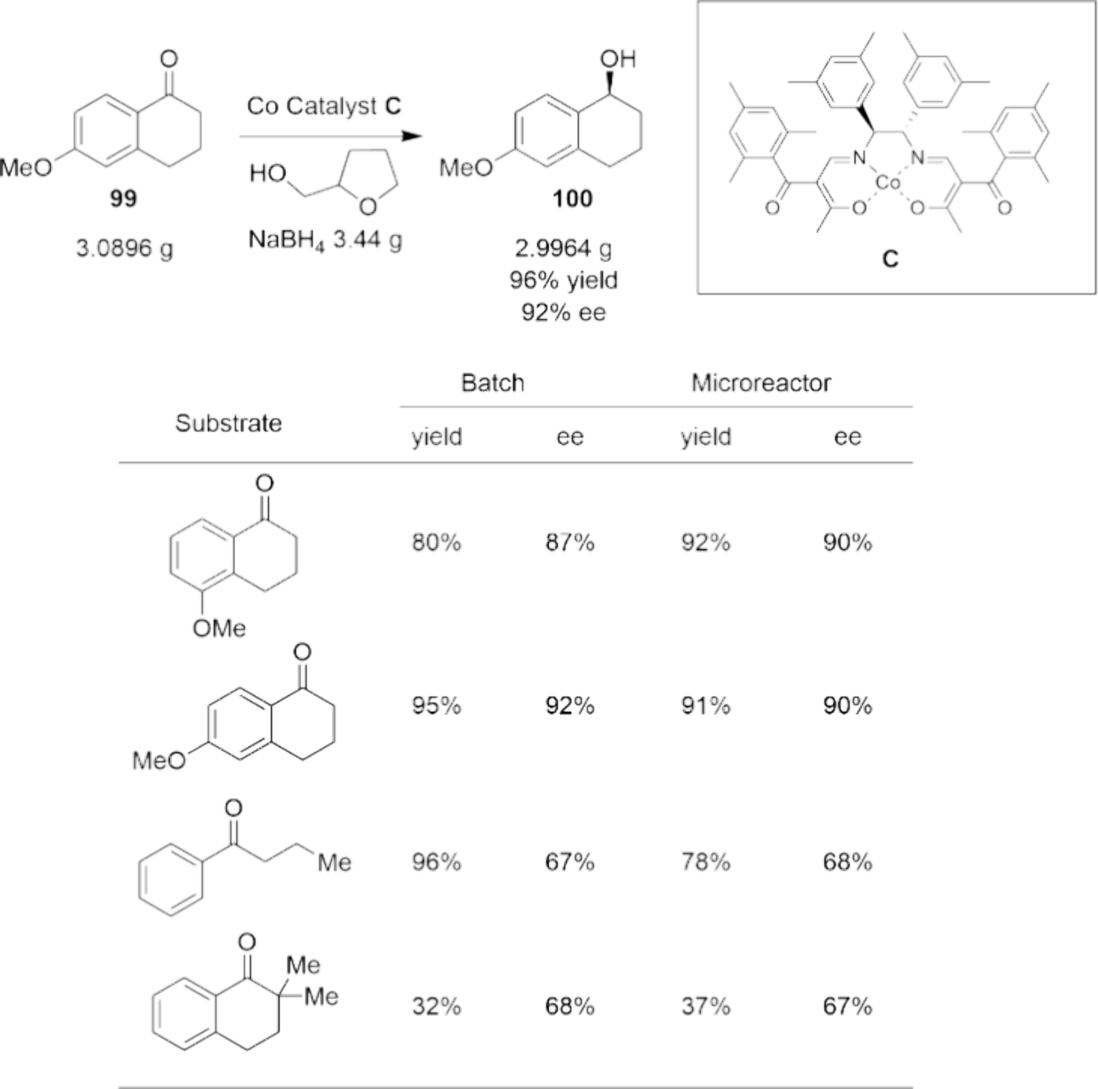
Cobalt-Catalyzed Gram-Scale Borohydride Reduction of Tetralone Derivative 99 under Continuous-Flow Conditions

In comparison to the number of applications of nickel and copper catalysis in industry, examples using cobalt catalysis are uncommon. One reason could be the lack of specialized ligands that offer sufficiently high selectivity for the broad assortment of substrates encountered in pharmaceutical research. Another factor could be that the difficulty of removing any cobalt residue from the final product is a concern for large-scale industrial production. While there are well-known sustainability challenges with respect to the scale of cobalt production required for the lithium-ion battery industry, cobalt still remains a more economical alternative to PGM catalysts for the foreseeable future. Hence, chemists in industry are still paying attention to the field of cobalt catalysis. As early as in 2011, scientists at Merck reported a mild route, using unactivated alkenes as substrates and salen-cobalt complex **B**(107) as the catalyst to synthesize tertiary alkyl/aryl sulfides (**98**) in a regioselective manner (Scheme 32).^108^ The reaction works with both electron-deficient and rich sulfur electrophiles as well as different types of substituted alkenes, resulting in the corresponding alkyl aryl sulfides (**98**) in 70–98% yield. The reaction demonstrated a broad substrate scope; however, no scale-up experiment was reported. Nevertheless, the potential of cobalt catalysis in real-world applications is evident. In 2012, Hitachi reported a gram-scale cobalt-catalyzed enantioselective borohydride reduction of **99** to **100**, taking advantage of a continuous-flow system (Scheme 33).^109^ Because of the intrinsic exothermic features of this reaction, precise temperature control is important for maintaining high enantioselectivity. The use of a microreactor ensured the high temperature required for the reaction was maintained and shortened the residence time to 12 min, thus maintaining the enantioselectivity at 92% ee while maintaining a high yield.

**Scheme 34:**
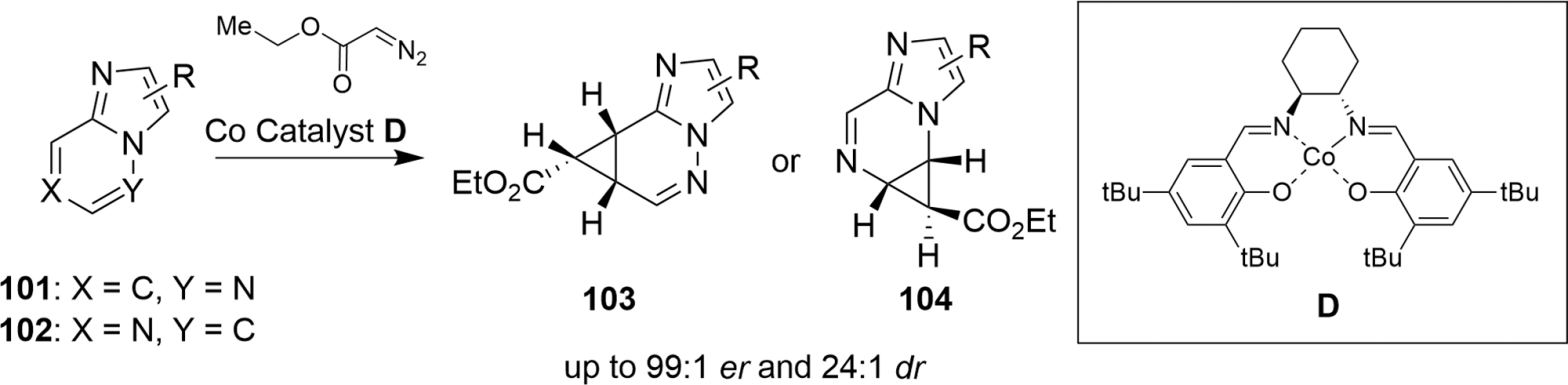
Cobalt-Salen-Catalyzed Milligram-Scale Asymmetric Cyclopropanation of Fused Heteroaromatics 101 and 102

Besides the above-mentioned C–S bond formation and borohydride reduction reactions promoted by cobalt, Co-salen catalysts also enable asymmetric cyclopropanation of fused electron-deficient azaheteroarenes.^110^ As shown in Scheme 34, Co-salen catalyst **D** promoted the transformation of heteroaromatic derivatives **101** and **102** into the corresponding cyclopropane products **103** and **104**, respectively, with high diastereomeric and enantiomeric ratios up to 99:1 er and 24:1 dr. The cyclopropane derivatives can be further functionalized to provide complex heterocyclic building blocks.

**Scheme 35:**
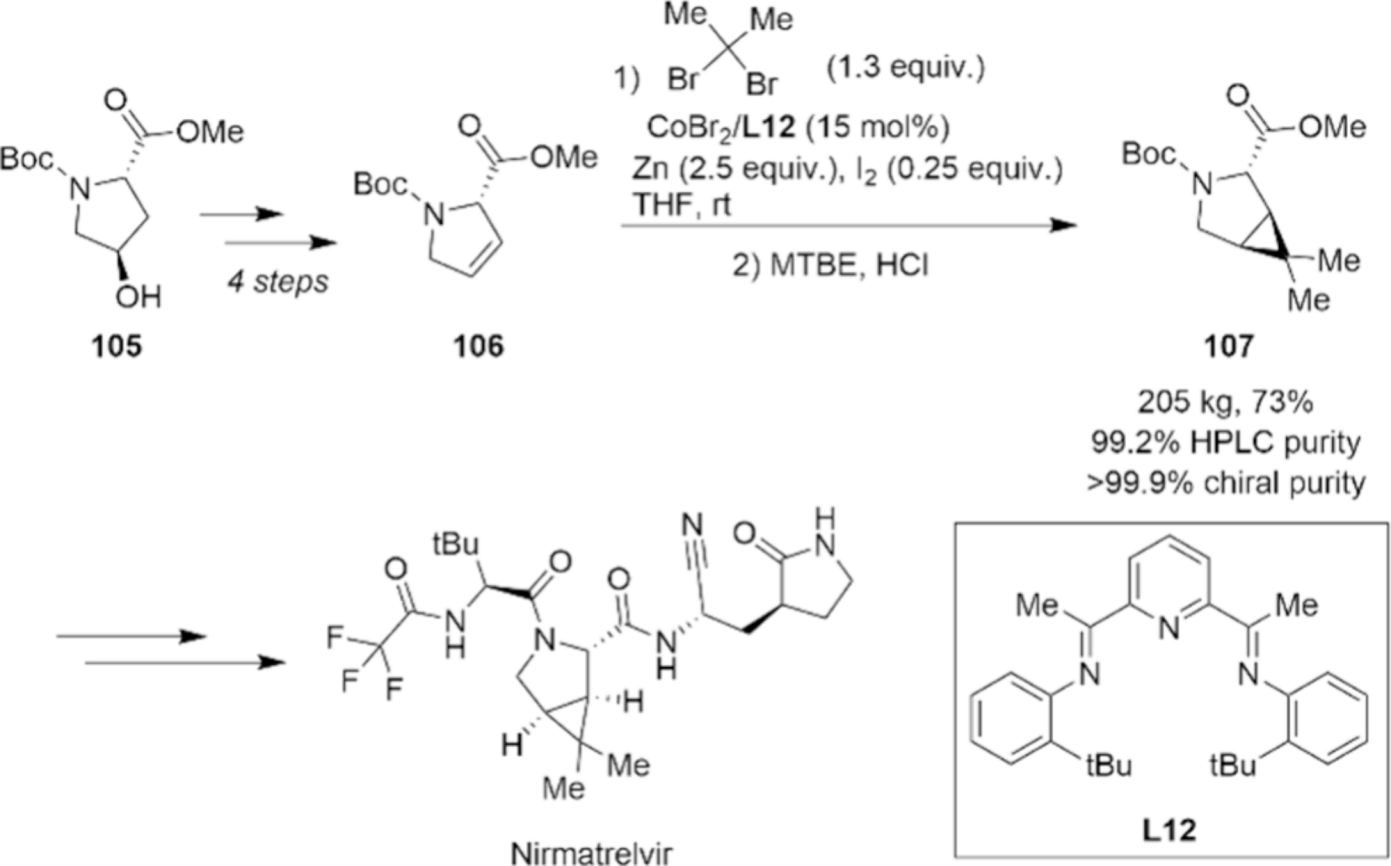
Scalable Cobalt-Catalyzed Cyclopropanation of 106 to Obtain Key Intermediate 107 for the Synthesis of Nirmatrelvir

An example of an academic methodology using non-PGMs that was quickly translated into a large-scale process is the cobalt-catalyzed cyclopropanation reported by the Uyeda group.^111^ A collaborative team between Pfizer and Curia found the method to provide an intriguing alternative route to key intermediate **107** in the synthesis of the antiviral medication Nirmatrelvir used in treatment of SARS-CoV-2.^112^ Applying this methodology allowed the synthesis to start from readily available and inexpensive material **105** (Scheme 35). Upon optimization, the authors found that employing zinc with an iodine activator circumvented the need for the zinc bromide additive which had originally been reported. Employing ligand **L12**, full conversion of **106** to **107** was achieved, thereby expediting the purification. Detailed studies of the fate of the cobalt catalyst found that the ligand suffers from decomposition under the reaction conditions necessitating a catalyst loading of 15 mol %. These findings point to an opportunity for future improvements in catalyst design. Application of the current catalytic system proceeds on 205 kg scale in 73% yield over two steps and furnished the desired product in high purity.

**Scheme 36:**
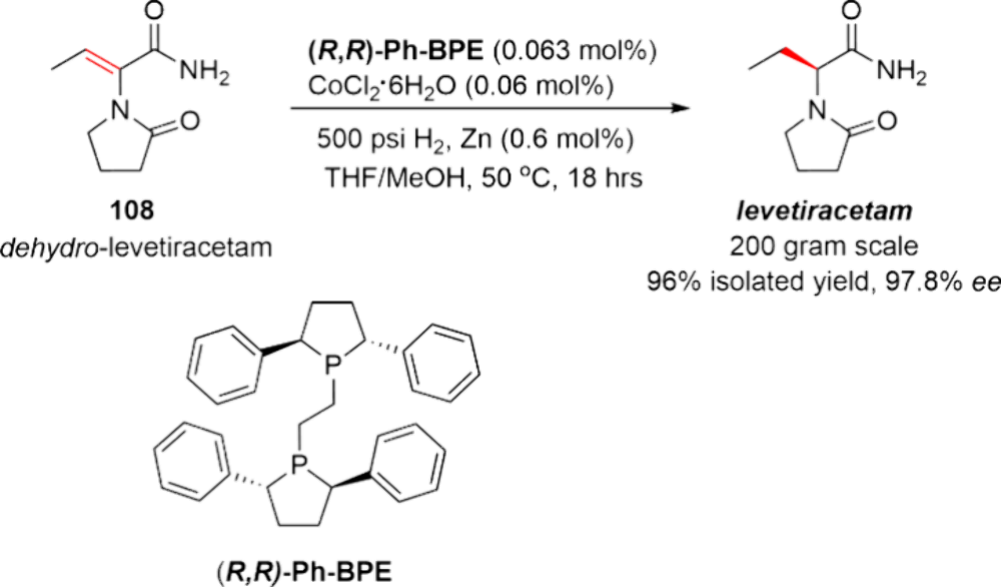
Gram-Scale Synthesis of Epilepsy Medication Levetiracetam

**Scheme 37:**
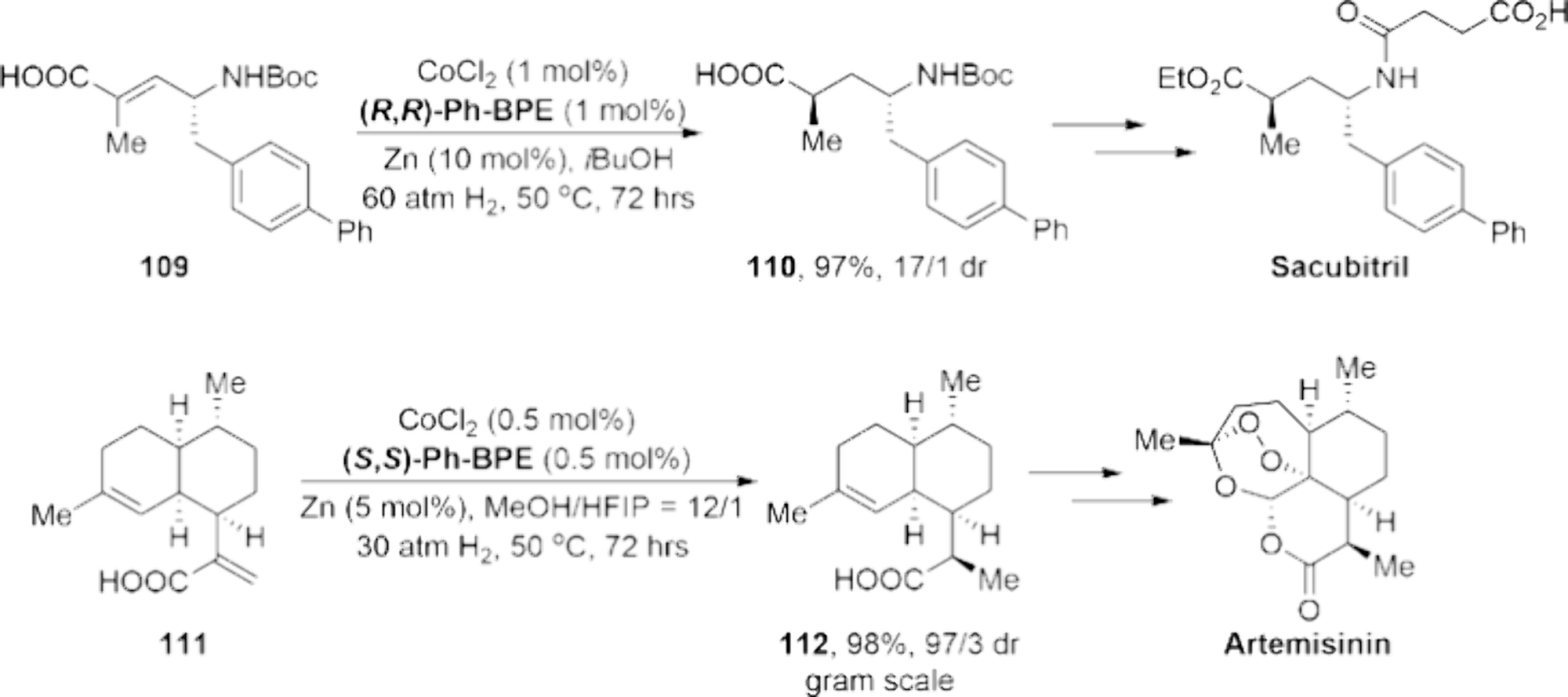
Synthesis of Chiral Drug Sacubitril and Artemisinin Intermediate 110 and 112 at Gram Scale

The other ligand that merits discussion with respect to the development of cobalt catalysis is the BPE ligand, which has shown great potential in cobalt-catalyzed asymmetric hydrogenations. One representative example takes advantage of a Zn-mediated activation method to produce hundreds of grams of the epilepsy medication levetiracetam. In 2018, Chirik and co-workers reported the combination of CoCl_2_·6H_2_O and (*R*,*R*)-Ph-BPE exhibited high catalytic activity and enantioselectivity in MeOH for the 200 g scale asymmetric hydrogenation of dehydro-levetiracetam **108**, affording levetiracetam in 97% isolated yield with 98.2% ee (Scheme 36).^113^ Importantly, the catalyst loading in this catalytic system could be reduced to ∼0.08 mol %, lower than the Rh catalyst loading (0.5 mol %) that has been reported.^114^ Stoichiometric studies established that the Co(II) catalyst precursor (*R*,*R*)-Ph-BPECoCl_2_ undergoes ligand displacement by methanol and zinc-promoted facile one-electron reduction to Co(I), which coordinated to the bisphosphine ligand more strongly. Another promising use of the Co-BPE catalyst system is in the asymmetric hydrogenation of α,β-unsaturated carboxylic acids **109** and **111** (Scheme 37).^115^ The gram-scale synthesis of key intermediates **110** and **112** toward enantioenriched drugs Sacubitril and Artemisinin, respectively, was thus achieved, with the catalyst showing high activity (up to 1,860 TON) and excellent enantioselectivity (up to >99% ee). Furthermore, Co(0)-((*R*,*R*)-Ph-BPE)(COD) has been effectively used for the hydrogenation of α,β-unsaturated carboxylic acids to synthesize chiral carboxylic acids, which are useful precursors to for example Naproxen and (*S*)-Flurbiprofen.^116^

Moreover, cobalt catalysis has also found applications in new fields, as represented by Isayama – Mizukaiyama cobalt catalyzed hydroperoxysilylation. This system has now been widely accepted as a superior process for aerobic epoxidation and hydroperoxysilylation of unactivated alkenes.^117^ Most recent, Chen and Xue summarized comprehensively the application of cobalt-catalyzed asymmetric reactions in total synthesis of natural terpenoids.^118^

### 2.4 Iron

**Scheme 38:**
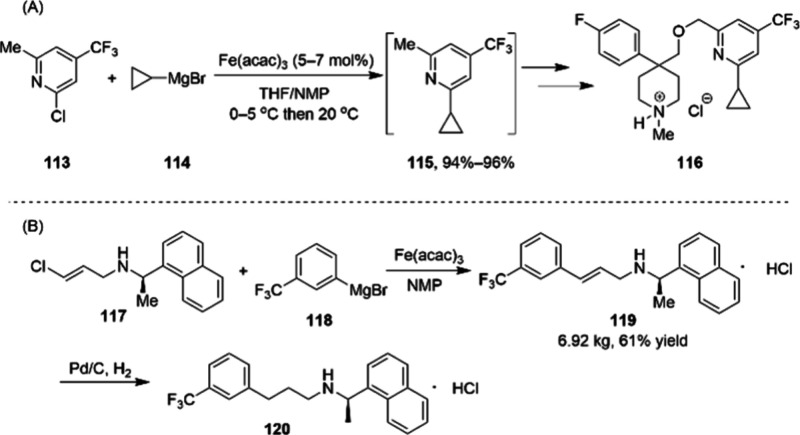
Large-Scale Synthetic Routes to 115 and 119 via Iron-Catalyzed Grignard Reactions

Despite being significantly cheaper ($144.90 mt) than other first-row transition metals (Ni: $13.845 lb, Cu: $4.2795 lb, Co: $37.195 lb), applications of homogeneous iron catalysts in industry are relatively sparse.^17^ This might be partially due to the instability of iron under catalytic conditions, as it tends to decompose into iron particles as the reaction progresses.^119^ In contrast, academic research on homogeneous iron catalysis has been increasing with recent reviews by Nakamura et al.^120^ and Ackermann et al. summarizing the state of play in iron-catalyzed C–H bond functionalization reactions.^121^ However, most large-scale applications in organic synthesis involving iron-catalysis are iron-catalyzed Kumada cross-coupling reactions, although palladium or nickel catalysts remain the most commonly employed metals in such chemistry. The driver here is most likely a combination of the broad utility of C–C bond-forming reactions in constructing pharmaceutical intermediates and the low toxicity and low cost of iron salts. In 2013, Conlon et al. reported the application of Fe(acac)_3_ in the multikilogram synthesis of heterocyclic dual NK1/serotonin receptor antagonist **97** (Scheme 38A).^122^ The process proceeds through iron(III)-catalyzed Kumada coupling of pyridyl chloride **113** and the corresponding Grignard reagent **114**, followed by benzylic chlorination utilizing trichlorocyanuric acid to selectively construct unsymmetrical 2,4,6-trisubstituted pyridine **115**. This route proved to be highly efficient. Moreover, a commercial kg-scale synthetic route for preparation of cinacalcet hydrochloride **120** (Scheme 38B), a calcimimetic agent and calcium-sensing receptor antagonist, was also described by chemists at Ranbaxy taking advantage of Fe(acac)_3_/*N*-methyl-2-pyrrolidone (NMP) catalyzed Grignard reaction of alkenyl halide **117** and the Grignard reagent *meta*-(trifluoromethyl)phenylmagnesium bromide (**118**).^123^ The synthetic approach involves C–C bond formation to prepare dehydrocinacalcet (**119**) followed by a hydrogenation reaction.

**Scheme 39:**
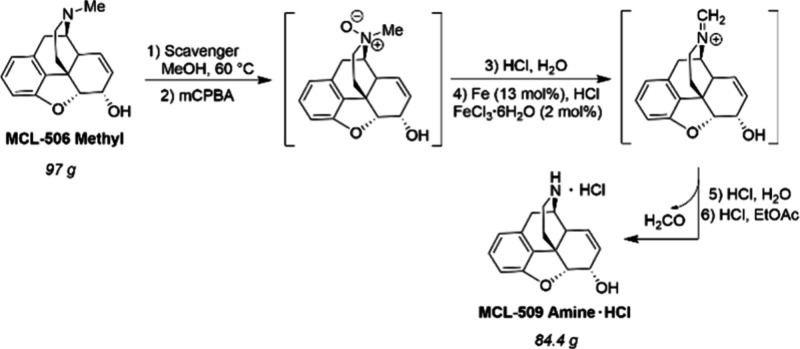
*N*-Demethylation of MCL-506 Methyl to MCL-509 Amine·HCl

The large-scale synthesis of MCL-509, a potential Parkinson treatment drug, integrates an iron-catalyzed nonclassical Polonovski reaction for the key *N*-demethylation (Scheme 39).^124^ This reaction was a great improvement in every aspect over alternative approaches that were explored, including a classical von Braun reaction and chloroformate/hydrazine-mediated demethylation employed in the initial discovery route.

**Scheme 40:**
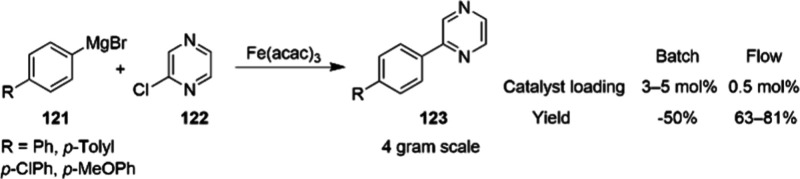
Iron-Catalyzed Kumada Cross-Coupling for the Synthesis of 123

**Scheme 41:**
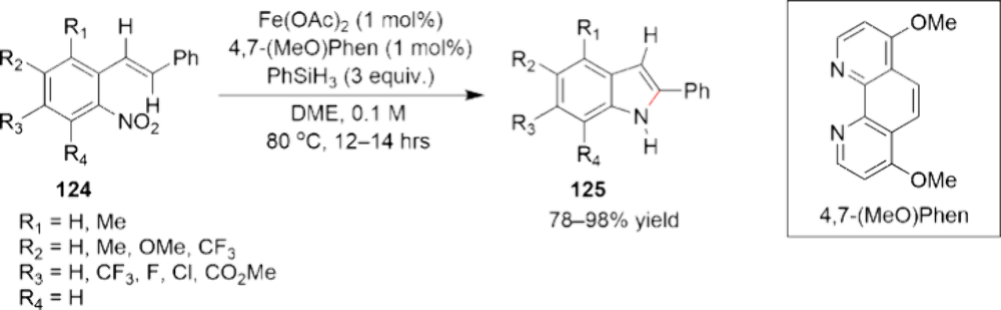
Milligram-Scale Iron-Catalyzed Transformation of Nitrostyrenes 124 to 125

Despite the growing number of successful applications, iron-catalyzed Kumada biaryl cross-coupling reactions still face multiple challenges in industry implementation, including catalyst deactivation, formation of homocoupling byproducts, limited substrate scope, and intolerance of some functional groups. Chemists at Boehringer Ingelheim developed a continuous flow process that solved these challenges and enabled an iron-catalyzed cross-coupling between 2-chloropyrazine (**121**) and aryl Grignard reagents **122** (Scheme 40).^125^ The continuous flow approach overcame the inherent exothermic nature of the reaction, thus enabling a longer catalyst lifetime and facilitating scale-up. Moreover, the yield of **123** was significantly improved by up to 30% in continuous flow mode compared to batch mode, and the catalyst loadings was decreased to 0.5 mol %. By utilizing a high-throughput experimentation approach, iron-catalyzed transformation of *ortho*-nitrostyrenes **124** into indoles **125** (Scheme 41) was described by scientists at Merck and University of Illinois at Chicago.^126^ The optimal reaction conditions require only 1 mol % of Fe(OAc)_2_ and 1 mol % of 4,7-(MeO)_2_Phen with phenylsilane acting as a convenient terminal reductant, producing indole derivatives (**125**) in moderate to high yield. Notably, this is a milder, catalytic, version of the Cadogan–Sundberg indole synthesis, which normally utilizes a stoichiometric amount of a trialkyl phosphite as reducing agent.

**Scheme 42:**
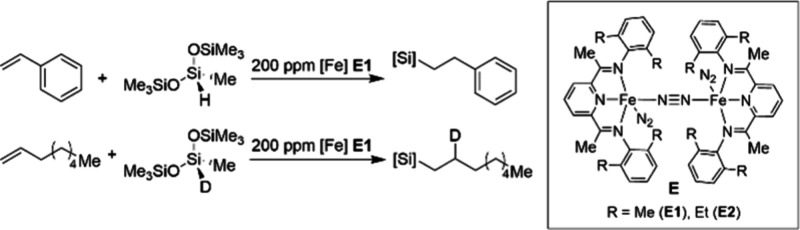
Iron-bis(imino)pyridine Complex-Catalyzed Hydrosilylation of Alkenes at Milligram Scale

It is worth mentioning that iron catalysts have also shown potential in hydrosilylation reactions. As shown in Scheme 42, in 2012 Chirik et al. reported a series of well-characterized iron complexes containing bis(imino)pyridine ligands (**E**) that promoted the regioselective anti-Markovnikov addition of sterically hindered, tertiary silanes to alkenes under mild conditions.^127^ The reaction was performed at milligram scale, and the iron complex showed high activity and offered broad functional group tolerance. The exclusive regioselectivity limited the formation of undesired stereoisomers to such an extent that the need for separation of unwanted byproducts may not be required in a potential industrial process. Iron is also able to mediate the hydrofunctionalization of alkenes by other mechanisms. In particular, iron-catalyzed hydrogen atom transfer (HAT) mediated intramolecular C–C coupling reactions between alkenes and nitriles has also been described.^128^ Utilization of PhSiH_3_ and catalytic Fe(acac)_3_ introduces a new strategic bond disconnection for ring-closing reactions, forming ketones via imine intermediates.

**Scheme 43:**
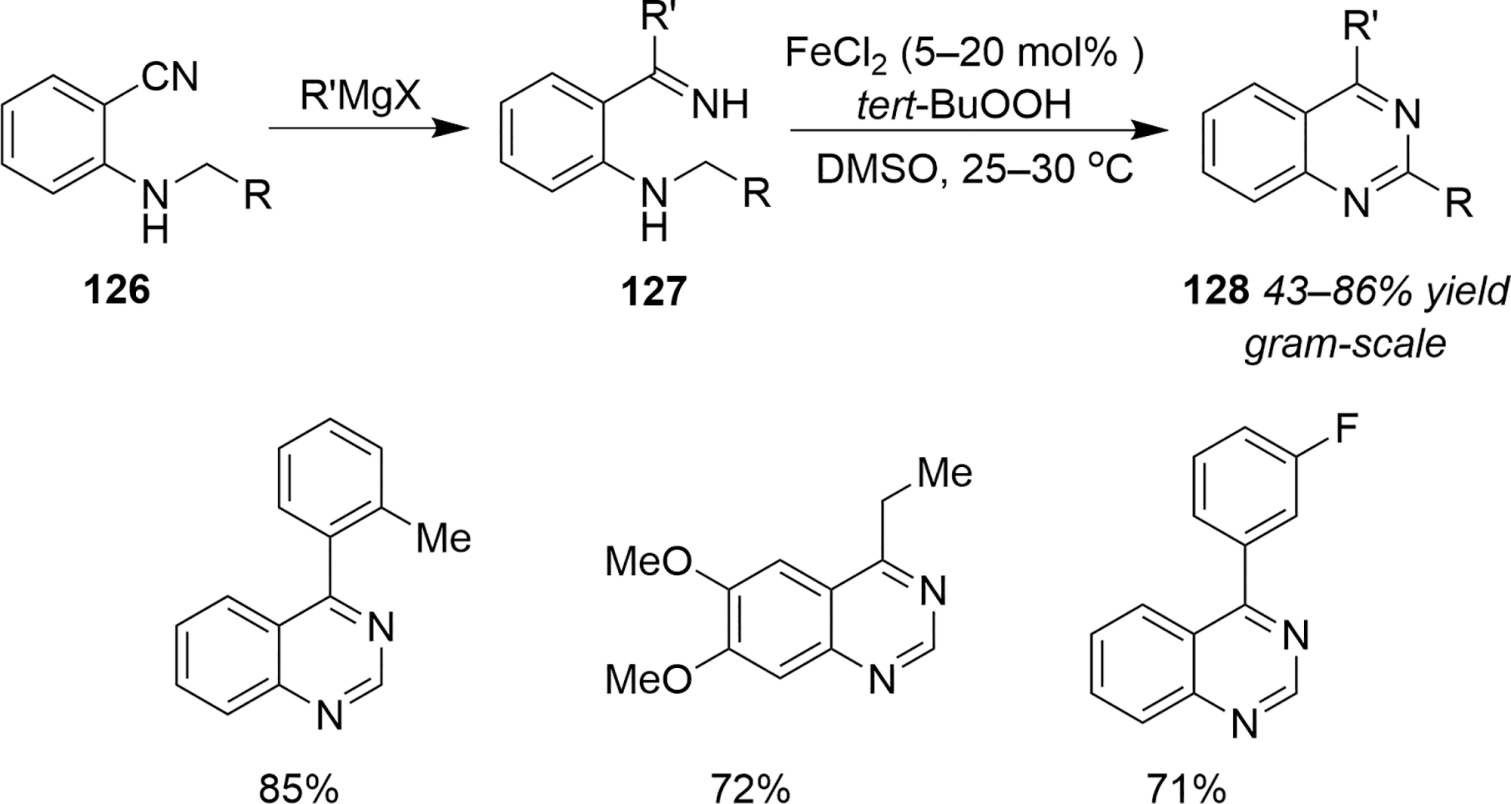
Iron-Catalyzed Synthesis of Quinazolines 128

Interestingly, iron-catalysis has also found utility in heterocycle synthesis. A collaboration between Janssen and Porton disclosed an efficient synthetic route to quinazolines (a ubiquitous class of compounds displaying a broad range of biological activities) based on iron-catalyzed C(sp^3^)–H oxidation and intramolecular C–N bond formation (Scheme 43).^129^ Readily available 2-alkylamino benzonitriles (**126**) were used as substrates and reacted with various organometallic reagents to produce 2-alkylamino NH ketimine species (**127**). Taking advantage of FeCl_2_ as the catalyst and *tert*-BuOOH as the terminal oxidant, C(sp^3^)–H oxidation of the alkyl group of **127**, followed by an intramolecular C–N bond formation and aromatization afforded a wide variety of 2,4-disubstituted quinazolines (**128**) in good to excellent yield. The gram-scale synthesis of a series of quinazoline derivatives demonstrated the applicability of this method in organic synthesis.

### 2.5 Manganese

**Scheme 44:**
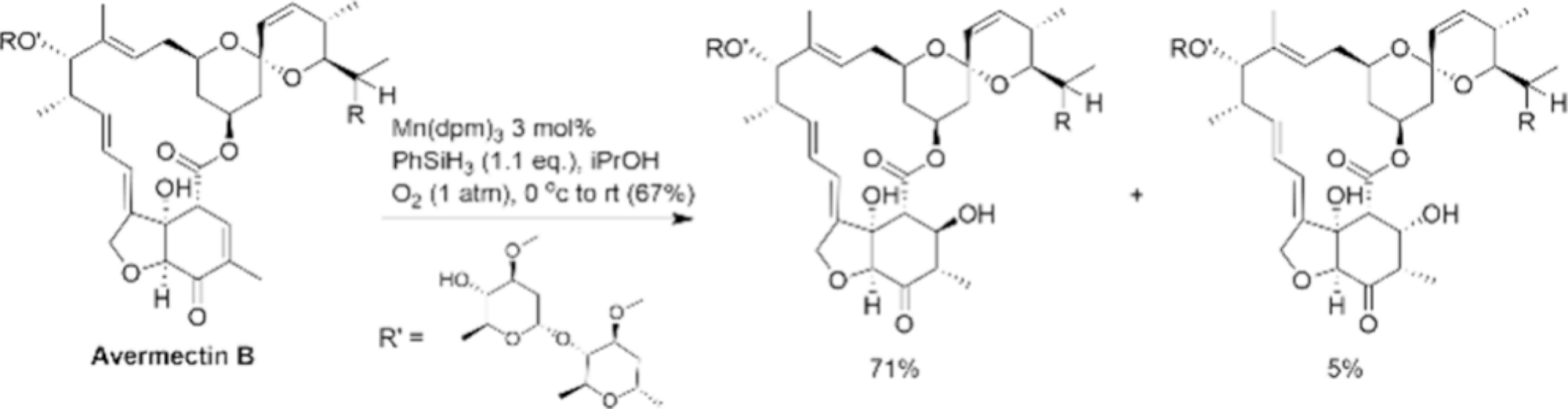
Manganese-Catalyzed Olefin Hydration Reactions for Gram-Scale Synthesis of Avermectin B Derivatives

In recent years, manganese catalysts have been explored as alternatives to the more commonly employed PGM catalysts in reactions such as C–H activation, hydrogenation and dehydrogenation reactions.^130,131^ Moreover, other reactions such as manganese-catalyzed three-component reactions of imines/nitriles^132^ and intramolecular nitrene transfer reactions have been investigated systematically.^133^ However, the application of manganese catalysis in industry is relatively limited. An early example is the application of (dipivaloylmethanato)-manganese(III) (Mn(dpm)_3_) in olefin hydration, which was initially developed by Mukaiyama et al. in 1990^134^ and extended by Magnus in 2000.^135^ Since then, the Mn(III)-catalyzed hydration reaction has been broadly accepted as a tool for total synthesis and natural product functionalization.^136^ A typical catalytic olefin hydration consists of phenylsilane in isopropyl alcohol under a dioxygen atmosphere in the presence of Mn(dpm)_3_ as a catalyst. Specifically, this Mn(III)-catalyzed hydration reaction was employed by chemists at Syngenta in the gram scale synthesis of avermectin derivatives (Scheme 44).^137^

Mukaiyama hydration is a representative member of a family of catalytic reactions that share a key metal hydrogen atom transfer (MHAT) elementary step. Another variant of this reactivity paradigm is MHAT hydrogenation. Manganese-catalyzed hydrogenation and hydrogen transfer reactions have been well reviewed by Sortais et al.^138^ In particular, one powerful manifestation of this concept from the Shenvi and Herzon research groups is the manganese- or iron-mediated hydrogenation reduction of C = C bonds.^139^ We expect to see future applications of this chemistry in large-scale reactions in due course.

**Scheme 45:**
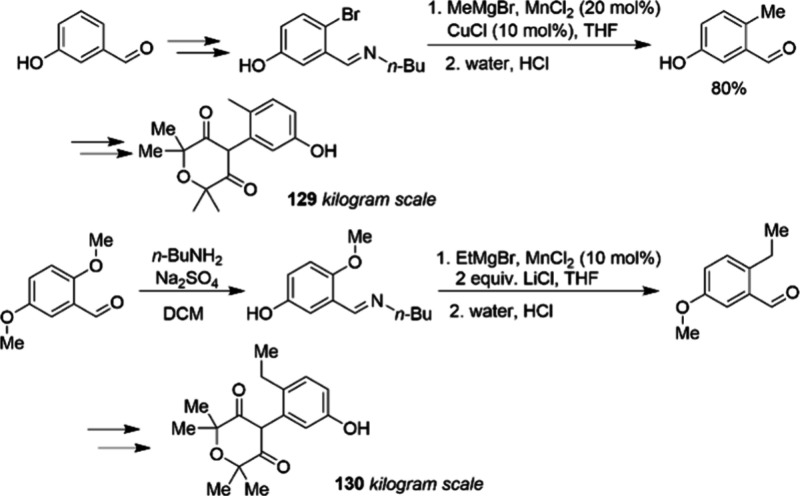
Kilogram-Scale Synthesis of aryl-1,3-Dione Motifs 129 and 130 via Mn–Cu-Catalyzed Alkyl Grignard Coupling

There are isolated examples of the use of manganese catalysts in an industrial setting, for example, the synthesis of aryl-1,3-diones **129** and **130**. The original synthetic route to access **129** was not suited to large-scale synthesis due to the involvement of an aryl lithium intermediate at −78 °C as well as the employment of an aryl lead reagent that is classified as highly toxic. While the aryl-1,3-diones turned out to be promising herbicidal acetyl-CoA carboxylase inhibitors, the successful realization of a kilogram-scale synthesis of these compounds was still uncertain. The solution was found in manganese catalysts by chemists at Syngenta who developed an alternative route employing a manganese–copper catalyzed coupling with alkyl Grignard reagents as the key step to synthesize aryl-1,3-dione motifs **129** and **130** (Scheme 45).^140^ The methodology could be applied at a kilogram scale for the synthesis of 2-alkyl substituted benzaldehydes and of 2-aryl-1,3-diones.

**Scheme 46:**
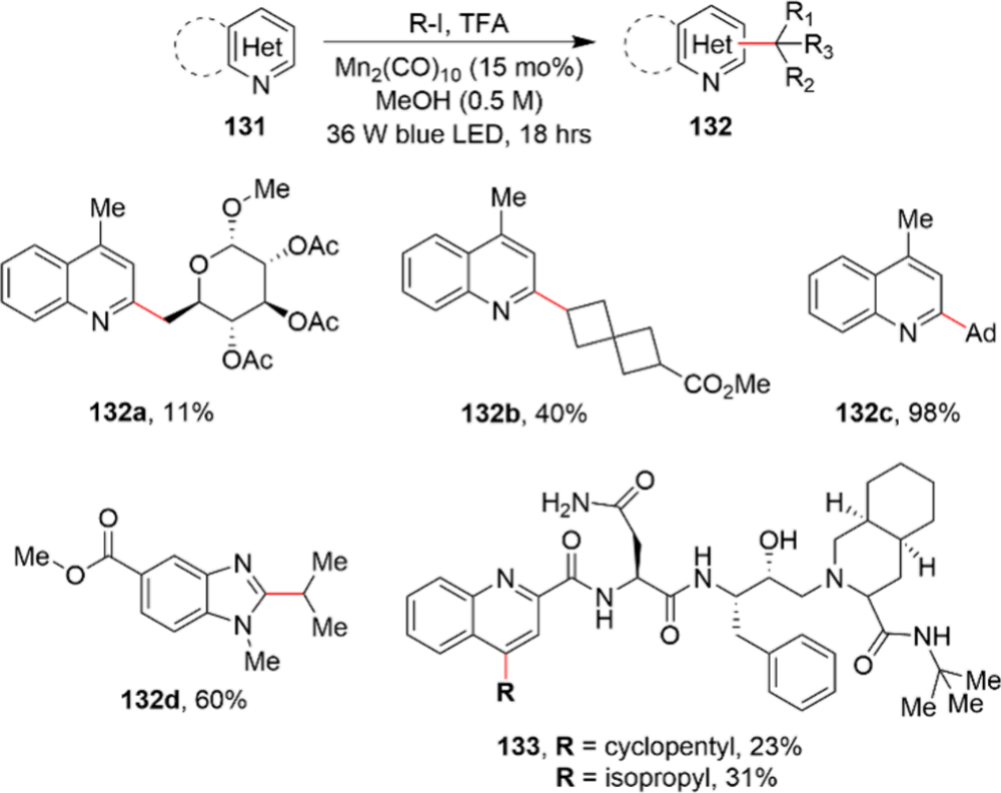
Milligram-Scale Manganese-Catalyzed Photomediated Minisci Reaction of Quinoline Derivatives 131

In an effort to leverage the catalytic activity of Mn_2_(CO)_10_ in an emerging class of reactions, chemists at Pfizer developed a visible-light-driven Minisci protocol without using precious metal catalysis, such as iridium, ruthenium, or silver.^141^ As shown in Scheme 46, this protocol was reported to be compatible with various functional groups such as sugar moieties (**132a**), spirocycles (**132b**), and others. No peroxide was needed in this photomediated Minisci method. Although these reactions were performed at mg scale, the use of a manganese based catalyst provided an economic benefit. This protocol represents an alternative method for the functionalization of complex nitrogen-containing drugs, as demonstrated in the case of two representative products (**133**). In this case, late-stage C–H alkylation with either acyclopentyl or isopropyl groups proceeded in 23% and 31% yield, respectively.

**Scheme 47:**
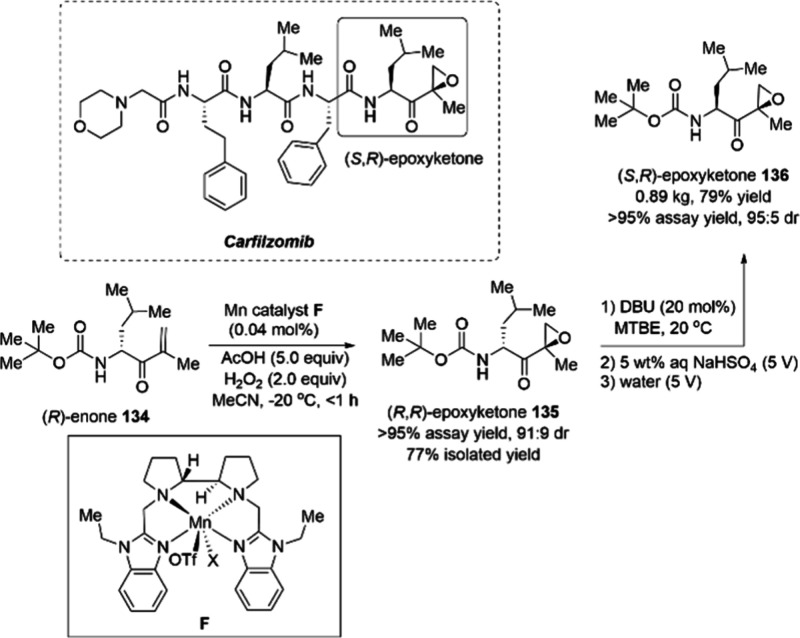
Manganese-Catalyzed Commercial Route to Intermediate 136 of Carfilzomib

The development of advanced ligands has also fueled the application of manganese catalysts in drug synthesis. Carfilzomib, also known as Kyprolis, is a proteasome inhibitor initially approved by FDA in 2012.^142^ According to the public manufacturing routes to Carfilzomib, epoxyketone moiety **136** is a key intermediate. In a recent report by chemists at Amgen, a commercial-scale route to (*S*,*R*)-epoxyketone **136** was disclosed.^143^ As shown in Scheme 47, central to this approach was the development of a kilogram-scale manganese-catalyzed asymmetric epoxidation method to (*R*,*R*)-epoxyketone **135**, using hydrogen peroxide as the stoichiometric oxidant and (R)-enone **134** as the starting material. Critical to the successful isolation of **136** is a solubility screen identified IPA/water as the ideal crystallization system to reject the undesired diastereomer so that the aid of column chromatography is not required. (*R*,*R*)-Epoxyketone **135** was isolated in 77% yield containing <0.5% epoxide diastereomer. Epimerization of the leucine side chain in the presence of 20 mol % DBU resulted in (*S*,*R*)-epoxyketone **136**. Subsequently, the kilogram-scale manufacture exploited an analogous NMP/water seeded-batch coaddition crystallization procedure. Eventually, **136** was obtained with ≥95:5 dr and 98.6 LCAP. This methodology was able to address the challenges associated with the existing bleach epoxidation process and eliminate the requirement for column chromatography.

**Scheme 48:**
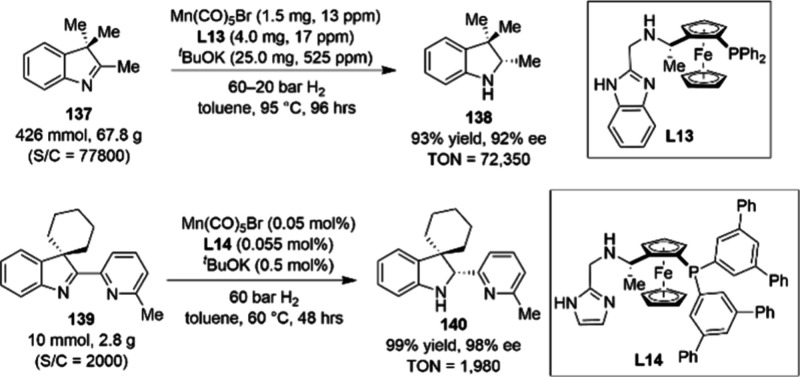
Mn-Catalyzed Decagram-Scale Asymmetric Hydrogenation of *3H*-Indole Derivatives 137 and 139

Recently, asymmetric hydrogenation (AH) reactions of unsaturated compounds via manganese-catalysis were also investigated by Liu et al.^144^ Through the use of chiral NNP-pincer-ligand (L13 or L14) coordinated to manganese, AH of *3H*-indoles with excellent yields and enantioselectivities was achieved (Scheme 48). This methodology expands the scope of AH to substrates which are unsuccessful using a state-of-the-art ruthenium catalyst. The reaction could proceed with catalyst loadings at the ppm level with an exceptional turnover number of up to 72,350. This is the highest value yet reported for an earth-abundant metal-catalyzed AH reaction.^141b^

### 2.6 What about Other Metals?

In addition to the metals discussed in separate sections above, there are a couple of other non-PGM metals that merit highlighting in this review.

There are several reaction classes for which zinc complexes could be potential catalysts. To mention a few; reduction and oxidation chemistry and carbon dioxide functionalization. A nice perspective article by Enthaler provides a reference for those who may want to read more.^145^ However, to the best of our knowledge, the application of zinc catalysts has yet to be reported in large-scale or manufacturing applications. This could be due to many reasons, for example, a lack of understanding, and hence control, of the reproducibility of such reactions or lack of access to the required catalysts in sufficient quantity.

Zinc reagents can not only act as catalysts for certain transformations, but there are also a number of reactions in which they can be useful stoichiometric reagents. One prominent example of this is of course Negishi coupling reactions, where an organozinc species is coupled with an alkyl or aryl (pseudo)halide. Although this is not the main focus of this article, one specific report by researchers at Pfizer and Snapdragon is nevertheless noteworthy. During the development of a clinical stage active pharmaceutical ingredient, the team identified the zinc complex [(DMPU)_2_Zn(CF_2_H)_2_] as an extremely effective reagent for a selective and high-yielding difluoromethylation reaction. However, they were not able to source this required zinc complex in sufficient quantities to progress the development of this difluoromethylation process. They therefore set out to develop a continuous flow process that could deliver this reagent on larger scale, a task that was successfully accomplished.^146^ There are reports of the use of this reagent in nickel or copper catalyzed difluoromethylation reactions,^147−149^ and we eagerly await the authors’ coming report on how they subsequently achieved this transformation in their API synthesis project.

**Scheme 49:**
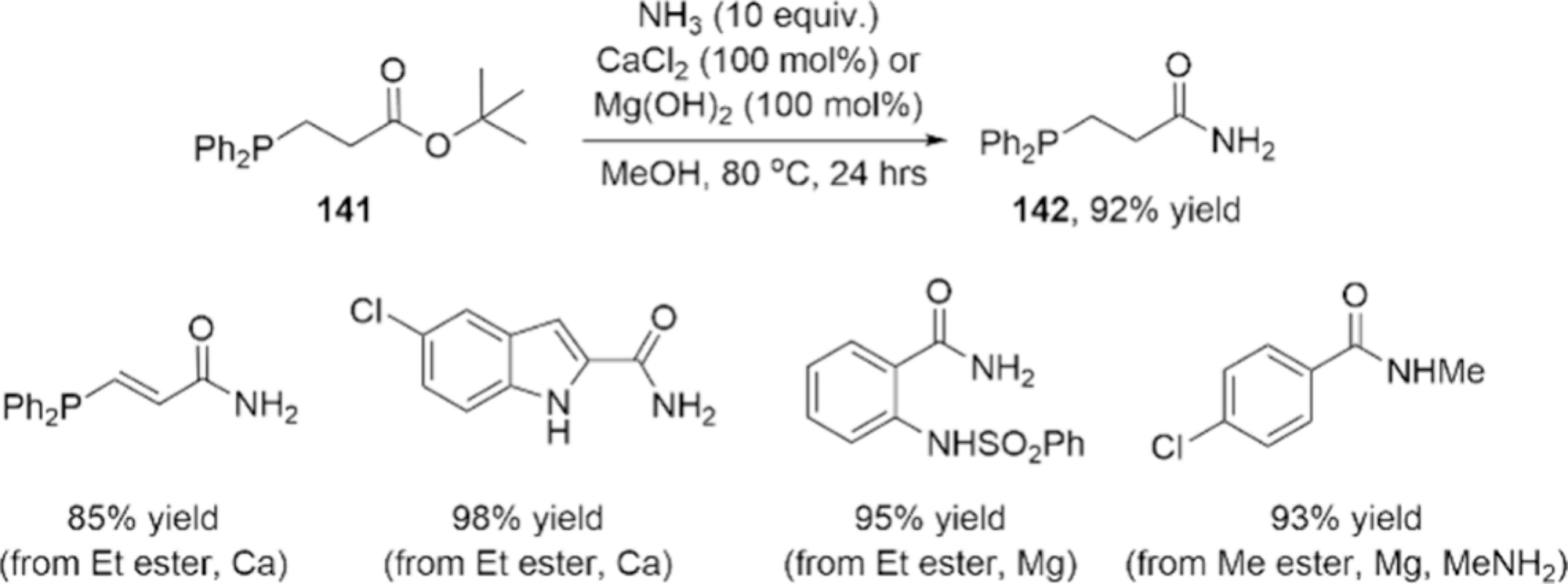
Amidation of Esters with Ammonia Promoted by Low-Cost and Low-Toxicity Non-PGMs

Finally, we note that in certain cases, the extremely low cost and low toxicity of certain non-PGM compounds can render them highly effective “catalysts” even when they are used in stoichiometric quantities. One such example is the report by scientists at Pfizer of a calcium- or magnesium-promoted amidation of esters with ammonia.^150^ A screen of readily available Lewis acids showed that CaCl_2_ and Mg(OMe)_2_ were highly effective at promoting this industrially relevant transformation. The reaction shows remarkable chemoselectivity and can be extended to alkylamines (Scheme 49). A simple aqueous workup was sufficient to remove the magnesium or calcium salts that formed. While reported on a small scale in the Pfizer article, these conditions should be readily scalable due to the advantages mentioned above. The use of Mg(OMe)_2_ for an amidation with dimethylamine was subsequently described on 10 kg scale in a patent (US8680280B2).

Mg catalysts have been shown to benefit from being used in conjunction with similar ligands to those employed in PGM catalysis.^151^ A nice review published by Kwit et al. comprehensively summarized stereoselective transformations that take advantage of magnesium with the advanced ligands employed in PGM catalysis.^152^ Several Mg-catalyzed transformations have been reported in small-scale efforts, and we are eagerly on the lookout for applications of these reactions in industry: catalytic hydroboration of alkyl- and aryl-substituted carbodiimides with pinacol borane (HBpin);^153^ enantioselective Friedel–Crafts alkylation reactions;^154^ asymmetric ring-opening reactions^155^ and chemoselective reduction of α,β-unsaturated carbonyl compounds.^156^

## 3 CONCLUSION AND OUTLOOK

The number of reaction classes in which non-PGM metals can be employed in place of or in preference to PGMs is undoubtedly on the rise and will keep increasing.

The evaluation of the state of play in non-PGM catalysis reveals several benefits across the entire drug development process from the delivery of the first few grams to large-scale manufacturing. Non-PGM catalysts can, for example, provide reactivity and substrate scope complementary to those of their PGM counterparts. However, challenges remain with respect to robustness and reproducibility upon scale-up. For example, nickel catalysts are more prone to substrate inhibition and undesired side reactions such as protodehalogenation or protodeborylation. Applications of non-PGM catalysis on process scale remain limited in number, most likely due to a combination of factors: a) lack of understanding of reaction mechanism and kinetics of reactions catalyzed by non-PGM as opposed to PGM catalysts; b) reaction conditions that are challenging to scale up (e.g., heterogeneous reaction conditions, carefully control of water stoichiometry); c) lack of commercial availability of the required non-PGM catalysts on the large quantities required. Further collaboration across academic laboratories, pharmaceutical process development groups, and catalyst and ligand manufacturers is crucial to the successful advancement and introduction of more non-PGM catalyzed processes in large-scale applications. Non-PGM catalysis will most likely never entirely remove the need for PGM catalysts; however, an increasing understanding and evolution within the non-PGM field will ensure that the industrial chemist has a larger number of tractable options available when route scouting or process optimization.

On writing this article, one extremely important, general, observation shines through the entire story: in addition to the use of more sustainable metal catalysts, the R&D chemist (and indeed R&D managers) would be encouraged to look for solutions from outside of the pharmaceutical field. Non-PGM (base metals as well as first-row transition metals) catalysts have been used on large scale in other industries for many years. We can see a parallel between the earlier adoption of electrochemistry in the fine chemical industry (e.g., the Monsanto adiponitrile process, 1965) and the recent exploration of this technology for pharmaceutical processes.^157^ We encourage readers in the pharmaceutical development community to look to other industries (e.g., fine chemicals, material science, polymers) for inspiration in developing new applications of non-PGM catalysis. It is never too late to start, and one never knows what one will find.
